# Multiomic analysis identifies glutaminolysis-dependent metabolic enhancement of immune memory utilised for vaccine development

**DOI:** 10.1038/s41467-026-74866-4

**Published:** 2026-06-26

**Authors:** J. M. Sowerby, P. Kotagiri, S. Clare, D. Griffiths, O. Knight, I. Cuthbertson, K. Harcourt, P. Pereyra Gerber, I. Pshenichnaya, C. Connor, A. Denton, G. A. Stoll, C. Matara, C. Keel, G. Manferrari, T. Birkle, P. Naydenova, H. Peck, P. A. Lyons, I. Barr, Y. Modis, G. Dougan, N. J. Matheson, K. G. C. Smith, E. F. McKinney

**Affiliations:** 1 Cambridge Institute of Therapeutic Immunology and Infectious Disease, Jeffrey Cheah Biomedical Centre, Cambridge, UK; 2 Department of Medicine, University of Cambridge School of Clinical Medicine, Cambridge, UK; 3 Wellcome Sanger Institute, Wellcome Genome Campus, Hinxton, Cambridge, UK; 4 Molecular Immunity Unit, Department of Medicine, University of Cambridge, MRC Laboratory of Molecular Biology, Francis Crick Avenue, Cambridge, UK; 5 National Institute for Health Research Cambridge Centre for Clinical Research, Cambridge, UK; 6 WHO Collaborating Centre for Reference and Research on Influenza (VIDRL), Peter Doherty Institute for Infection and Immunity, 792 Elizabeth Street, Melbourne, Australia; 7 NHS Blood and Transplant, Cambridge, UK; 8 Cambridge Centre for Artificial Intelligence in Medicine, Cambridge, UK

**Keywords:** Drug development, Molecular medicine, Experimental models of disease, Vaccines

## Abstract

Vaccines work by inducing an immunological memory response to protect against infection or disease. More effective vaccines are required to improve protection of high-risk groups such as the elderly. Here we screen transcriptional signatures of T cell memory against repurposable drug signatures, identifying a subclass of lysine deacetylase inhibitors (KDACi) capable of promoting a memory precursor phenotype in primary T cells. Leveraging combined acetylomic, metabolomic, transcriptomic and epigenomic analyses, we identify enhanced glutaminolysis as the mechanism responsible for the KDACi effect, with selective inhibition reversing the changes induced by KDACi treatment. We validate the ability of a repurposable KDACi (sodium valproate) to promote immune memory in four murine models of infection and immunisation, with the effect reversed by concurrently inhibiting glutaminolysis. Finally, we undertake a human challenge study, which demonstrates a 10-fold increase in correlates of protection following seasonal influenza vaccination. These results reveal immune-metabolic adjuvant properties of repurposable lysine deacetylase inhibitors and demonstrate that systematic screening can identify both efficacy and mechanism of repurposable drugs.

## Abstract (web summary)

Drug-repurposing could provide optimised vaccine adjuvants while avoiding lengthy toxicity testing. Here authors perform multi-omics screening of T cell phenotypes using a library of repurposable drugs to find that a subclass of lysine deacetylase inhibitors promote a memory precursor phenotype in primary T cells via enhanced glutaminolysis, and vaccines adjuvanted with these drugs provide superior protection in mouse and human challenge studies.

## Introduction

Lasting protection after vaccination or infection depends on the induction of durable memory responses^1^. Enhancing immune memory could increase vaccine efficacy as greater memory responses associate with reduced disease incidence and severity, for example, in SARS-CoV-2^2^ and influenza^3^. Adjuvants are vaccine components that aim to enhance the size, breadth and durability of a vaccine response. Current adjuvants primarily rely on the provision of innate ‘danger’ signals^4^, although an understanding of their mechanism has lagged well behind discovery^5^. More recently, a systematic approach to vaccine development and design has been proposed^6^ using genomics to better understand vaccine responsiveness and guide adjuvant design^7^.

Drug repurposing—the use of failed or approved drugs with established safety profiles for new indications –creates the potential to rapidly translate treatments for new indications^8^. Many drugs never reach clinical testing and <15% in development receive clinical approval despite the majority being safe for use^9^. Although successful examples of clinical repurposing exist^10^, most are serendipitous rather than a result of screening^8^.

In this study, we screen transcriptional signatures of T cell memory and exhaustion against drug response signatures and test the ability of predicted hits to alter T cell memory differentiation using a primary human in vitro model. We use a combination of acetylomics, metabolomics, transcriptomics and functional metabolic assays to identify enhanced glutaminolysis during T cell activation as the immunometabolic mechanism responsible. We then test the ability of a repurposed KDACi to promote memory expansion using four mouse models of infection or immunisation, including a rechallenge model where treatment had only been given during primary exposure. Finally, we validate the ability of sodium valproate (a repurposable KDACi) to enhance both cellular and antibody responses to seasonal influenza vaccination in healthy human volunteers. Our results demonstrate that genomic analysis can be used to systematically identify repurposable drugs while informing their mechanism of action and confirm efficacy of a repurposed immunometabolic adjuvant boosting vaccine induced immunity.

## Results

### A subclass of KDACi modulates a memory phenotype in vitro

**Fig. 1:**
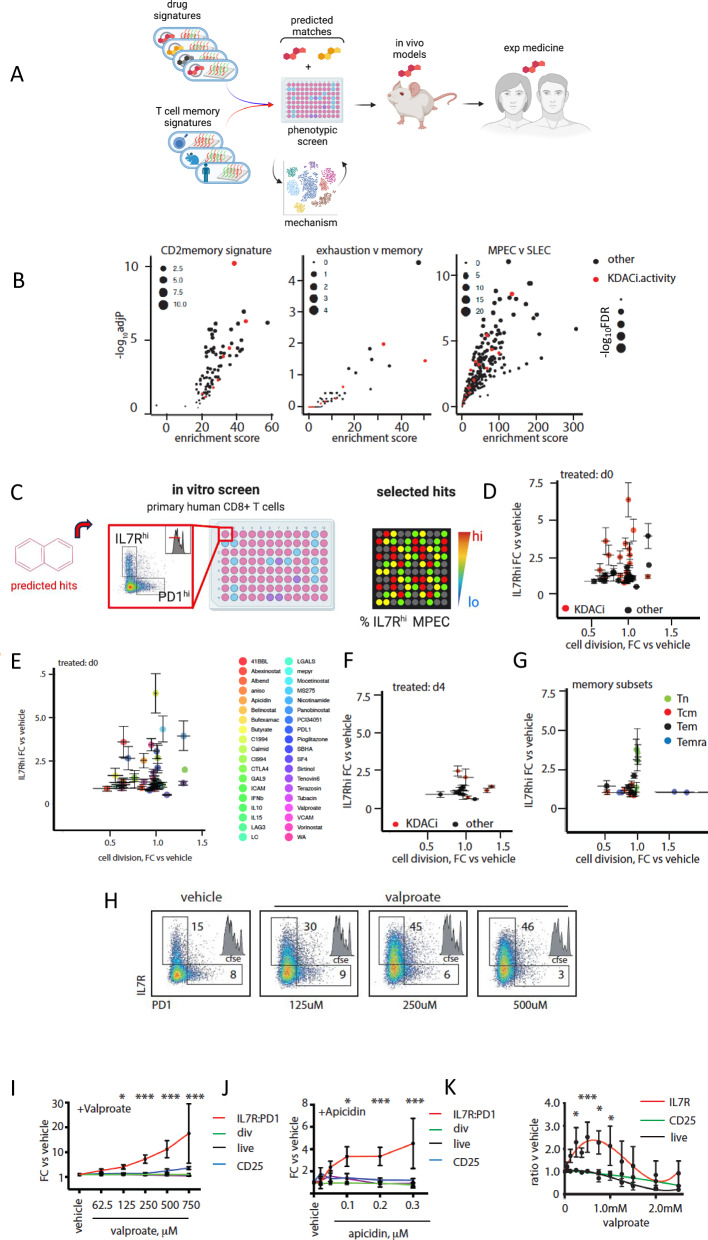
In silico screening identifies a subclass of KDACi modulating a memory phenotype in vitro. **A** Schematic illustration of transcriptomic approach to small molecule repurposing. Created in BioRender. McKinney, E. https://BioRender.com/obzf3is (**B**) Bubble plots showing enrichment score (*x*-axis) against significance (*y*-axis) for matching of three CD8^+^ T cell memory signatures against drug response signatures collated from GEO (SuppData1, methods). Red symbols indicate compounds with KDACi activity, *n* = 700 drug signatures screened against each memory signature. Plots illustrate those with overlap of at least *n* = 5. -log_10_FDR of enrichment. **C** Schematic illustration of in vitro phenotypic screen, created in BioRender. McKinney, E. https://BioRender.com/x211kuy**D**–**G** Scatter plots showing maximum change in MPEC (IL7R^hi^ %, FC v vehicle) against % cell division (*x*-axis, FC v vehicle) for each of 42 selected treatments (**D**, **E**: *n* = 844, mean +/-sem), for 13 selected treatments (**F**) given 4 days after stimulation, and for 6 selected KDACi given to isolated T cell subsets (**G**). All treatments given to highly purified, sorted T cell memory subsets and timepoints as indicated. **H** Representative scatterplots illustrating dose-dependent induction of an MPEC phenotype (IL7R^hi^PD1^lo^) on valproate treatment. Inset numbers indicate % population, inset histograms indicate proliferation (cfse dilution). **I**–**K** Line and scatterplots showing induction of MPEC phenotype (FC vs vehicle) on treatment with low dose valproate (**I**, *n* = 8) or apicidin (**J**, *n* = 5). **K** Biphasic dose response with higher dose valproate exposure. **I:** **K** y-axes, ratio v vehicle. red, IL7R:PD1 ratio, green = cell division, blue = activation (CD25^+^MFI), purple=cell survival (%live). 2 sided Mann Whitney test *<0.05, **<0.01, ***<0.001. MPEC memory precursor effector cell, SLEC short lived effector cell, FC fold change.

To identify existing small molecules with the potential to modulate T cell memory responses we undertook an in silico repurposing screen. We matched three independent transcriptional signatures (Supplementary Data 1) comparing CD8^+^ T cell memory cells to their short-lived effector or exhausted counterparts (Fig. 1A, B^11–13^) against three libraries of small molecule transcriptional response signatures comprising over 30,000 compounds (GEO, LINCS and the connectivity map (CMap), Methods, Fig. 1B^14^). Drug response signatures were primarily derived from transformed cell lines, although many gene co-expression programmes are shared between species^15^, tissues and cell types^16^ for example those regulating metabolic signalling and multipotency^17^. We anticipated that screening signatures generated in unmatched cell lines could therefore identify candidate compounds targeting shared pathways.

Amongst top predicted hits we observed multiple instances of compounds with lysine (K) deacetylase inhibitory (KDACi) activity (Fig. 1B). Forty two treatments, including compounds matching screened libraries^18,19^ (adjusted P < 0.05 with a minimum of 2 enriched signatures per drug class, Supplementary Data 2, 3) and those known to impact memory differentiation were considered for validation in an in vitro phenotypic screen (Supplementary Data 2). In the in vitro model, reciprocal expression of memory precursor (IL7R^20^) and markers of activation/exhaustion (PD1^21^) on stimulated primary human CD8^+^ T cells (Fig. 1C, polyclonal iBEAD, anti-CD2/CD3/CD28) is used to identify memory precursor effector cell (MPEC) differentiation^20^ including gene expression changes used for screening^13^. Amongst 844 assays, screening 3 doses for each of 42 treatments, we observed significant expansion of IL7R^hi^ MPEC following treatment with multiple KDACi with no effect on cell proliferation (CFSE^mfi^), survival (liveAqua^neg^) or cell activation (CD25^mfi^, Fig. 1D, E). T cell memory fate is known to be driven by a differentiation programme, initiated within hours of antigen encounter and independent of later signals^22–24^. Consistent with this, induced memory differentiation diminished if treatment was delayed following stimulation (Fig. 1F) and was observed to arise predominantly from differentiation of naïve T cells, rather than from restimulation of those in a pre-existing memory pool (Fig. 1G) with a similar effect seen in isolated stimulated CD4^+^ T cells (Figure S1I). Together, these data show that combined in silico and phenotypic screening identifies a subset of compounds with KDACi activity capable of modulating markers of memory T cell differentiation.

Lysine acetylation is a reversible post-translational modification controlled by the opposing activity of acetyltransferases (KATs) and deacetylase (KDAC) enzymes^25^. Initially termed histone deacetylases (HDAC), the advent of global acetylation mapping revealed acetylation targets outside the nucleus, including in the cytoplasm and mitochondria^26^. Widespread roles for KAT/KDAC are apparent in cellular signalling, including regulation of protein enzymatic activity, subcellular localisation and metabolic pathway regulation^27^. The 18 known human KDAC enzymes fall into 4 main classes (I-IV), and the effects of inhibitors differ widely by target KDAC specificity^27^, functional impact measured^26^ and target cell type^28^. Promotion of growth arrest or death of transformed cells has resulted in the development of some KDACi as cancer therapeutics^29^, and some have also been reported to modulate immune cell differentiation and function^30–32^ but not immune memory. Following KDACi treatment of primary human CD8^+^ T cells, we observed a biphasic dose response, with MPEC expansion occurring only at low doses at which no impact on cell survival, proliferation or activation was seen (Fig. 1H-K). Higher doses limited cell proliferation and survival consistent with their clinical use in the context of cancer (Fig. 1K, Figure S1J, Supplementary Data 3)^33^.

### Enhanced glutaminolysis promotes differentiation of a T cell memory phenotype in vitro

**Fig. 2:**
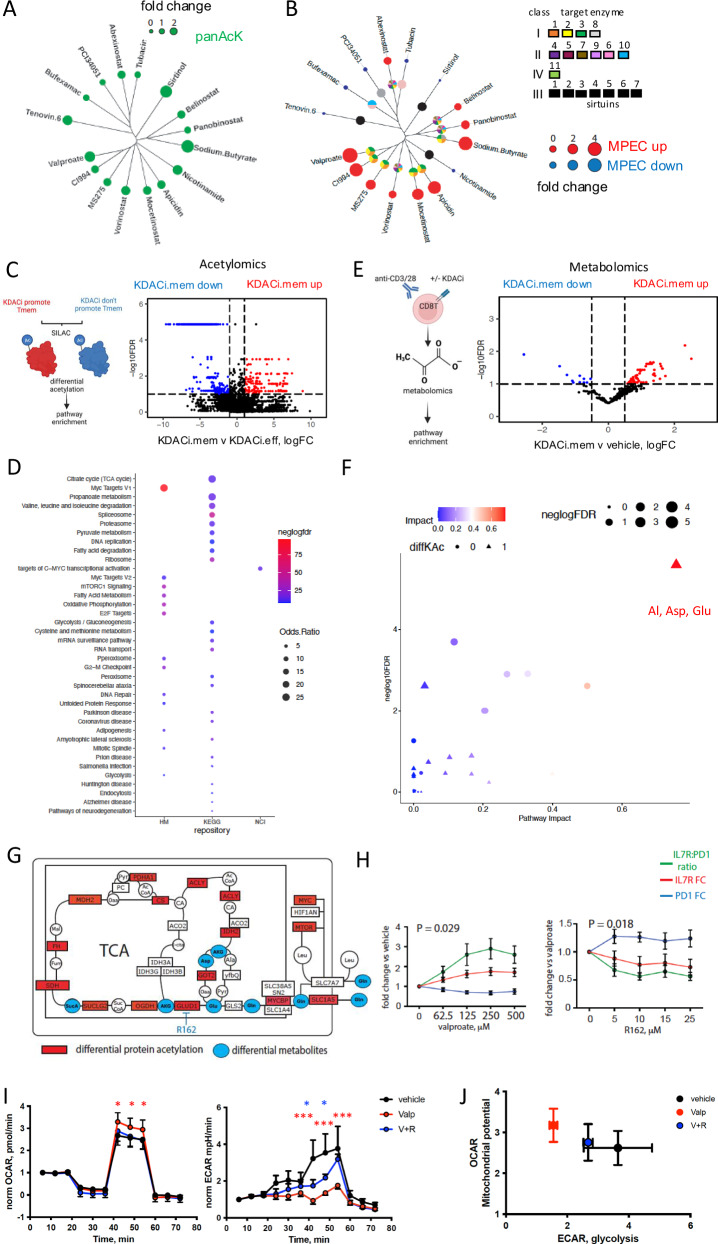
Acetylomic and metabolomic analysis pinpoints enhanced glutaminolysis as the immunometabolic mechanism. **A**, **B** Unrooted tree plots showing KDACi compounds clustered by site-specific acetylomic changes induced on treatment of HEK cells. Leaf diameter indicates total acetylation (**A**, panAcK FC v vehicle) or MPEC expansion (**B**, FC v vehicle) on treatment of stimulated primary CD8^+^T cells with KDACi as indicated. Internal pie charts (**B**) indicate target KDAC enzyme class(es) of each compound. **C** Schematic illustration (left, SILAC stable isotope labelling by amino acids in cell culture) and volcano plot (right) showing differentially acetylated proteins between KDACi.mem and KDACi.eff compounds in treated HEK cells. **D** Bubble plot showing pathway enrichment analysis of *n* = 1544 differentially acetylated proteins (*n* = 230 up, *n* = 1314 down) identified in (**C**) against each of three pathway repositories (HM: Hallmark, KEGG and NCI-PID), created in BioRender. McKinney, E. https://BioRender.com/e5up9ym (**E**) Schematic illustration (left) and volcano plot showing differential metabolomic analysis of CD8^+^T cells d6 after stimulation with KDACi (valproate/apicidin, *n* = 16) or vehicle (*n *= 8). **F** Scatter plot showing pathway enrichment analysis of differential metabolites (*n* = 68, FDR < 0.05) identified in (**E**). Pathway impact (*x*-axis) is a topological measure of metabolite importance in a network. Symbol shapes indicate whether each pathway also showed significant (FDR < 0.05) differential acetylation (from **D**). **G** KDACi (valproated/apicidin) induced metabolites (green, from C) and acetylated proteins (red, from **E**) mapped onto the glutaminolysis pathway (HMDB SMP02298). (H) Line and scatter plots (mean + /-sem) showing MPEC differentiation (IL7R^hi^PD1lo FC vs vehicle) on treatment (d0) with 125μM valproate (left, *x*-axis, *n* = 8) or with valproate + increasing doses of the glutaminolysis inhibitor R162 (right, *x*-axis, *n* = 5). *P* = 2 way ANOVA. **I**, **J** Metabolic flux analysis showing baseline-normalised oxygen consumption rate (OCR, left) and extracellular acidification rate (ECAR, right) against time for CD8^+^ T cells stimulated with valproate (red), vehicle (black) or valproate + R162 (green), *n* = 5, mean + /-sem. 2 sided Mann Whitney test *<0.05, **<0.01, ***<0.001.

Only a subset (9/16, 56%) of KDACi tested (Figure S1J) drove dose-dependent IL7R^hi^PD1^lo^ MPEC differentiation (Fig. 1I-J, KDACi.mem), while others had no effect on effector phenotype (KDACi.eff, Fig. 2A and Figure S2). To understand the mechanism responsible, we investigated patterns of protein acetylation resulting from exposure to each. Global acetylation levels within a cell can act as a metabolic signalling intermediate^34^, allowing cells to modulate survival and proliferation according to available resources. As expected, treatment of primary human CD8^+^ T cells with all KDACi resulted in increased total cellular acetylation, although no difference was observed on treatment with KDACi.mem and KDACi.eff groups (Fig. 2A). To explore a role for differential acetylation of specific peptides, we analysed mass spectroscopic acetylomic data from HEK cells treated with a range of KDACi (including both KDACi.mem and KDACi.eff)^26^. Unsupervised clustering of acetylomic data from KDACi-treated HeLa cells segregated compounds by their effect on MPEC differentiation, but not by either target KDAC enzyme class (Fig. 2B, inlaid pie chart) or total acetylation level induced (Fig. 2A, B) suggesting marked differences in protein aceylation between the groups. Comparing each, we identified 1560 differentially acetylated sites from 1116 proteins (Fig. 2A, C, threshold fdr 5%). Pathway enrichment analysis against multiple repositories (Fig. 2D, Hallmark, KEGG and NCI-PID) indicated over-representation of metabolic pathways (Supplementary Fig. 2B, C), most strongly for the TCA cycle and Myc signalling (Fig. 2D), a known master regulator of metabolic reprogramming in activated T cells^35^.

Given this we undertook metabolomic analysis of primary human CD8^+^ T cells stimulated in the presence of sodium valproate and apicidin, KDACi.mem with highest potency and potential for repurposing (Figure S1J, Supplementary Data 3, Fig. 1H–K). Sixty-eight metabolites were differentially abundant (FDRq<0.1) compared to vehicle-treated, stimulated control cells (Fig. 2E, and Supplementary Data 4, 5). Metabolite pathway enrichment analysis^36^ identified Alanine, Aspartate and Glutamate (Al/Asp/Glu) metabolism^36^(FDR < 5%, Figure S2D, E) and glutaminolysis in particular (Fig. 2G) as the central pathway being modulated. Glutaminolysis feeds metabolic intermediates into the TCA cycle through generation of α-ketoglutarate (αKG), generating energy and biosynthetic precursors such as nucleic acids while modulating cellular redox balance, growth and survival in a process termed ‘anaplerosis’^37^. It makes an important energetic contribution to T cell activation^38^, and is known to fuel stem cell-like oxidative metabolism in cancer cells^39^, sustaining chronic proliferation, growth and survival and making it a target of cancer therapy^40^. The combined acetylomic and metabolomic analyses may similarly facilitate stem-like CD8^+^ T cell memory differentiation following KDACi.mem exposure. Many metabolic pathways change on T cell activation^41^ we sought to test a causal role for enhanced glutamine breakdown. Selective blockade of glutaminolysis using the GLUD1 inhibitor R162 (but not more proximal inhibitors, SupplementaryFig. 3A-D) reversed valproate-mediated induction of an MPEC phenotype (Fig. 2H). Bypassing glutaminolysis through direct provision of the cell-permeable αKG analogue di-methyl ketoglutarate (DMKG, which mediates anaplerosis directly) reproduced enhanced MPEC differentiation (Figure S3A), although we did not observe differential expression of glutaminolysis enzymes per se (Figure S3E). Our observation that only distal inhibition of glutaminolysis (through GLUD1 by R162) impacted on the memory phenotype indicates that anaplerosis was the likely mechanism responsible. Glutaminolysis may alter cellular function by contributing to reactive oxygen species production, biosynthetic pathways and control of cellular amino acid pools^42,43^ in addition to anaplerosis. Inhibition of more proximal steps in the pathway impact multiple processes, whereas distal inhibition specifically inhibits anaplerosis (Figure S3A).

Note that our data are consistent with previous reports of distinct effects of glutaminolysis inhibition when effected at different points of the pathway^44^. Glutamine restriction can also block cellular (including T cell) proliferation, activation and cytokine production. Blockade at different levels or to different degrees (knockout versus suppression) may also produce different effects^44–47^, including in T cells^48–50^. It is therefore important to interpret studies blocking glutamine uptake with care as these may impact on proliferation or activity which in turn impacts directly on memory differentiation. Our study infers the impact of repurposed drugs that enhance glutaminolysis with no impact on activation or proliferation.

We next sought to determine whether KDACi-induced glutaminolysis had a functional impact on T cell ‘metabolic switching’–a relative increase in oxidative over glycolytic metabolism on activation that is a critical determinant of memory differentiation^51^. Isolated primary CD8^+^ T cells were stimulated and treated for 2 days with either sodium valproate or vehicle before measurement of oxygen consumption rate (OCR) and extracellular acidification rate (ECAR) alongside flow cytometric assessment of the MPEC phenotype. Comparative flux estimates showed that valproate enhanced oxidative metabolism and reduced the glycolytic rate (Fig. 2H, I) without changing cell activation, proliferation or glucose uptake (Figure S3F, G). Consistent with a causal role for enhanced glutaminolysis, this enhanced metabolic switching could be reversed by the addition of R162 (Fig. 2I, J).

These data suggest a dominant role for the anaplerotic effect of glutaminolysis in driving an IL7R^hi^PD1^lo^ MPEC phenotype, with acetylomic and metabolomic changes consistent with enhanced Myc and TCA signalling.

### KDACi drives transcriptomic and epigenomic changes consistent with memory precursor differentiation

**Fig. 3:**
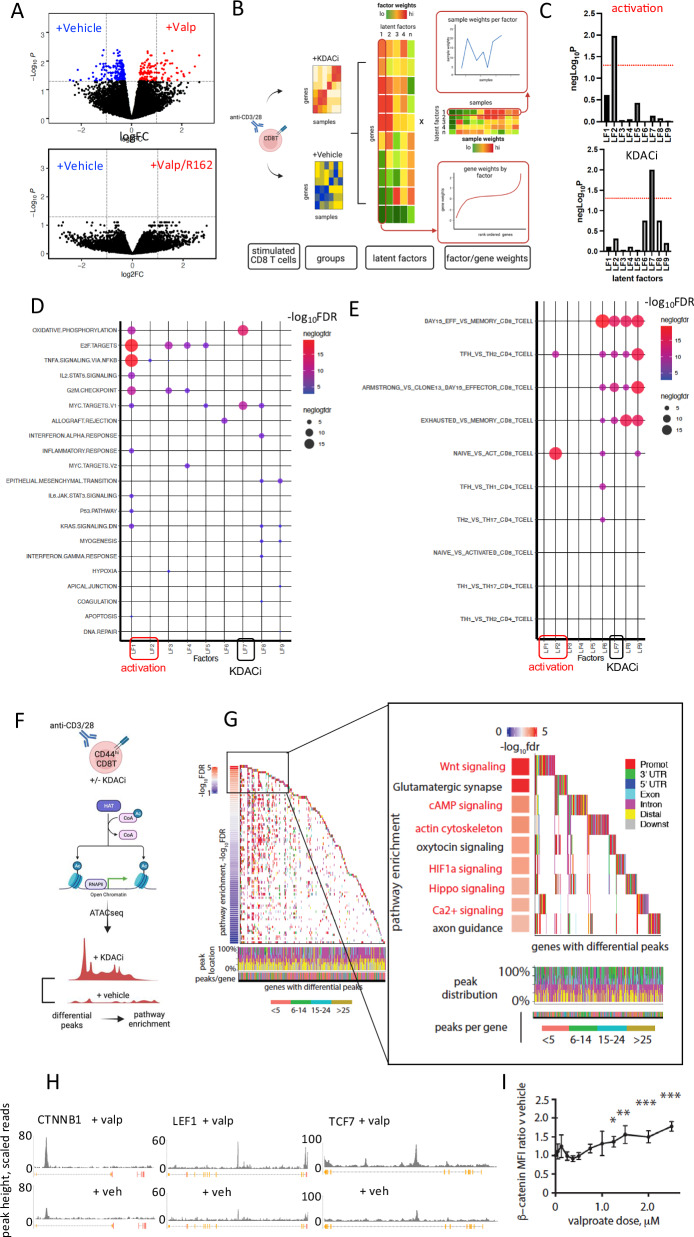
Transcriptomic and epigenomic analysis identifies changes consistent with memory differentiation. **A** Volcano plots showing *n* = 371 differentially expressed genes (DEG:red/blue, FDR < 0.05) induced by treatment of stimulated primary CD8^+^T cells (*n* = 8) for 6 d with valproate (125μM, top) or valproate+25μM R162 (bottom). **B** Schematic of a matrix factorisation approach to identification (factor weights) and interpretation (gene weights) of transcriptional changes (RNAseq) in stimulated, KDACi-treated CD8^+^ T cells. created in BioRender. McKinney, E. https://BioRender.com/wj7 (**C**) Barplot showing association of each of 9 latent factors describing maximal variance in valproate/vehicle treated CD8^+^ T cells with CD8^+^ activation (upper) and treatment group (lower, valproate v valp+R162). **D**, **E** Bubble plots showing pathway enrichment for (**D**) hallmark pathways and (**E**) T cell differentiation signatures (SuppData6) overlapping (*n* > =5 genes) with ranked feature weights defining each of 9 latent factors from (**A**, **C**). **F** Schematic illustration of ATACseq peak set enrichment analysis, created in BioRender. McKinney, E. https://BioRender.com/e5up9ym (**G**) Peak Set Enrichment waterfall plot (upper) showing all overlapping and (inset) the top enriched pathways (*y*-axis heatmap, -log_10_FDR) amongst 3320 ranked genes (*x*-axis) with differential ATACseq peaks on valproate treatment of primary, stimulated CD8^+^ T cells (*n* = 6 per group). (middle/lower) Heatmap to the left indicates significance of enrichment per pathway. Waterfall shows individual genes within each pathway, coloured by location of the most differential peak alongside location of all differential peaks within each gene (peak distribution) and the number of differential peaks per gene (bottom). **H** Representative track plots illustrating differential chromatin peaks in Wnt pathway genes beta catenin (CTNNB1), Lymphoid enhancer binding factor 1 (LEF1) and Transcription factor 7 (TCF7). **I** Line and scatterplot showing beta-catenin induction in stimulated CD8^+^ Tcells (*n* = 6) with increasing dose of sodium valproate (*x*-axis). **J** Schematic illustration of MPEC (IL7R^hi^CFSE^lo^) and SLEC (IL7R^lo^CFSE^lo^) in vitro restimulation. **K**, **L** Representative (**K**) and summary (**L**) scatterplots (mean + /-sem) showing MPEC phenotype (IL7R^hi^CFSE^lo^) on secondary restimulation (without drug treatment) by treatment (apicidin, *n* = 5, valproate, *n* = 5, vehicle *n* = 5) and MPEC phenotype on primary stimulation. Mann Whitney test *P* value index:*<0.05, **<0.01, ***<0.001.

We next sought to test whether KDACi exposure induced global transcriptional changes associated with T cell memory differentiation–the transcriptional signature used for initial compound screening. RNAseq analysis of in vitro stimulated and valproate-treated CD8^+^ T cells revealed *n* = 371 differentially expressed genes (Fig. 3A) that could be reversed with concurrent inhibition of glutaminolysis (Fig. 3B).

To interpret these gene expression changes, we used a machine learning approach (matrix factorisation, MOFA^+^^52^) using variational inference to reconstruct a low-dimensional representation of treated T cell transcriptomes. This method learns a set of latent factors describing maximal variance across the data (Figure S4), with the number of factors present determined by the variance explained. To identify relevant factors, per-sample factor weights can be related to experimental metadata such as treatment group. In addition, while all genes contribute to defining all factors, a sparsity constraint on feature weights^52^ allows identification of the most important features defining each factor (Fig. 3A). In this way, it is possible to interpret a factor’s biological relevance through iterative pathway enrichment analysis. Our analysis learned 9 latent factors (explaining >1% variance) with one (LF7) showing strong, specific association with valproate treatment (Fig. 3C, FDR < 1%). Interrogation of LF7 gene weights identified T cell memory and exhaustion signatures but not those of T cell activation or differentiation (Fig. 3D, E)^53^. Variable T cell activation was instead captured by the orthologous LF2 which reflected inter-individual differences not associated with response to valproate treatment (Fig. 3C–E, and Figure S4A, B). This confirms that valproate treatment in vitro specifically induces transcriptional changes characteristic of polarised memory and exhausted T cells. Analysis^54^ identified two (out of 50) consensus pathways (FDR < 5%), reflecting oxidative phosphorylation and Myc signalling (Fig. 3D) consistent with earlier metabolomic and acetylomic analyses (Fig. 2). These data indicate that KDACi treatment of activated CD8^+^ T cells induces glutaminolysis-dependent transcriptional changes of memory differentiation, similar to those used for initial screening.

### Induced MPEC differentiation is associated with epigenetic modification of Wnt signalling

The generation of durable cellular memory requires epigenetic modification, as memory cells are functionally altered despite remaining genetically identical to naïve precursors^55^. We therefore used ATACseq analysis to identify differential peaks in 3220 genes overlapping with 92 pathways (Fig. 3F, H) induced on valproate treatment of stimulated primary CD8^+^ T cells. Peak Set Enrichment analysis of differentially open genes highlighted the Wnt/Β-catenin pathway (Fig. 3G), with 6 of the top 9 pathways also having established roles in T cell memory formation including HIF1α −^56^, Hippo^57^ and glutamate signalling (Fig. 3G). Wnt/B-catenin signalling is known to arrest effector differentiation and promote both T cell memory formation^58–60^ and glutaminolysis-driven metabolic switching^41^, the latter under the control of Myc^41,61^. Valproate treatment resulted in expansion of peaks in both promoter and regulatory 3’UTR regions in key Wnt pathway genes including CTNNB1, LEF1 and TCF7^59^ (Fig. 3H). Beta catenin (CTNNB1) degradation, known to be prevented by active Wnt signalling^62^, was also inhibited in a dose-dependent manner on valproate treatment of stimulated CD8^+^ T cells in vitro (Fig. 3I) with the phenotype preserved through subsequent rounds of stimulation (Figure S4C–E). Transcription factor (TF) footprint analysis predicted TF binding to differential peaks (Figure S4F). Top TFs were specifically enriched for those observed in primary CD8^+^T cell differentiation (in the Epigenomics Roadmap dataset, Figure S4G, H), including CTCF, known to control heightened secondary responsiveness of CD8^+^T cell memory^63^.

These data are consistent with a model in which a KDACi-induced memory phenotype results from enhanced Wnt, Myc and glutaminolysis pathway activity with epigenomic modification of Wnt pathway genes.

### KDACi enhances memory responses on infection and immunisation in vivo

**Fig. 4:**
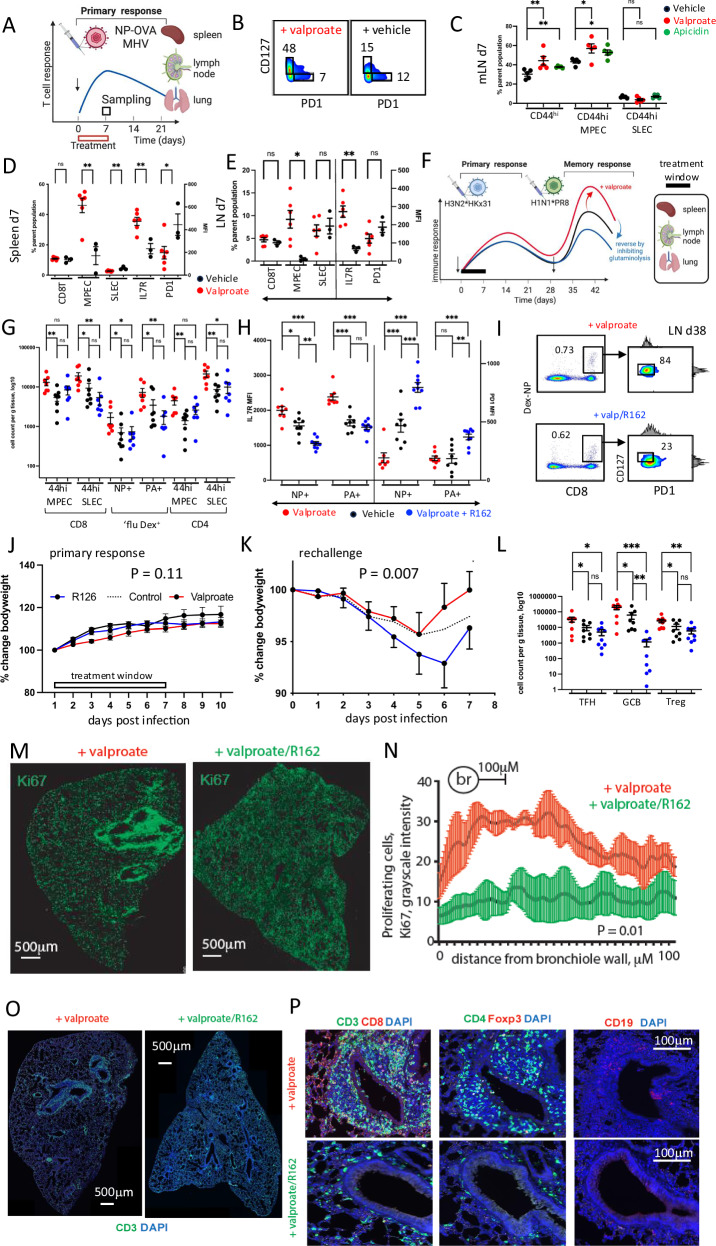
Modulation of in vivo T cell memory responses on infection and immunisation. **A** Schematic of primary response model. MHV = γmurine herpesvirus, created in BioRender. McKinney, E. (https://BioRender.com/3v2ku8x (**B**, **C**) Representative (**B**) and summary (**C**) scatterplots showing MPEC differentiation (%CD44^+^CD8^+^) in mLN d9 post intransal γMHV infection in KDACi (valproate/apicidin) or vehicle treated mice (mean +/- sem, *n *= 5 per group). **D**, **E** Summary scatterplots (mean + /-sem of *n* = 3–6 replicates) showing %MPEC (IL7R^hi^KLRG1^lo^) and SLEC (IL7R^lo^KLRG1^hi^) and IL7R/PD1 expression (MFI) amongst CD44^hi^ CD3^+^CD8^+^ splenocytes d7 post Nitrophenylacetyl[NP]-OVA immunisation with valproate (red) or vehicle (black) treatment for 7 days. **F** Schematic of rechallenge model, created in BioRender. McKinney, E. https://BioRender.com/mzrrxb6. **G**, **H** T cell memory counts (mean + /-sem, *n* = 7–9 per group) (**G**) and influenza antigen-specific phenotype (**H**) in draining mediastinal LN (LN, normalised per g/tissue) d7 post influenza rechallenge following valproate (V, red), vehicle (black) or combined valproate/R162 (blue) treatment during primary infection. *n* = 6–8 biological replicates per group. **I** Memory phenotype (right) of influenza specific (DexNP^+^) CD8^+^ T cells (left) in mLN d7 after rechallenge following valproate (V, red) or valproate/R162 (blue) treatment during primary infection. **J**, **K** Change in bodyweight (% baseline) during primary infection (**J**) and rechallenge (**K**); *P* = 2 way ANOVA, mean + /-sem, *n* = 6–9 replicates per group. **L** CD4 and B cell counts in draining mLN (per g/tissue) d7 post rechallenge following valproate (V, red), vehicle (black) or valproate/R162 (blue) treatment during primary infection. mean + /-sem, *n* = 7–9 replicates per group. **M**–**P** Representative immunofluorescence images (**M**, **O**, **P**) and scatterplot (N) showing peri-bronchial distribution of proliferating cells (**M**, **O**, **P**) d7 after rechallenge following valproate (red) or combined valproate/R162 treatment during primary infection. **N** br bronchiole, 2-way ANOVA *P *= 0.01, mean +/-sem of Ki67 fluorescence assessed in *n* = 16 100μM segments in each of *n* = 3 replicates. mLN = mediastinal lymph node. TFH – T-follicular helper, GCB = GL7^+^ germinal centre B cell. NP influenza nucleoprotein, PA influenza PA protein. 2 sided Mann-Whitney test unless otherwise stated, ns = *P* > 0.05, * = *P* < 0.05, **<0.01, ***<0.001.

Although in vitro phenotypic or epigenomic markers can be used to reliably identify cells with memory-like characteristics^64,65^, durable persistence of cells with functionally altered secondary responses are defining features of memory that can only be validated in vivo^66^. We next tested whether low dose valproate treatment could enhance immune memory using four murine models of infection or immunisation. Low dose KDACi treatment during primary infection (γMHV, Fig. 4A-C, Figure S5A) or immunisation (Nitrophenylacetyl(NP)-OVA, Fig. 4A, D, E, Figure S5B, C) increased the proportion of early (d7pi) MPEC differentiation. We next used a rechallenge model (Fig. 4F) in which primary H3N2 influenza (day 0, A/HKx31) is followed by rechallenge with a heterotypic recombinant H1N1 strain (A/PR8/34) after 30 days. The secondary PR8 strain shares all internal proteins with HKx31 but surface proteins are distinct, allowing detection of secondary T cell responses without the confounding effects of antibody mediated viral clearance^67^. Secondary influenza responses are characterised by expansion of antigen-specific T cells in mediastinal lymph nodes (mLN) followed by migration to the lung^68^ with severe infection characterised by diffuse lymphocytic infiltration^69^ and virus-induced lung immunopathology^70^. Following virus rechallenge (over 30 days after exposure to drug) we observed a significantly greater antigen-experienced (CD44^hi^) and influenza-protein specific (NP and PA) T cell response in secondary mLN of valproate-treated mice (Fig. 4G) but not in spleen or lung (Figure S6). Both MPEC and SLEC cells were expanded consistent with a contribution of anaplerotic metabolism to both and with SLEC generation from memory precursors during the secondary response. As in the in vitro model, influenza-specific T cells showed increased IL7R and reduced PD1 expression (Fig. 4H, I). Increased numbers and proportions of influenza specific T cells were observed in lung and spleen prior to rechallenge (d30pi, Figure S5D–G) which could reflect an expansion of resident memory post-immunisation, although this may also include an increase from circulating cells^71^. Drug treatment had no effect on mice during the primary infection (Fig. 4J) although, on rechallenge, mice previously exposed to valproate had milder disease and recovered faster (Fig. 4K). Expanded CD4 T cell and germinal centre B cell responses were also seen in mLN (Fig. 4L, and Figure S5). In addition to increased numbers, valproate treatment lead to marked clustering of proliferating (Ki67^+^) cells characterised by focal infiltration of CD8^+^T cells, CD4^+^Tcells and CD19^+^Bcells around the peribronchiolar site of influenza infection (Fig. 4M, N) compared to more diffuse parenchymal cell infiltrates characteristic of immunopathology^72^ seen in controls.

Combining valproate treatment with concurrent inhibition of glutaminolysis in vivo partially reversed the valproate-induced expansion of memory responses (Fig. 4G–P, and Figure S5). This demonstrates that, while the effect may be mediated through more than one cell type, the in vivo effect is glutaminolysis-dependent.

**Fig. 5:**
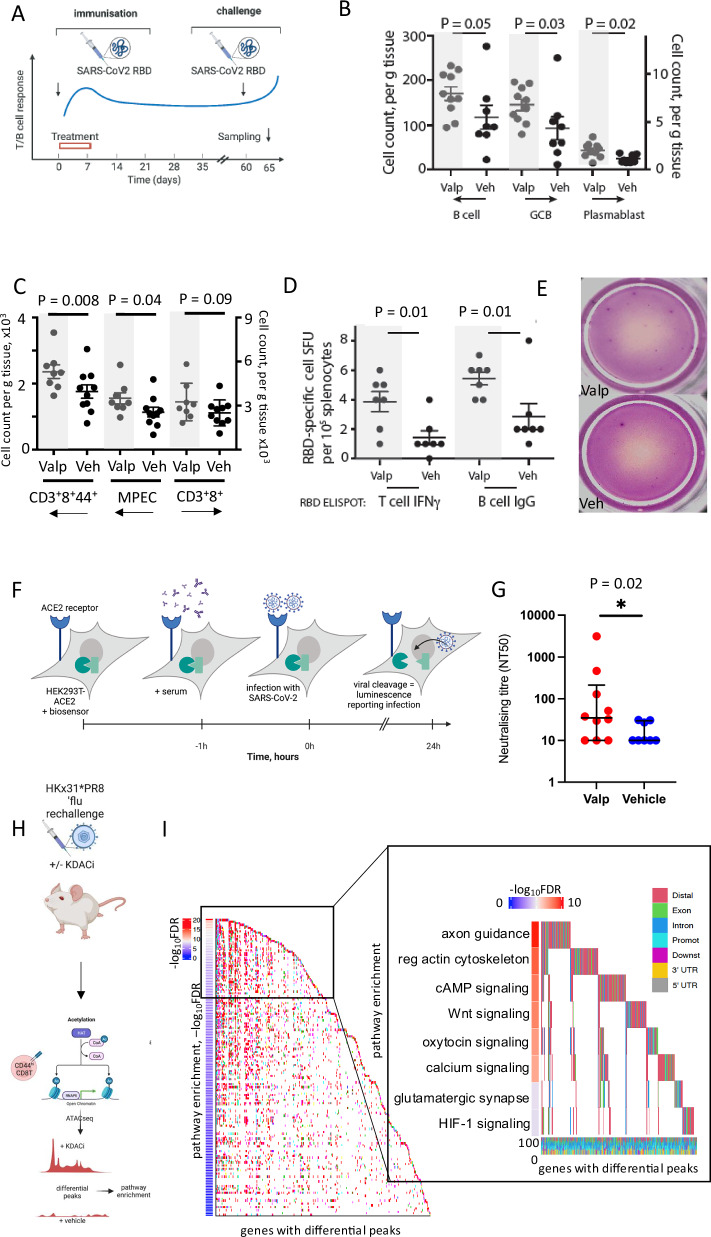
Modulation of in vivo cellular and humoral memory responses. **A** Schematic of SARS-CoV-2 receptor binding domain (RBD) immunisation regimen with valproate treatment or vehicle d0-7, *created in BioRender. McKinney, E.*https://BioRender.com/dlkrmy2 (B, C) Summary scatterplots (mean + /-sem, *n *= 8–10 per group) showing splenic B and T cell populations d5 post RBD rechallenge; arrows indicate relevant axis (**D**, **E**) Summary scatterplot and representative plates (**E**) showing RBD-specific B and T cell ELISpot titres on d5 post RBD rechallenge by treatment regimen (valproate or vehicle). **F** Schematic of viral protease-activatable biosensor model of SARS-CoV-2 infectivity, created in BioRender. McKinney, E. https://BioRender.com/bl67c3u (**G**) Scatterplot (median + /- IQR) showing SARS-CoV-2 neutralising antibody titre (NT50) by treatment group (valproate/vehicle). **H** Schematic illustration of ATACseq investigation of CD3^+^8^+^CD44^hi^ T cells following heterologous influenza rechallenge with valproate (KDACi) treatment, created in BioRender. McKinney, E. https://BioRender.com/e5up9ym (**I**) Waterfall plots showing all overlapping and (inset) the top enriched pathways (*y*-axis heatmap, -log_10_FDR) amongst 3632 ranked genes (x-axis) in which differential ATACseq peaks were identified in CD8^+^CD44^+^ cells isolated from spleens of mice (*n* = 6) on d5 post rechallenge with heterotypic influenza (Fig. 4) after early treatment (d0-d7) with either valproate or vehicle (Fig. 4C). Location of differential peaks within genes (% by region) indicated in lower bar of inset plot. 2 sided Mann-Whitney test: ns = *P *> 0.05, * = *P* < 0.05, **<0.01, ***<0.001.

We next tested the impact of valproate treatment on both cellular and humoral responses in a murine model of SARS-CoV2 immunisation (RBD, Fig. 5A). Valproate-treated animals showed expansion of germinal centres, plasmablast responses (Fig. 5B), RBD-specific memory T cell populations (Fig. 5C) alongside a > 2-fold expansion in the magnitude of RBD-specific B and T cell memory responses following peptide rechallenge (Fig. 5D, E). Using a live SARS-CoV-2 reporter cell assay (Fig. 5F)^73^, we observed a significant increase in viral neutralisation compared to vehicle-treated and immunised controls (Fig. 4G). These data show that low-dose valproate treatment during priming results in a functionally improved secondary response on rechallenge with expansion of both cellular and humoral responses.

To explore the epigenetic modification of T cells activated in vivo we undertook ATACseq analysis of CD44^+^CD8^+^ T cells from spleens of valproate or vehicle-treated animals on day 5 following heterotypic influenza rechallenge (Fig. 5H). Amongst differential peaks in 3632 genes, we observed differentially open chromatin in pathways similar to those of valproate-treated primary human CD8^+^ T cells in vitro (Fig. 3G), including similar increases in chromatin accessibility of Wnt, HIF and glutamatergic signalling pathways (Fig. 5I).

Together these data show that valproate treatment during in vivo priming promotes expanded and functionally superior cellular and humoral responses on secondary rechallenge in multiple models of infection and immunisation.

### Human vaccine responses are boosted by a repurposed immunometabolic adjuvant

**Fig. 6:**
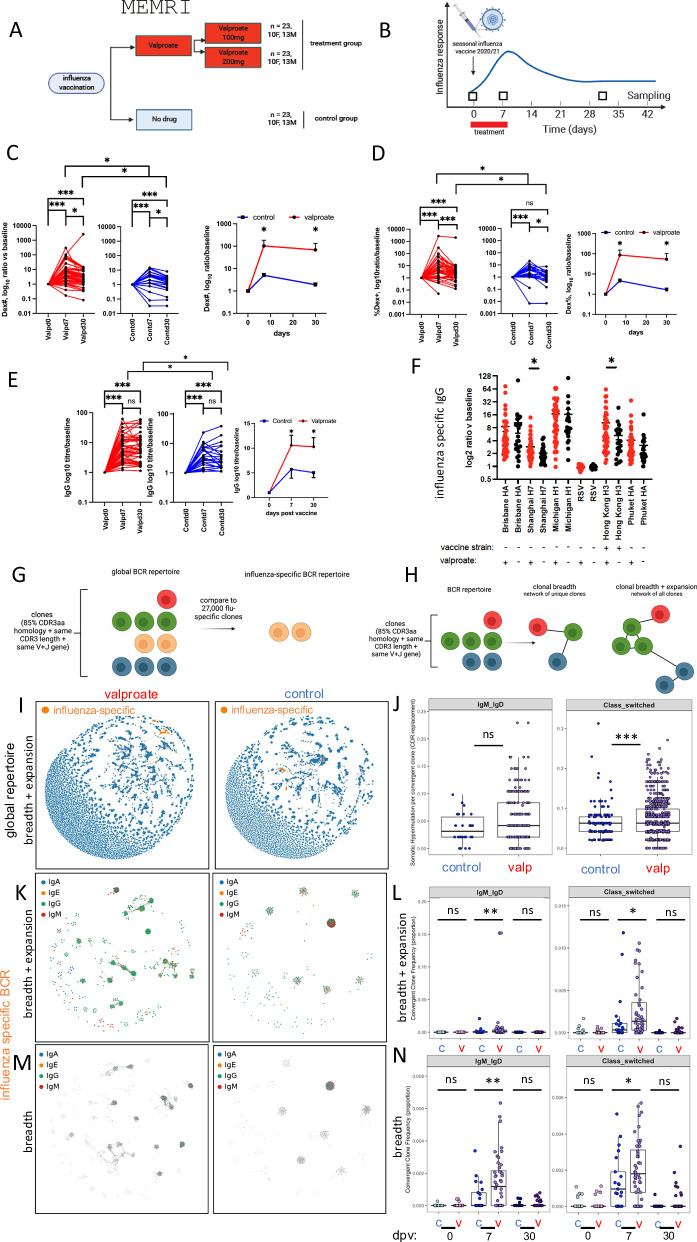
Boosting human vaccine responses with a repurposed immunometabolic adjuvant. **A**, **B** Schematic of the MEMRI experimental medicine study design and treatment/sampling regimen, created in BioRender. McKinney, E. https://BioRender.com/g7mzu9f (**C**–**E**) Scatter/line plots showing paired individual (left) and summary (right) baseline-normalised log_10_ ratios (mean +/- sem) of influenza-specific CD8^+^ T cell counts (**C**), frequencies (**D**) and influenza vaccine-specific IgG titre (**E**) in volunteers following vaccination with (*n* = 46) or without (*n* = 23) valproate treatment from d1-7 post vaccination. **F** Summary scatterplot showing influenza vaccine strain-specific log_2_ IgG titres (baseline-normalised ratio) on day 7 post-vaccination following valproate (red) or no treatment (black).*n* = 69, mean + /-sem (**G**, **H**). Schematic illustrations showing definition of influenza-specific ‘convergent’ clones (**G**) and definition of repertoire diversity networks (**H**) measuring unique (breadth) or all (breadth+expansion) convergent clones. Representative dandelion plots (I, K, M) and summary scatterplots (**J**, **L**, **N**, *n* = 69) illustrating BCR diversity metrics by treatment group. Linked convergent clones represent VDJ sequences with >85% amino acid homology, equal CDR3 length and V/J gene use. Boxplots showing q25, median, q75, whiskers showing 10^th^/90^th^ centiles. (**I**) Representative dandelion plot showing global BCR repertoire network of all clones (breadth+expansion). Orange clones indicate influenza specific BCR. **J** Scatterplot illustrating clonal somatic hypermutation (SHM) rates in naïve (left) and switched memory populations (right) in vaccinated and valproate treated or untreated (control) individuals in the MEMRI study. Representative dandelion plots (**K**) showing influenza specific BCR clonal network (all clones/breadth + expansion) from valproate treated (left) and control (right) individuals d7 post vaccination. **L** Summary scatter and boxplots (mean/IQR + /−95%ci) illustrating clonal frequency of all influenza-specific clones (breadth + expansion) in control (C) or valproate (V) treated individuals by sampling timepoint. **M** Representative dandelion plots showing influenza specific BCR clonal network (unique clones/breadth) from a valproate treated (left) and control (right) individual. (**N**) Summary scatter and boxplots (mean/IQR + /−95%ci) illustrating clonal frequency of unique influenza-specific clones (breadth) in control (C) or valproate (V) treated individuals by sampling timepoint (d0, d7 or d30 post vaccination). 2 sided Mann-Whitney test or Wilcoxon signed rank test (where comparing to baseline ratio =1), ns = *P* > 0.05, * = *P* < 0.05, **<0.01, ***<0.001. dpv days post vaccination.

The magnitude of both the heterotypic cellular memory T cell response^3^ and the antibody response^74^ to influenza vaccination is associated with protection from subsequent infection, establishing them as useful immunological correlates of protection against influenza infection^75^. We therefore undertook an experimental medicine study (the Modulating Emerging Memory Responses to Immunisation, MEMRI study) to determine the impact of randomised low dose sodium valproate treatment on correlates of protection following seasonal influenza vaccination (Fig. 6A, B, Figure S7A). Genotyped (HLA-A*0201^+^) healthy volunteers underwent stratified randomisation (protocol, Methods) to receive either low dose sodium valproate (equivalent to 5-10% of its approved dose as an anti-convulsant) or no additional treatment daily for seven days starting on the day of seasonal influenza vaccination (Fig. 6A). No difference in adverse events was seen (Figure S7B), although valproate treatment drove significant increases in both cellular and humoral responses to influenza (normalised to baseline to account for prior influenza exposure, Fig. 6C-F) with no change in response to an irrelevant antigen (RSV, Figure S8). Treatment resulted in an average >10-fold expansion with maintenance of both the absolute number (Fig. 6C) and frequency (Fig. 6D) of ‘flu-specific CD8^+^ T cells beyond 30 days despite control levels falling to baseline. A > 2-fold expansion and similar maintenance of vaccine strain-specific influenza IgG and IgA titre (Influenza A/Hong Kong H3, Fig. 6E, F, and Figure S8) was also observed.

Increased titres to influenza serotypes not included in the seasonal vaccine (Shanghai H7, Fig. 6F, Figure S8) implied an adjuvant-driven increase in the breadth as well as the magnitude of response. This is supported by increased TFH and germinal centre B cell responses in the murine influenza (Fig. 4L) and RBD immunisation models (Fig. 5B). To test this, we analysed changes in the global BCR repertoire, finding an expected increase in somatic hypermutation and a decrease in diversity after vaccination (Figure S9) and with no effect of treatment on the global BCR repertoire (Fig. 6I, Figure S9). However, on restricting analysis to 5274 influenza-specific sequence clusters (by integrating with 27,000 sequences of known specificity), we observed higher rates of somatic hypermutation (Fig. 6J), clonal expansion (total clonal frequency, Fig. 6K, L) and clonal breadth (unique clonal frequency, Fig. 6M, N) in valproate treated individuals.

Together these data show that use of a repurposed KDACi (sodium valproate) can significantly boost the magnitude, breadth and maintenance of influenza vaccine responses in humans.

The data are derived from a human experimental medicine study and quantify impact on validated correlates of protection rather than on clinical efficacy directly. However, given the effect size seen, it is very likely that the enhanced response would be clinically relevant^3,74^.

## Discussion

In this study we describe the identification and mechanism of a repurposed immunometabolic adjuvant promoting both cellular and antibody responses in mice and humans.

Our systems immunology approach demonstrates that systematic identification of repurposable drugs is feasible, can be translated quickly into human experimental studies and can concurrently be used to support mechanistic discovery. Our initial screen matched T cell signatures against drug signatures primarily generated in transformed cell lines. It is therefore inevitable that pathways conserved across cell types will be more easily identifiable. The causal mechanism identified–enhanced glutaminolysis–is conserved in many cell types, and it is clear that treatment modulated CD4 T cell and B cell responses in vivo in addition to those in the CD8 T cells used for screening (Fig. 4L, Figure S5). This is consistent with a likely effect of valproate treatment on other cell types (an effect we had also observed in vitro, Figure S1I). Thus, although we set out to modulate T cell memory differentiation, B cell responses were also affected (although with no clear impact on myeloid phenotypes). This likely reflects a role for glutaminolysis facilitating proliferation and survival in other cell types^43,76–78^, although we cannot exclude (and it is challenging to confirm) a possible impact on T cells with secondary modulation of B cell and antibody responses. Our human studies also could not differentiate whether the observed expansion in T cells reflects expanded resident (Trm) or circulating (Tcm/Tem) memory populations, or both. We also observed greatest responses from purified naïve cells in vitro, although with Tem cells also showing increased responses (Fig. 1G). Two pieces of evidence suggest that both naïve and recall responses were impacted by treatment in the human study. First, VDJ sequence repertoire analysis after vaccination demonstrated treatment-driven increases in both breadth and expansion of the influenza-specific B cell response (Fig. 6G-N). This was observed in both naïve (IgM/IgD) and memory (class-switched) sequences (Fig. 6L, N), confirming that both are impacted by treatment. Second, boosting of IgG titres was observed for both vaccine-included (Fig. 6E) and other influenza serotypes (Fig. 6F, Supplementary Fig. 8). This suggests expansion of pre-existing memory responses, although cross-reactivity with vaccine-included strains cannot be excluded. For clinical impact on vaccine responses, boosting pre-existing memory has greater functional relevance than enhanced priming of naïve responses.

Glutaminolysis is essential for T cell activation^79^. Activated cells replenish TCA cycle metabolites from amino acids, principally glutamine, in a process termed anaplerosis to maintain mitochondrial function^43^. Glutamine metabolism contributes to the maintenance of pluripotency in both cancer^80^ and embryonic stem cells^81,82^ through multiple mechanisms including availability of TCA cycle intermediates and epigenetic modification. Memory T cells share functional properties with stem cells, including self-renewal and pluripotency^83^. Our data suggest that enhanced survival of activated T cells into a memory precursor pool is an analogous process and depends on overlapping metabolic pathways.

Both the acetylomic and transcriptomic analyses identified altered Myc signalling (Fig. 3) with induced memory differentiation. Myc is known to be a master regulator of mitochondrial metabolism in activated T cells^35^ and controls induction of glutaminolysis^61,84^. Our data show that lysine deacetylation of specific proteins can promote memory differentiation and that this process is dependent on enhanced glutaminolysis. However, our data cannot differentiate between a direct induction of Myc signalling (with subsequent induction of glutamine breakdown), a direct modification of proteins involved in glutaminolysis, or both.

Memory T cell differentiation is associated with, and reliant upon, epigenetic modification^85^. Consistent with this, our systems analysis identified epigenetic modification of memory-associated pathways in KDACi-treated cells and mice including Wnt^59^, HIF1α−^56^ and Hippo^57^ in addition to glutamate signalling. While each of these pathways has been linked to memory T cell differentiation (and the involvement of multiple parallel pathways is likely), Wnt showed the strongest enrichment. Wnt signalling is known to directly regulate both memory T cell differentiation^59^ and also Myc signalling^86^, although is best studied in glutamine-dependent cancer cells. Our data are therefore consistent with a model in which altered lysine acetylation results in enhanced Myc signalling and glutaminolysis promoting a self-renewing memory phenotype with concurrent epigenetic modification of Wnt and other pathways to similarly allow persistence of that phenotype through subsequent rounds of reactivation.

Our data have broad clinical relevance in multiple contexts, indicating the potential application of low dose KDACi as an immunometabolic adjuvant for vaccination against infection or to promote a durable phenotype of adoptive cell therapy in cancer. With demonstrated in vivo efficacy at low dose, selected KDACi could be rapidly translated to boost immune memory responses to both infection and vaccination, including to influenza and SARS-CoV2 vaccines. Such enhanced memory may be of particular benefit in the elderly, where vaccine responses are suboptimal, or to limit the ability of new viral variants to evade vaccine-induced serological protection through promoting increased breadth of the induced response. We show an average 3 and 10 fold increase in the magnitude of vaccine-specific antibody and cellular responses respectively, both correlates of protection after seasonal influenza vaccination in humans. However, further clinical studies will be necessary to quantify direct protection from infection.

## Methods

This research complies with all ethical regulations and was approved by the HRA and Health and Care Research Wales (IRAS id 287814).

### Experimental model and study participant details

#### In silico screen

Target CD8^+^ T cell memory signatures defining CD8 SLEC v MPEC (ImmuneSigDB M3027, M3028), CD8+ exhaustion v memory (GSE41867, ImmuneSigDB, M9480,9482), CD2 memory (E-MTAB-3470) were obtained from MSigDB or GEO as indicated in Supplementary Data1. Signatures were screened against characteristic direction drug response signature libraries curated from GEO (Drug_perturbations_from_GEO_2014), LINCS (L100 chem perturbation) and the connectivity map (CMAP) using the EnrichR platform^19^. Compounds with significant overlap (overlap >=5 and adjusted P < 0.05) were considered for inclusion for in vitro phenotypic screening. Combined enrichment scores are computed as modified Fisher’s exact test multiplied by deviation from an expected rank value^19^.

### in vitro phenotypic screen

Primary human CD8^+^ T cells were separated from leucocyte cones obtained from NHS Blood and Transplant (Addenbrooke’s Hospital, Cambridge, UK) by centrifugation over ficoll and positive selection using magnetic beads as previously described^87^. The purity of separated cell subsets was determined by three-colour flow cytometry. Purified T cells (>95%) were labelled with 2.5 µM CFSE (Invitrogen) and resuspended in complete RPMI 1640 (Sigma Aldrich) in the presence of 10% FCS and then stimulated in sterile, U-bottomed 96 well culture plates (Greiner) using MACS iBead particles (1:2 bead:cell ratio, Miltenyi) conjugated to anti-CD2/CD3/CD28 in the presence of non-limiting IL2 (10 ng/ml, Gibco Life technologies) for 6 days. Selected compounds were added at the time of stimulation (d0), or 2 (d2) or 4 (d4) days after as indicated. T cell memory subsets were isolated using flow cytometric sorting (FACSAriaIII cell sorter, BD Biosciences) following MACS enrichment of CD8^+^ T cells as above, into naïve (Tn, CD45RA^+^CD62L^+^), effector memory (Tem, CD45RA^-^CD62L^-^), central memory (CD45RA^-^CD62L^+^) and Temra (CD45RA^+^CD62L^+^) populations before co-culture with drugs as indicated. T cells were stimulated in the presence of treatments as indicated (Supplementary Data 2) for 6 days before undergoing high-throughput immunophenotypic screening (BD LSR Fortessa HTS) with quantification of IL7R. Reactions were standardised with multicolour calibration particles (BD Biosciences) with saturating concentrations of the following antibodies: IL7R (BD Biosciences, Clone HIL-7R-M21), CD25 (Clone M-A251), live/dead discrimination (AquaFluorescent amine-reactive dye, Invitrogen). Data was analysed using FlowJo software (Tree Star), extracting immunophenotypic traits (IL7R MFI, division (CFSE^dil^), CD25mfi, % live cells) with ratios calculated against pooled vehicle treated controls in triplicate.

### Compound selection and titration

Compounds were selected from the in silico screen on the basis of consistent enrichment against multiple signatures taking into consideration likely impact on cellular viability, toxicity and availability (Supplementary Data 4, 5). Additional ligands and cytokines with a demonstrated association with T cell memory formation were also included (Supplementary Data 2) with Fc-Chimaeric ligands hybridised to individual wells of a 96-well U-bottomed culture plate (Greiner) for 2 h at 37 °C before washing in sterile complete RPMI-1640 and addition of cell suspensions for stimulation. Dose ranges for inclusion were selected on the basis of demonstrated in vitro IC_50_ for target effects with 3 doses included per treatment (Supplementary Data 2). Effects were considered by dose and also by peak effect per treatment with each value compared to the median of triplicate repeat vehicle stimulation. In vitro KDACi efficacy was confirmed by measuring impact on total cellular protein acetylation by flow cytometry (acetylated lysine multiMab mix, CST) and for selected compounds (apicidin and sodium valproate) taken into genomic, functional and in vivo assays standard concentrations were generated using targeted LC-MS/MS quantitation (PK/B Core facility, CRUK, Cambridge).

### Infection and immunisation models

Mouse experiments were carried out in specific pathogen-free conditions at The Wellcome Trust Sanger Institute. Experiments were performed under project licence P653704A5 with institutional approval by a local Animal Welfare and Ethical Review Board (AWERB) and in accordance with the United Kingdom Animals (Scientific Procedures) Act of 1986. Basic husbandry and animal welfare was provided daily by trained and competent animal technicians and mice were given ad libitum access to food (Safe Diets, Safe 105) and water in individually ventilated cages with relative humidity (RH) of 55 ± 10%, temperature 21 + /−2C and a standard 12 h light/dark cycle. All animals were bred at the Wellcome Sanger Institute and were wild type mus musculus C57BL/6N^WTSI^ and were between 6-8 weeks of age. Female C57BL/6 mice were infected i/n with γMHV virus or immunised with 200μl NP-OVA in alum with spleens harvested d7 (NP-OVA) or d9pi (γMHV). For the heterotypic influenza model C57BL/6 mice were given a primary i/n challenge with 500pfu H3N2 influenza (A/HKx31) and a secondary challenge with 10^5^pfu i/n H1N1 (A/PR8/34) influenza d31 post-primary challenge with tissues (lung, spleen, MLN, blood) harvested on d30pi and d38pi for stable memory and rechallenge assessment respectively. Numbers of mice are as indicated in the Figure Legends.

For each model mice were administered sodium valproate 0.1% w/v in drinking water, sodium valproate plus R162 (90% corn oil, 10% DMSO) 20 mg/kg/day intraperitoneally or matched vehicle control as indicated (Fig. 4). For SARS-CoV-2 immunisation, mice were administered receptor binding domain (RBD) peptide (gift, YM) 50μg s/c in QuilA adjuvant either once (R1) or three times (R2) as indicated. Tail bleeds were taken (d58pi) prior to RBD re-immunisation (d60) with tissues harvested on d65pi.

### The modulating emerging memory responses to immunisation (MEMRI) study

The Modulating Emerging Memory Responses to Immunisation (MEMRI) study was a non-CTIMP interventional experimental medicine study as defined by EU Directive 2001/20/EC and approved by the HRA and Health and Care Research Wales (IRAS id 287814). The study has been verified by a formal MHRA scoping review as an experimental medicine study exploring correlates of protection, therefore not requiring registration as a clinical trial. Participants were identified from the NIHR Cambridge BioResource (study# CBR213) with informed, written consent taken from all participants. Prespecified inclusion and exclusion criteria were: [for inclusion] age >18 years old, able to give informed consent and hetero/homozygous for HLA-A*02:01; [for exclusion] known/suspected primary or acquired immune deficiencies due to a congenital condition, treatment or disease process, history of thymectomy, any use of immune suppressing medications, including glucocorticoids and topical formulations, current or within the previous 60 days, prior vaccination with or contraindication to receiving the 2020/21 seasonal influenza vaccine, any intercurrent acute febrile illness, active or in the previous 90 days and pregnancy, lactation or intention to conceive during the study period in women of childbearing potential. Individuals >65 years of age were precluded by recommendation of the Cambridge BioResource Advisory Board due to the COVID pandemic and requirements for hospital attendance for venepuncture.

Sequential participants (n = 74) were randomised at recruitment into one of three treatment groups (sodium valproate 100 mg od, 100 mg bd or control without treatment) using a biased coin minimisation and marginal balance method (probability 0.7, MinimPy2) specifying age (18-30, 30-45, 46-65 years old) and sex (male or female) as stratification factors. Accurate stratification for selected factors was confirmed on the final cohort (Figure S6). All subjects received a single dose of adjuvanted trivalent seasonal Influenza vaccine 2020/21 adjuvanted with MF59C.1 (Seqirus Ltd) in the Cambridge Clinical Research Centre (CCRC). Vaccines comprised 15ug haemagglutinin from each of A/Hong Kong/267/2019 IVR-208 (H3N2), A/Victoria/2454/2019, IVR-207 (H1N1) and B/Victoria/705/2018 BVR-11 (B). Participants in the treatment group received either 100 or 200 mg per day of sodium valproate (Epilim, Sanofi) orally for 7 days beginning on the day of vaccination. The primary endpoints of the study were the baseline normalised ratios of influenza specific CD8^+^ T cell number and the influenza specific humoral responses (MSD) in the treated (sodium valproate 100 mg od/bd) and control groups. For cellular memory responses a group size of n = 25 was estimated to give 80% power to detect a superiority margin of 30 with group A mean 110 and group B mean 55 with sd 50 and a sampling ratio of 1 at a type 1 error rate of 0.05 with parameters estimated from Sridhar et al. ^3^. For humoral memory responses: A group size of n = 36 results in 80% power to detect a superiority margin of 2 with group A mean 18 and group B mean 32 with sd 32 a sampling ratio of 1 at a type 1 error rate of 0.05 and with parameters estimated from Nakaya et al. ^88^.

### MEMRI sample processing and analysis

20 ml whole blood was obtained on each visit from each participant with PBMC isolated over a density gradient (Histopaque 1077, Sigma) and frozen in aliquots of 1×10^7^ cells in 90%FBS/10% DMSO for batched processing. A 2.5 ml PAXgene RNA tube (Qiagen) was collected and frozen at −70C for later RNA extraction and 2.7 ml EDTA blood was collected with 50ul transferred to a Trucount tube (BD Biosciences) and stained with Live/Dead Aqua (Invitrogen), CD3 (clone OKT3, BioLegend), CD8 (clone SK1, eBiosciences) according to the manufacturer’s instructions. Dextramer quantification was performed by staining aliquots of 1×10^7^ frozen/thawed PBMC with the above antibodies plus influenza-specific dextramer (GILGFVFTL, HLA-A*0201, Immudex). Absolute CD8^+^ T cell counts were calculated using the formula A = X/Y × N/V, where A = absolute CD8^+^ T cell count, X = # CD8^+^ T cell events, Y = number of bead events, N = number of beads per test, V = test volume. The absolute number of influenza-specific CD8 + T cells were then calculated as D = A*F where D = influenza-specific Dextramer #, A = absolute count CD8^+^ T cells, F = influenza-specific Dextramer population as fraction of total CD8^+^ T cell population.

Influenza-specific IgG and IgA titres were quantified on frozen/thawed plasma (1:1000 dilution) using the Respiratory virus panel 1 according to the manufacturer’s instructions (Mesoscale Discovery) incorporating both IgG and IgA responses to ‘Flu A (Hong Kong H3/Michigan H1/Shanghai H7), ‘Flu B (Brisbane HA/Phuket HA) and RSV (Pre-Fusion F) as indicated.

Haemagglutination inhibition (HAI) titres were measured at the WHO Collaborating Centre for Reference and Research on Influenza (VIDRL, Melbourne, Australia) using influenza strains included in the 2020/21 seasonal vaccine. Specifically, these were influenza A (IVR-208: A/Hong Kong/2671/2019; Passage E7/D10, E1, ID BA10043042), influenza A (IVR-207: A/Victoria/2454/2019; Passage E4/D6, ID VI-1649) and influenza B (BVR-11: B/Victoria/705/2018; Passage E4, ID BA10043417). All viruses were used at concentration of 4HA units/25μl with Turkey red blood cells used in all assays.

For both influenza-specific cellular and antibody responses, titres were normalised to pre-vaccination levels and a ratio calculated using the formula: R = T/t, where R = response ratio, T = titre post-vaccination (day 7 or day 30) and t = titre pre-vaccination (day 0). Statistical tests were either one-sided Mann-Whitney tests of significance at each timepoint or a mixed linear model with a natural cubic spline fit with fixed effects as vaccine, timepoint and treatment group and subject id as a random effect. Ethical approval was obtained for up to 180 participants to allow subgroup analysis by drug dose and other covariates (sex, prior response etc).

As recruitment was undertaken during the period of a COVID pandemic, this was slower than anticipated and recruitment of those >65 years old was precluded by our BioResource SAB. Consequently, the study recruited n = 69 individuals, with adequate power to analyse against treatment/control groups but not for subgroup analyses. However, for transparency, responses by dose (Figure S9) and with adjustment for covariates (using multivariate mixed effects logistic regression) are also presented (Figure S8F) using the form:.

model <-glm(treat.group~response + age + BMI + Sex + smoking + AE + (1|id), family=binomial (link=logit)), where BMI = body mass index, smoking = smoking status, AE= adverse events.

A full protocol of the MEMRI study is available at the DOI listed in Supplementary Table 1 and includes full information on study design, power calculations and assumptions, randomisation, blinding and inclusion/exclusion criteria.

### Quantification and statistical analysis

#### Statistics & reproducibility

The data presented in the figures represent a minimum of three independent experiments. The number, statistical test and descriptive metrics used are defined in each corresponding figure and its legend. Statistical analyses were conducted in R and using GraphPad Prism software (La Jolla, CA, USA). A Mann Whitney U test was employed to compare quantitative data between groups and significance was determined at P < 0.05. No data were excluded from the analyses.

### Lysine Acetylation proteomic data

Stable isotope labelling with amino acids in cell culture (SILAC) mass spectroscopic (MS) quantitation of enriched acetylated peptides was obtained from^26^. We extracted SILAC H/L ratios from KDACi-treated HeLa cells, verifying comparability between and then combining data across technical replicates. Median H/L ratios were converted to a pairwise distance matrix using the ape and phylobase packages in R before visualisation as an annotated, unrooted tree using iTOL (https://itol.embl.de/). KDACi compounds were classified on the basis of MPEC phenotype induction (KDACi.mem/eff) and differentially acetylated peptides were determined by comparison between these groups (threshold fdr 5%). Peptides were summarised to protein level and pathway enrichment analysis within a list of differentially acetylated proteins ranked by fold change was undertaken against Hallmark (HM), KEGG and National Cancer Institute Pathway Interaction (NCI-PID) databases respectively as indicated using the BioConductor package fgsea in R. Proteins showing both up and down-regulated acetylation were considered together for enrichment as the effects of acetylation vary by peptide residue and protein context.

### Metabolomic analysis

Raw data was extracted, peak-identified and QC processed using Metabolon’s hardware and software. These systems are built on a web-service platform utilising Microsoft’s.NET technologies. Compounds were identified by comparison to library entries of purified standards or recurrent unknown entities. Metabolon maintains a library based on authenticated standards that contains the retention time/index (RI), mass to charge ratio (*m/z)*, and chromatographic data (including MS/MS spectral data) on all molecules present in the library. Identified metabolites normalised to dsDNA concentration, log transformed and missing values imputed as the lowest value identified per compound. A Mixed model ANOVA with biological replicate as a random effect was used to identify differential compounds between experimental conditions (FDRq<0.1). Pathway enrichment of differential metabolites was undertaken using MetaboAnalyst^89^ and metabolite set enrichment analysis, taking pathways from HMDB^90^ as indicated.

### Seahorse metabolic flux analysis

Measurement of oxygen consumption rate (OCR, XF MitoStress test, Agilent) and extracellular acidification rate (ECAR, XF Glycolysis Stress test, Agilent) was undertaken using the Seahorse XFp energy phenotype system (Agilent) according to the manufacturer’s instructions. Primary human CD8^+^ T cells were isolated, stimulated and cultured as above for 2 days in the presence of either 87.5μM sodium valproate before being transferred to Seahorse-XF base medium (Agilent, Cat No. 103335-100), immobilised onto a Seahorse XF96 cell culture microplate (Agilent) using Cell-Tak (Corning) with concurrent aliquots taken for immunophenotypic assessment including live/dead (AquaFluorescent amine-reactive dye, Invitrogen) and cell proliferation (CFSE dilution) quantification. Raw data was extracted using Wave software (Agilent) and normalised to baseline values with comparative flux estimates obtained from summarised peak values across biological replicates. For real-time cellular metabolic analysis using Agilent Seahorse XF technology normalisation is a standard requirement of data processing to allow interpretation^91,92^. Cell quantity per well is a commonly accepted reference value for data normalisation alongside normalisation to baseline value to show relative change in flux over time between multiple replicate runs.

### RNA-seq analysis

Merged bam file QC was undertaken using the QoRTs package in R with assimilation using multiQC. Merged bam files were read into R using featureCounts (Rsubread) with feature filtration to >cpm1 in >6 samples (n = 16,402) and annotation using BioMart followed by pairwise differential expression, undertaken in primary human CD8^+^ T cells using a contrast matrix with a pairwise design. This was undertaken in limma^93^ using an additive model matrix, considering it as a special case of blocking with block size of n = 2. Matrix factorisation analysis was undertaken using the MOFA package^94^ in R with feature set enrichment performed using the Bioconductor package fgsea in R with gene sets extracted from MSigDB using the msigdbr package as indicated. RNAseq data has been deposited in the European Bioinformatics Institute ArrayExpress repository with accession number listed in the Supplementary Table 1.

### ATAC-seq analysis

Peak calling and differential peak identification undertaken using the esATAC BioConductor package in R. KEGG pathway enrichment and visualisation of treatment-specific differential peaks was undertaken using the BioConductor packages clusterProfiler, GenVisR and rtracklayer in R. ATACseq data has been deposited in the European Bioinformatics Institute ArrayExpress repository with accession numbers available in Supplementary Table 1.

#### BCR Sequence analysis

The BCR sequence data was processed using the Immcantation toolbox (v4.0.0). Using pRESTO, raw reads were filtered according to base quality (median Phred score of ≥30). The unique molecular identifiers were annotated and a consensus sequence constructed. Forward and reverse reads were merged. Primers were trimmed and the constant region annotated^95^.

Immunoglobulin gene use and sequence annotation were performed in IMGT V-QUEST^96^. BCR clones were assigned using the Change-O package using the single-nucleotide Hamming distance model^97^. The Alakazam package was used to analyse the BCR sequencing data for diversity estimation of CDR3 sequences; the diversity estimates were adjusted for sequencing depth by subsampling with multiple iterations^97^. Somatic hypermutation levels (including silent and non-silent mutations) per unique IGHV-D-J region per isotype were calculated over the CDR1/2 and FWR regions for each individual sample using the observedMutation function within the SHazaM package^97^.

#### IGH reference sequences

Influenza specific IGH sequences were collected from the literature^98–101^. Convergent IGH clones were identified based on matching V and J gene and CDR-H3 length with a minimum 85% CDR-H3 amino acid sequence identity. CDR-H3 amino acid sequence clustering was performed using CD-HIT^102^ with options -c 0.85 -l 4 -S 0 -g 1 -b 1. Clusters were identified as influenza specific if they co-clustered with an influenza known sequence.

Statistical tests were performed in R using Wilcoxon tests for significance (non-parametric test of differences between distributions).

### Glucose uptake assay

Primary human CD8^+^ T cells were isolated, stimulated and cultured as above for 6 days after which cells were incubated in glucose-free media for 60 min at 37 C in the presence of 50μM NBDG (ThermoFisher N13195) before staining with live/dead (AquaFluorescent amine-reactive dye, Invitrogen) and IL7R (BD Biosciences, Clone HIL-7R-M21) and acquisition of NBDG fluorescence on a BD LSR Fortessa instrument.

### Glutaminolysis inhibition

Following isolation as above, CD8^+^ T cells were stimulated and co-cultured in the presence of either a dose range of sodium valproate (Sigma) or of the specific glutaminolysis inhibitors GDH1 (Focus Biomolecules), C968 (Sigma) or BPTES (Sigma), or with the cell-permeable α-ketoglutarate analogue dimethyl-2-ketoglutarate (DMKG, Sigma) as indicated.

### Metabolomic data

Primary human CD8^+^ T cells were snap frozen in liquid nitrogen following isolation, stimulation and culture as described above in the presence of either apicidin (1.4 nM), valproate (87.5μM) or vehicle control ( < 0.001% DMSO, n = 8 per group). Cell pellets were subjected to LC-MS/MS analysis (Metabolon Ltd). In brief, **s**amples were prepared using the automated MicroLab STAR® system (Hamilton) with recovery standards added prior to extraction for QC purposes. Proteins were precipitated with methanol under vigorous shaking for 2 min (Glen Mills GenoGrinder 2000) followed by centrifugation and the resulting extract was divided into five fractions: two for analysis by two separate reverse phase (RP)/UPLC-MS/MS methods with positive ion mode electrospray ionisation (ESI), one for analysis by RP/UPLC-MS/MS with negative ion mode ESI, one for analysis by HILIC/UPLC-MS/MS with negative ion mode ESI, and one sample was reserved for backup. Samples were placed briefly on a TurboVap® (Zymark) to remove the organic solvent. Sample extracts were stored overnight under nitrogen before preparation for analysis. Spiked external QC standards were used as controls with water as negative controls and a pool of well-characterised human plasma as positive control. All methods utilised a Waters ACQUITY ultra-performance liquid chromatography (UPLC) and a Thermo Scientific Q-Exactive high resolution/accurate mass spectrometer interfaced with a heated electrospray ionisation (HESI-II) source and Orbitrap mass analyzer operated at 35,000 mass resolution.

### RNA sequencing

Primary CD8^+^ T cells were cultured and stimulated as above for 6 days in the presence of either sodium valproate (175μM, Sigma) or sodium valproate plus R162 (175μM/25μM, Focus Biomolecules) before total RNA was extracted using an RNeasy mini kit (Qiagen) with quality assessed using an Agilent BioAnalyser 2100 and RNA quantification performed using a NanoDrop ND-1000 spectrophotometer. Sequencing library preparation was performed using the SMARTer stranded Total RNA-Seq kit v2 pico input (Takara Bio) and 75 bp paired end sequencing performed on a NextSeq500 instrument with titrated 1% PhiX. Raw sequence read QC was undertaken using fastqc followed by adapter removal (TrimGalore), extraction of ribosomal reads (BBsplit) and sequence mapping (HISAT2) to the reference genome (GRCh38). Merged bam file QC was undertaken using the QoRTs package in R with assimilation using multiQC.

### ATAC sequencing

ATACseq was undertaken following the protocol established by Buenrostro et al.^103^ with modifications. Following CD8^+^ T cell culture as above, 50,000 cells were lysed (1 M Tris-HCl) and the nuclear pellet transposed using the Tn5 transposase (TDE1, Illumina) at 37 °C for 30 min before DNA isolation (Qiagen minElute) and PCR amplification (NEBnext) guided by qPCR (KAPA-SYBR) estimation of reporter signal v cycle number and PCR cycle length tailored to 1/3 maximal amplification. Libraries were purified (AMPure XP) with quality assessed using an Agilent BioAnalyser 2100 and RNA quantification performed using a NanoDrop ND-1000 spectrophotometer. 50 bp paired-end sequencing was performed on a NextSeq500 instrument with downstream QC matching against ENCODE standards.

### in vitro restimulation

Primary CD8^+^ T cells were isolated, stimulated and cultured as above for 6 days in the presence of either sodium valproate (175μM, Sigma) or vehicle control ( < 0.001% DMSO) followed by flow cytometric sorting (FACSAriaIII cell sorter, BD Biosciences) into divided CFSE^lo^IL7R^hi^ and CFSE^lo^IL7R^lo^ subpopulations. These were rested for 5 days in complete RPMI-1640 (Sigma), with normalisation of live cell numbers (1×10^6^/ml) and CFSE-relabelling before polyclonal restimulation repeated as for the primary stimulation (anti-CD2/3/28, Miltenyi). After 5 days restimulation cells were analysed by flow cytometry (BD LSR Fortessa) with staining for live/dead (AquaFluorescent amine-reactive dye, Invitrogen) and IL7R (BD Biosciences, Clone HIL-7R-M21).

### Expression and purification of SARS-CoV-2 RBD

Recombinant SARS-CoV-2 Spike RBD glycoprotein carrying a C-terminal hexahistidine purification (His_6_) tag was expressed in the *Pichia pastoris* X-33 yeast strain using the pPICZα A expression vector (ThermoFisher). Cultures were grown on the 10-liter scale in an Eppendorf BioFlo 510 fermenter with active control of temperature, pH, dissolved oxygen concentration and methanol feed (as the carbon source) to maximise protein yield based on a protocol developed previously to purify SARS-CoV-1 RBD^104,105^. RBD glycoprotein was secreted into the cell culture supernatant and purified by nickel-affinity and size-exclusion chromatography. To produce untagged RBD, the His_6_ tag was removed using Carboxypeptidase A (Sigma), followed by nickel-affinity chromatography to remove uncleaved protein and size-exclusion chromatography.

### Tissue processing

Splenocytes and draining lymph nodes were weighed and then homogenised through a 70 μm cell strainer (BD bioscience) using complete tissue culture media [IMDM (Thermo), 10% Foetal Bovine Serum (Sigma), 1 mM Sodium Pyruvate (Thermo), 1 X Non-essential Amino Acid Solution (Sigma), 2 mM Glutamax (Thermo), 100U/ml Penicillin-Streptomycin (Thermo) and 50μM β-Mercaptoethanol (Sigma)]. Splenocyte solutions were red blood cell lysed, resuspended in culture media and counted before use.

### ELISPOTs

For B cell ELISPOTS, MultiScreen-IP Filter PDVF, 0.45 µm ELISPOT plates (Merck Millipore) were first activated in 70% ethanol for 2 minutes before being washed twice with sterile PBS. Plates were then coated overnight at 4 °C with 50μl of purified RBP protein in PBS (5μg/ml). The next day, plates were washed twice with sterile PBS and blocked with 200μl complete tissue culture media for 2 h at 37 °C. After this time, blocking media was aspirated and 100μl of splenocyte suspensions were added in two concentrations (10^6^/ml and 10^5^/ml) to appropriate triplicate wells. Plates were then incubated at 37 °C overnight. After 18hrs cells were aspirated from the wells and plates were washed 3 times with PBS/0.05% Tween-20 (Sigma) [PBST] followed by 3 washes in PBS. 50μl of goat anti-mouse IgG-HRP conjugate (Southern Biotech) in PBS at 1:2000 dilution was then added to the plates and incubated at room temperature for 1 h. Plates were then washed as previous, before being developed with 3-Amino-9-ethylcarbazole tablets (Sigma) using the manufacturer’s instructions. After stopping development, plates were left to dry for 48 h before being read and analysed using an ELISPOT plate scanner (ELISPOT Reader System, AID) and spots enumerated using ImageJ (National Institutes of Health).

For IFNγ ELISPOTS, plates were prepared with ethanol and washed as above before being coated with 50μl of anti-mouse IFNγ (AN18, Thermo) at 2 μg/ml in PBS. Plates were incubated overnight at 4 °C before being washed and blocked as described above. First, 50μl of culture media containing 60U/ml IL-2 (Peprotech) and 20μg/ml purified RBP protein were added to the appropriate wells, followed by 50μl of cell suspension corresponding to 10^4^ or 10^5^ cells per well. Plates were then incubated at 37 °C for 48 h before cells were aspirated and washed as above. 50μl of biotinylated anti-mouse IFNγ (R4-6A2, Thermo) in PBS/0.5% BSA (Fischer) was then added to each well and plates were incubated for 2 h at room temperature before being washed. 50μl of Streptavidin-horseradish peroxidase (Thermo) diluted 1:5000 in PBS/0.5% BSA was then added to each well and plates were incubated for 1 h at room temperature, washed and then developed as above.

### Immunophenotyping

After harvesting cell suspensions were normalised to 1×10^6^/ml and stained with non-limiting concentrations of IL7R (CloneA7R34, Biolegend), PD1 (Clone RMP1-30, Biolegend), KLRG1 (Clone 2F1/KLRG1, Biolegend), CD44 (CloneIM7, Biolegend), NP-dextramer (Immudex), GL7 (Clone GL7, Biolegend), CD8 (Clone 53-6.7, Biolegend), BCL6 (CloneIG191E/A8, Biolegend), FOXP3 (Clone FJK-16s, ThermoFisher), B220 (Clone RA3-6B2, Biolegend) before data acquisition by flow cytometry (BD LSR Fortessa) and analysis in FlowJo (BD Biosciences) and prism 6.0 (Graphpad).

### Multiplex IHC

Lung and spleen samples were fixed and processed into FFPE blocks. 4.5um paraffin sections were collected on Super Frost Plus slides and dried overnight. For immunostaining slides were deparaffinized in xylene and rehydrated with series of graded ethanol. Antigen Retrieval was performed by gentle simmering in 700 ml of Tris-EDTA, pH9 buffer, following by endogenous peroxidases blocking and incubation with 2.5% Horse Serum. Ki67 antibody (1:500) were incubated in a humidified chamber overnight at +4 C, detected using ImmPRESS anti-Rabbit-HRP Polymer detection system (VectorLab, MP-7401) and visualised using TSA-Plus Opal 620 (Akoya, FP1495001KT). Slides were then placed in Tirs-EDTA, ph9 buffer and simmered for 15 min. anti-CD3 antibodies (1:50) were applied and incubated in a humidified chamber overnight at +4 °C. As before primary anti-CD3 antibodies were detected with ImmPRESS anti-Rabbit-HRP Polymer detection ssytem and visualised with TSA-Plus Opal 520 (Akoya, FP1495001KT). Slides were mounted with Dapi-supplemented ProlongGold and imaged using Zeiss Apotome 2 microscope and Hamamatsu monochrome camera.

### SARS-CoV-2 neutralisation assay

The virus used in this study was the clinical isolate SARS-CoV-2/human/Liverpool/REMRQ0001/2020, a kind gift from Ian Goodfellow (University of Cambridge), isolated by Lance Turtle (University of Liverpool) and David Matthews and Andrew Davidson (University of Bristol)^106,107^. Sera were heat-inactivated at 56 °C for 30 mins, then frozen in aliquots at −80 °C. Neutralising antibody titres at 50% inhibition (NT50s) were measured as previously described^73^. In brief, HEK293T reporter cells expressing ACE2, Renilla luciferase (Rluc) and SARS-CoV-2 Papain-like protease-activatable circularly permuted firefly luciferase (FFluc) were seeded in flat-bottomed 96-well plates. The next day, SARS-CoV-2 viral stock (MOI = 1) was pre-incubated with a 3-fold dilution series of each serum for 2 h at 37 °C, then added to the cells. After 24 h, cells were lysed in Dual-Glo Luciferase Buffer (Promega) diluted 1:1 with PBS and 1% NP-40. Lysates were transferred to white half-area 96-well plates, and infectious virus quantitated as the ratio of FFluc/Rluc activity measured using the Dual-Glo kit (Promega) according to the manufacturer’s instructions. To obtain NT50s, FFluc/Rluc ratios were expressed as fraction of maximum, then analysed using the Sigmoidal, 4PL, X is log(concentration) function in GraphPad Prism. For visualisation and ranking, samples with no detectable neutralising activity at the lowest dilution tested (1:30) were assigned an arbitrary NT50 of 10. Experiments were performed in duplicate and repeated at least once.

### BCR sequencing

#### Library Preparation

Whole blood RNA was extracted from PAXgene Blood RNA tubes (BD Biosciences). B cell receptor repertoire libraries were generated using the protocol described by Bashford-Rogers et al. ^108^. Briefly 200 ng of total RNA (14ul volume) was combined with 1uL 10 mM dNTP and 10uM reverse primer mix (2uL) and incubated for 5 min at 70 °C. The mixture was immediately placed on ice for 1 minute and then subsequently combined with 1uL DTT (0.1 M), 1uL SuperScriptIV (Thermo Fisher Scientific), 4ul SSIV Buffer (Thermo Fisher Scientific) and 1uL RNAse inhibitor. The solution was incubated at 50 °C for 60 min followed by 15 min inactivation at 70 °C. cDNA was cleaned with AMPure XP beads and PCR-amplified with a 5′ V-gene multiplex primer mix and 3′ universal reverse primer using the KAPA protocol and the following thermal cycling conditions: 1cycle (95 °C, 5 min); 5cycles (98 °C, 20 s; 72 °C, 30 s); 5cycles (98 °C, 15 s; 65 °C, 30 s; 72 °C, 30 s); 19cycles (98 °C, 15 s; 60 °C, 30 s; 72 °C, 30 s); 1 step (72 °C, 5 min). Sequencing libraries were prepared using Illumina protocols and sequenced using 250-bp paired-end sequencing on a MiSeq system (Illumina).

### Reporting summary

Further information on research design is available in the Nature Portfolio Reporting Summary linked to this article.

## Supplementary information

Supplementary Information

Description of Additional Supplementary Files

Supplementary Data 1

Supplementary Data 2

Supplementary Data 3

Supplementary Data 4

Supplementary Data 5

Supplementary Data 6

Reporting Summary

Transparent Peer Review file

## Source data

Source data

## Data Availability

RNA-seq data have been deposited in EBI ArrayExpress and are publicly available as of the date of publication under the accession numbers: E-MTAB-13366 (RNA-seq of isolated primary human CD8 + T cells treated with KDACi with polyclonal stimulation for 6 days), E-MTAB-13367 (ATAC-seq of isolated primary human CD8 + T cells treated with KDACi with polyclonal stimulation for 6 days), E-MTAB-13368 (ATAC-seq of CD44 + CD8 + T cells isolated from spleen on 5 post rechallenge in a heterotypic influenza rechallenge model). Additional raw datasets and full protocols have been deposited at Mendeley and are publicly available as of the date of publication (https://data.mendeley.com/drafts/pncgztw2mg). Accession codes and DOI are also listed in Supplementary Table 1. Source data are provided with this paper.
