# Supplementary material for: Multiomic analysis identifies glutaminolysis-dependent metabolic enhancement of immune memory utilised for vaccine development
Source: Nat Commun. 2026 Jun 26;17:8018. doi: 10.1038/s41467-026-74866-4 (PMC13454449; doi:10.1038/s41467-026-74866-4)
Supplement: Supplementary file 1 [file 41467_2026_74866_MOESM10_ESM.pdf]

Peer Review File

Multiomic analysis identifies metabolic enhancement of immune memory by enhanced glutaminolysis in cells, mice and humans

Corresponding Author: Professor Eoin McKinney

This file contains all reviewer reports in order by version, followed by all author rebuttals in order by version.

Version 0:

Reviewer comments:

Reviewer #1

(Remarks to the Author)
In an innovative approach, the authors screened transcriptional signatures of T cell memory and exhaustion against repurposable drug response signatures. Using drug response signatures evoked in defined tumor cell lines, they identifed several lysine deacetylase inhibitors (KDACi) that promote a memory precursor phenotype in primary T cells both in an in vitro T cell activation assay and in vivo in various infection/immunization models. Multiomic studies led to the identification of enhanced glyutaminolysis as the mechanism underlying this memory-repogramming activity. They also provide evidence for a strong adjuvant activity of a KDACi (valproate at doses lower than those required for anti-convulsant therapy) in human memory T and B cells undergoing seasonal influenza vaccination.
This is well written manuscript that uncovers a new class of compounds with the ability to significantly enhance memory immune cell differentiation when activated/primed in the presence of the compound for a short period of time. It provides strong mechanistic data supporting glutamine anaplerosis as the main supply of acetyl for epigenetic imprinting of a memory gene expression program in activated T cells, as the main cell type characterized in detail. These findings are important and have key implications for pharmacological intervention to improve immunotherapies such as therapeutic cancer vaccines and adoptive cell transfer data. It is even suggested, importantly, a role of repurposable drugs, such as sodium valproate, in making prophylactic vaccination more efficient in the elderly.
The data from the human study are encouraging and a little bit puzzling. The latter if considering the results from the in vitro treatment assays which indicate that the main targets are naive T cells (figure 1G) rather than the pre-existing memory pool of T cells. It is hard to imagine that the influenza vaccine booster of both antigen specific T and B cells is operating at the naive immune cell level. How do the authors reconcile this potential disconnect ? Have they tested the impact of valproate on highly pure naive and memory T cells ?
The use of valproate in vaccination in the elderly is an interesting application. The authors should consider evaluating the effect of valproate on T cells from young adutls compared with T cells from >80 year old donors.
Minor points :
Figure 1B : please explain the meaning of the circle sizes (range = 2.5 – 10.0, 4 different size values)
Line 107 – markers (makers)
Lines 210 – 212 : figure 3F appears to be quoted erronously as the 3F panel depicts a schema of pathway enrichment approach to ATACseq. It might be figure 3D, instead ?
Figure 4G : there is also an increase of the SLECs, not only the MPECs, which is also dependent on anaplerosis. This should be discussed.
Figure 4H : it is not clear which scatter groups depict IL7R+ and which PD-1+ cells. Presentation should be improved both in the figure and in the corresponding legend.
Line 265 : a likely effect of valproate on other cell types. Please elaborate.
Figure 6E : the antibody titers ratios reported in this panel might be in IgG log2 and not log10. Please check. The scatter plots in 6F, showing summary titers on day 7/baseline ratio seems more realistic (magnitude of change) than in figure 6E
Figure S8 A, B : the figure legend refers to days 1 to 7 but the X-axis labels says days 0, 7 and 30. Which is the true case represented here ? If the latter (the X-label is correct), how does it differentiate from the data represented in figure 6C ?
Line 316 : probably refers to figure 6E, F and not as printed figure 5E, F
Line 323 : « Treatment on the global BCR repertoire … Seems like an incomplete sentence, not clear what it is saying …
Tables S1 to S3 need legend explaining the nature of the displayed gene signatures, for the non-specialist reader.

Reviewer #2

(Remarks to the Author)
In this manuscript, Sowerby et al. investigate whether repurposing various pharmaceuticals can boost memory T cell formation and enhance memory responses. The authors identify selective inhibitors for lysine deacetylases (KDACi) that can boost memory precursor progenitor cell (MPEC)-like cell formation of human and murine CD8+ and CD4+ T cells in vitro, and the corresponding effects of KDACi on immune memory responses in multiple animal models and in a human vaccination trial. Such effects appeared to be attributed to enhanced generation of alpha-ketoglutarate and were dependent upon the activity of glutamate dehydrogenase (GLUD1). These results suggest that KDACi may represent a means to improve vaccine efficacy and boost immune memory responses, in part via metabolic rewiring of T cells, which will be of interest to immunologists and clinicians studying vaccine design and metabolic control of immunity. Several points should be addressed prior to publication:

Major comments

1.The authors postulate that increased glutaminolysis in the KDACi-induced effects on memory T cell responses in vitro and in vivo. However, they only test the contribution of glutamate dehydrogenase (GLUD1) and the generation of alpha-ketoglutarate rather than the glutaminolysis pathway.

a.The authors mention that other “glutaminolysis proximal inhibitors” had no effect, though data testing the contribution of other glutaminolysis-related inhibitors do not appear to be provided in the manuscript. The authors should show how glutaminase (GLS) and GOT1 inhibition impacts memory T cell generation, at minimum in vitro using both mouse and human T cells, as this will further highlight the specificity of glutamate-derived alpha-ketoglutarate in this process. Overall, the role of the glutaminolysis pathway versus the specific GLUD1-dependent generation of alpha-ketoglutarate should be resolved.

b.Can acute restriction of glutamine and/or glutamate also rectify the increased memory potential within the in vitro system?

c.Are the glutaminolysis-related enzymes (GLUD1, GLS, GOT1) differentially upregulated in KDACi-treated human and mouse CD8+ T cells?

2.What is the impact of GLUD1 inhibition alone in both the in vitro and in vivo (in particular, the IAV infection model) systems? This treatment group should be included, as GLUD1 activity has recently been reported in memory CD8+ T cells (Tang et al. 2023 Nat Immunol). Furthermore, certain studies suggest that glutamine uptake (Nabe et al. 2018 Cancer Sci; Huang et al. 2021 Cell) and GOT1 (Xu et al. 2023 Cell Rep) may be inhibitory for memory T cell generation.

3.Overall, limited surface markers were used to designate cells as MPEC/memory populations (mainly IL-7R) from activated/exhausted-like populations (mainly PD-1) in both the in vitro and in vivo systems. While this reviewer appreciates the inclusion of gene signatures, additional memory- versus effector T cell-related markers could be included to better support the conclusions of the study. This would be especially pertinent in the IAV infection model (both primary and re-challenge infections), where the authors could distinguish naïve, effector, effector/memory (Tem), central memory (Tcm), and tissue-resident memory (Trm) populations in the spleen, mediastinal lymph node, and lung.

4.Based on Figure 3E, there was no enrichment for memory-related signatures in LF7, contrary to the authors’ conclusion. The Day15_Eff_vs_memory_CD8_Tcell signature is an effector signature. Please check other T memory-related signatures from the C7 Immunological Collections of the Molecular signatures database to better support this conclusion, or revise the statement (page 8, lines 205–206).

5.For the ATAC-seq datasets, the authors should perform peak set enrichment analysis (PSEA) using the T cell-related genes signatures as in Fig. 3D,E, as well as those from the C7 Immunological Collections of the Molecular Signatures Database to expand the analysis of T cell-related signatures. In addition, the authors should show a heatmap of the differentially accessible peaks, along with the expression of selective effector or memory-related genes. Both of these analyses would better support the notion that memory-related epigenetic programs are altered upon KDACi treatment. Transcription factor activities can also be predicted from ATAC-seq data using transcription factor footprinting or motif enrichment analyses. In particular, it would be interesting to see if Myc activity was also predicted to be enhanced in the ATAC-seq data.

6.The scatterplots shown for the inhibitor screening data were often difficult to discern conclusions (e.g., those in Fig. 1E–G). Please include the raw data about the impacts of each of the drugs, with names listed, on each of the parameters tested (IL7RA, PD-1, cell proliferation, CD25, and survival) to complement these figures.

7.For the pathway enrichment and metabolite-set enrichment analyses, it was unclear as to whether all differentially expressed genes/metabolites were included or if they were separated into upregulated versus downregulated. Separating them would help better discern whether a given gene signature was increased or decreased in each comparison and aid with interpretations.

Minor comments

1.The readability of this manuscript could be improved via careful editing of the figure callouts, as many seemed to be incorrect (e.g., data for CD25 and cell survival were not shown in Fig. 1D, E) and/or disorganized in the text (e.g., panels in Fig. 4 were cited out of the more logical, sequential order).

2.For Table S3, it would be helpful to more precisely identify the target of the inhibitors rather than calling them “molecules”. In particular, the different inhibitors of KDACs should be clearly labeled.

3.The incorporation of diagrams to describe experimental systems was appreciated, although the authors should also add more experimental details to their figure legends. It was challenging, at times, to match the descriptions in the Methods with the experimental systems shown in each of the figure panels.

4.The labels for Figure 4H were not clear – please illustrate which plots designate IL-7R MFI (left plots) and which designate PD-1 MFI (right plots). Similarly, in addition to the figure legend, please provide a legend key to illustrate what the orange versus blue bars refer to for Fig. S2C.

5.For the Seahorse data, it is unclear why relative values are shown and how the data were normalized. Please clarify this point and more ideally, show the raw values for such assays.

6.The authors should carefully revise conclusions about the impact of beta-catenin and Myc signatures in their model. The data provided in the paper are correlative only (page 9, lines 233–235). Similarly, GLUD1 inhibition in the in vivo models only partially rescued selective phenotypes, so those conclusions should be carefully reviewed and revised as needed (e.g., page 11, lines 271–274). Finally, the authors should not call PD-1 an exhaustion marker in this context (page 4, line 93), as PD-1 is also expressed by activated T cells.

Reviewer #3

(Remarks to the Author)
The study by Sowerby and colleagues describes the ability of KDAC inhibitors to promote T cell memory responses after vaccination. It offers interesting insights derived from cell line and mice experiments, identifies enhanced glutaminolysis as the responsible mechanism, and reports on the results of a human challenge study which investigated the effects of vaproat, a repurposable KDACi, after seasonal influenza vaccination. These data are interesting and add to the increasing body of literature investigating the connection between immunometabolomics and vaccine responses.

Some comments and questions:

- Line 165-166: Selective blockade of glutaminolysis using the GLUD1 inhibitor R162 (but not more proximal inhibitors, Supp Fig3A)…” However, in Supp Fig3A. only DMKG is shown which is not an inhibitor but, as described in the next phrase, would enhance metabolic flux through the TCA cycle. Which proximal inhibitors were used and, in the authors' opinion, why did they not have the same effect as more distal blockers?
- Transcriptomics: The DEG analysis is only described in a cursory fashion in the methods section and should be described in more detail there. Were low-abundance genes removed prior to the DEG analysis? If so, how many were excluded? The two conditions to be compared were not independent, as the same cell line was used in two different states (with and without treatment, i.e., a paired design). How was this accounted for in the EdgeR workflow? Additionally, only reporting the number of DEGs does not convey much information to the reader. What kind of genes were up- and down-regulated? A functional annotation using gene ontology or alike would be helpful.
- Figure: 3: Figure 3A is referenced in connection to differentially expressed genes (line 191), but does not contain anything on DEGs but is a schematics on MOFA. Confusingly, Figure 3 therefore jumps from MOFA (A) to DEGs (B) back to MOFA (C). In B) in the lower panel, it is very confusing to color genes in red/blue which according to the figure legend indicate statistical significance, even when the genes are clearly below the significance threshold. If the same genes that are significant in the upper panel should be labelled, a different color should be used. However, I am not really convinced that this adds any useful information because clearly, not a single gene is differentially expressed in the lower panel; therefore all significant genes from the upper panel must be below the threshold and insignificant in the lower panel.
- "MOFA was used “to interpret these gene expression changes” (line 193). This could also rather easily be done with e.g. GO annotation or BTMs etc. Why did the authors choose to use MOFA and what kind of insights does this more complex approach offer that e.g. a GO or BTMs/BloodGen3Modules would not provide? As far as I know, the usual application and strength of MOFA is to combine different –omics datasets for the joint analysis of multiple datasets. As many datasets are available in this study, have the authors considered performing this kind of integrative analysis?

- Human challenge study: The study protocol specifies that the aim was to recruit 180 participant based on a given power calculation. However, in the paper, only 69 participants were recruited to the study, less then half. Why? How would this impact the results of the study? This should be acknowledged in the paper.

- How are the baseline characteristics distributed across the randomized groups? Figure S7 (not S6 as written in line 847) only shows figures for age and sex. These, however, were the two variables included in the minimization algorithm and it is therefore expected that these are balanced (assuming that the manimimzation algorithm worked correctly). According to the protocol, several other characteristics that are associated with vaccine responses were also captured for each participant, such as BMI, smoking, and prior vaccination history. A detailed baseline characteristics table is required comparing the

distributions of these relevant variables including the pre-vaccination titers/ T cell frequencies across studygroups.

- Two different drug dosages (100mg and 200mg valproate) were used in the study and according to the study protocol, they were to be analysed separately. In the paper, without explanation, these are pooled together. Why? Dose-dependent relationships would give more information about the results and/or provide insights if no such relationship is seen. Accordingly, in Figure 6 C, D, E etc. two different colors should be used for the two dosages.

- The study protocol states in the analysis plan, that linear and logistic regression models will be used to explore the association between study endpoints and variables including age, drug dose, and prior memory responses. This has so far not really been addressed yet but would be important. Additionally, on multiple occasions the study protocol mentions age stratification into 18-65yo and >65 yo but this has not been explored in the paper.

- Only ratios are displayed in Figure 6 A, D, E. How would absolute values look like? An ANCOVA analysis with adjustment for prior titer/cell frequency levels would be less prone to regression to the mean effects while still be able to adjust for baseline levels.

- Have adverse effects been recorded? The dose was low and the study was not registered as a clinical trial, but vaproat does have serious potential side effects and it would be important to include these.

- A key resources is mentioned on multiple occasions in the paper, e.g., for GEO accessions numbers etc. However, I could not find this table.

- Abbreviation "ob" / "bd" in Figure S7 should be explained in the legend. Preferably, it is just avoided, there would be enough space to write it out like 1x/day and 2x/day.

Version 1:

Reviewer comments:

Reviewer #1

(Remarks to the Author)
The authors have satisfactorily addressed the comments and poised raised by this reviewer.

Reviewer #2

(Remarks to the Author)
The authors performed new analyses and/or clarified points in response to my original comments. The study will be an important advance in understanding mechanisms to boost memory immune responses and the contribution of metabolic rewiring to such a process. A few minor comments remain to be addressed:

1. While the functional impacts of valproate treatment in improving secondary recall responses and vaccine responses are striking, the original question remains about the type(s) of memory T cell responses that are being generated in response to valproate treatment (Tcm, Tem, or Trm). The analyses of the cell populations at day 30 before re-challenge in the IAV infection model would help to clarify the points on lines 276–279 regarding whether those cells that are expanded are tissue-resident or circulating (Tcm or Tem) populations. This reviewer feels that such analyses would still be a beneficial addition to this paper, given the differential effects of distinct memory T cell populations on long-term immunity in tissues. At minimum, this point should be acknowledged as a limitation of this study, especially in the human patient cohort.

2. Based on the authors' response to the original comment #4, please revise the names in Fig. 3E to match exactly what is shown in MSigDB, especially to reflect the use of DN or UP at the end of the signature. For example, it is presumed, based on the authors' response, that the Day15_Eff_vs_Memory_CD8_Tcell signature is the Kaech_Day15_Eff_vs_Memory_CD8_Tcell_DN signature, which is a memory T cell signature. Without this additional information, this reviewer interprets the signature to show genes enriched in effector cells rather than memory cells. The same applies to all the other signatures listed.

3. The label is missing above the bubbles in Fig. 3D.

4. For the new experimental schematic in Fig. S3A, the authors should revise to GLUD1 rather than its alternative name of GDH1. This will aid the readers of the manuscript, since GLUD1 is the name used throughout the text.

5. Please add a statement to the methods regarding how the Seahorse data were normalized (as described in the response to minor comment #5). This point remains unclear in the revised manuscript.

6. In the discussion (and related to the authors' response to comment #1), the authors could clarify that glutamine and glutaminolysis have additional effects beyond T cell activation, citing those papers mentioned in the original comment #2 (Tang et al. 2023 Nat Immunol; Nabe et al. 2018 Cancer Sci; Huang et al. 2021 Cell; Xu et al. 2023 Cell Rep), as well as the notion that glutamine deprivation or glutaminase inhibition can have differential effects on CD4 and CD8 T cell populations (Nakaya et al. 2014 Immunity; Johnson et al. 2018 Cell). This provides broader context to the readers about the overall

impact of altering glutamine metabolism on T cell responses, especially in consideration of the newly included data on valproate treatment alone in the IAV model.

Reviewer #3

(Remarks to the Author)
My comments have been adequately addressed. In my opinion, this work is now suitable for publication.

Version 2:

Reviewer comments:

Reviewer #2

(Remarks to the Author)
The authors have addressed the majority of my remaining minor comments. The study is acceptable for publication.

Reviewer #3

(Remarks to the Author)
In my opinion, this work is now suitable for publication.

Open Access This Peer Review File is licensed under a Creative Commons Attribution 4.0 International License, which permits use, sharing, adaptation, distribution and reproduction in any medium or format, as long as you give appropriate credit to the original author(s) and the source, provide a link to the Creative Commons license, and indicate if changes were made.
In cases where reviewers are anonymous, credit should be given to 'Anonymous Referee' and the source.
The images or other third party material in this Peer Review File are included in the article's Creative Commons license, unless indicated otherwise in a credit line to the material. If material is not included in the article's Creative Commons license and your intended use is not permitted by statutory regulation or exceeds the permitted use, you will need to obtain permission directly from the copyright holder.
To view a copy of this license, visit https://creativecommons.org/licenses/by/4.0/

REVIEWER COMMENTS
[responses highlighted in red; line references to amended manuscript version; updated text in amended manuscript version also highlighted in red]

Reviewer #1 (Remarks to the Author):
In an innovative approach, the authors screened transcriptional signatures of T cell memory and exhaustion against repurposable drug response signatures. Using drug response signatures evoked in defined tumor cell lines, they identifed several lysine deacetylase inhibitors (KDACi) that promote a memory precursor phenotype in primary T cells both in an in vitro T cell activation assay and in vivo in various infection/immunization models. Multiomic studies led to the identification of enhanced glyutaminolysis as the mechanism underlying this memory-repogramming activity. They also provide evidence for a strong adjuvant activity of a KDACi (valproate at doses lower than those required for anti-convulsant therapy) in human memory T and B cells undergoing seasonal influenza vaccination.
This is well written manuscript that uncovers a new class of compounds with the ability to significantly enhance memory immune cell differentiation when activated/primed in the presence of the compound for a short period of time. It provides strong mechanistic data supporting glutamine anaplerosis as the main supply of acetyl for epigenetic imprinting of a memory gene expression program in activated T cells, as the main cell type characterized in detail. These findings are important and have key implications for pharmacological intervention to improve immunotherapies such as therapeutic cancer vaccines and adoptive cell transfer data. It is even suggested, importantly, a role of repurposable drugs, such as sodium valproate, in making prophylactic vaccination more efficient in the elderly.

The data from the human study are encouraging and a little bit puzzling. The latter if considering the results from the in vitro treatment assays which indicate that the main targets are naive T cells (figure 1G) rather than the pre-existing memory pool of T cells. It is hard to imagine that the influenza vaccine booster of both antigen specific T and B cells is operating at the naive immune cell level. How do the authors reconcile this potential disconnect? Have they tested the impact of valproate on highly pure naive and memory T cells?
We are pleased that the Reviewer finds our approach innovative and thank them for highlighting this important point. Fig1G indicates that a greater response *in vitro* is observed with naïve cells (cells tested here are highly purified naïve and memory subets, sorted by FACS, Fig1G). The legend of Fig1G has been modified to make this clear (line 474).
However, we did also observe responses from Tem subsets in vitro, albeit to a lesser degree than for purified naïve cells (Fig1G).
We present (and now discuss in more detail) two other pieces of evidence that support an impact of treatment on both memory and naïve responses in humans.
First, VDJ sequence repertoire analysis after vaccination demonstrated treatment-driven increases in both breadth and expansion of the influenza-specific B cell response (Fig6G-N). This was observed in both naïve (IgM/IgD) and memory (class-switched) sequences (Fig6L, N), confirming that both are impacted by treatment consistent with what was observed for Tn and Tem populations in vitro in Fig 1G.

Second, we observed boosting of IgG titres to both vaccine-included (Fig6E) and other influenza serotypes (Fig6F, SuppFig8). This suggests expansion of pre-existing memory responses, although cross-reactivity with vaccine-included strains cannot be excluded. We agree with the Reviewer that the impact on pre-existing memory is likely to have the greatest functional relevance for vaccination, particularly in older individuals, and we have included extended discussion of this point in the revised manuscript (lines 376-387).

The use of valproate in vaccination in the elderly is an interesting application. The authors should consider evaluating the effect of valproate on T cells from young adults compared with T cells from >80 year old donors.
We thank the Reviewer and agree that the effect in older individuals is a potential application of our findings. This was considered during study enrolment but recruitment of those >65 years was precluded by the BioResource Advisory Board due to concerns over attendance at the hospital campus during the ongoing pandemic that persisted at the time of the study. We plan to investigate in older adults in future studies as suggested. The rationale for this restriction has been clarified in the methods section (lines 897-899).

<u>Minor points :</u>
Figure 1B : please explain the meaning of the circle sizes (range = 2.5 – 10.0, 4 different size values).
Circle diameter in Fig1B represents corrected significance (-$\log_{10}$adjP) and this has been annotated with a legend to improve clarity.

Line 107 – markers (makers)
Thank you: this has been corrected

Lines 210 – 212 : figure 3F appears to be quoted erroneously as the 3F panel depicts a schema of pathway enrichment approach to ATACseq. It might be figure 3D, instead?
Thank you: this has been corrected.

Figure 4G : there is also an increase of the SLECs, not only the MPECs, which is also dependent on anaplerosis. This should be discussed.
We thank the Reviewer for highlighting this point. The expansion of SLECs may indeed be due to enhanced anaplerotic support as suggested. We have included this in discussion (lines 272-275) and have also noted that enhanced memory differentiation during the primary response is expected to lead to increased SLEC production in the secondary response. It is more likely that the observed SLEC expansion during the secondary response is a consequence of enhanced memory formation during the primary than from a direct drug effect (ie enhanced anaplerosis). This is because drug treatment is only given during the primary response and the expansion is observed during the secondary, over 30 days later. However, our data cannot directly discriminate between these two possibilities and we have included discussion of the point as above.

Figure 4H : it is not clear which scatter groups depict IL7R+ and which PD-1+ cells. Presentation should be improved both in the figure and in the corresponding legend.

We agree this was insufficiently clear and are grateful to the Reviewer for highlighting this point. This has now been corrected in Fig 4H. We identified a similar problem with Fig4E which has also been amended to improve clarity of presentation.

Line 265 : a likely effect of valproate on other cell types. Please elaborate.
We thank the Reviewer for highlighting this important point. We have moved this comment to the discussion section with further elaboration included (lines 362-371):

"Our initial screen matched T cell signatures against drug signatures primarily generated in transformed cell lines. It is therefore inevitable that pathways conserved across cell types will be more easily identifiable. The causal mechanism identified – enhanced glutaminolysis – is conserved in many cell types and it is clear that treatment modulated CD4 T cell and B cell responses in vivo in addition to those in the CD8 T cells used for screening (Fig4L, FigS5). This is consistent with a likely effect of valproate treatment on other cell types (an effect we had also observed in vitro, FigS1I). Thus, although we set out to modulate T cell memory differentiation, B cell responses were also affected (although with no clear impact on myeloid phenotypes). This likely reflects a role for glutaminolysis facilitating proliferation and survival in other cell types[1-4], although we cannot exclude (and it is challenging to confirm) a possible impact on T cells with secondary modulation of B cell and antibody responses"

Figure 6E : the antibody titers ratios reported in this panel might be in IgG log2 and not log10. Please check. The scatter plots in 6F, showing summary titers on day 7/baseline ratio seems more realistic (magnitude of change) than in figure 6E
The data in Fig6E are ratios presented on a $\log_{10}$ scale and normalized to baseline values to show relative increase post-vaccination. The magnitude of fold increase in the control group is consistent with previous reports of post vaccine expansion in antibody titres[5,6]. Values in the treated group are significantly higher, although the majority still reside in the range of previously reported responses.
We agree that the axis label may have been confusing and have modified it to avoid this: data are ratios on a $\log_{10}$ scale rather than $\log_{10}$ ratios.
Note that raw (unnormalized) titres are also provided in Supp Fig 8C, E to allow further interpretation.

Fig6F shows titres for 'flu strains not included in the vaccine, some of which show expansion after vaccination and with valproate treatment, albeit to an expectedly lower degree and with no impact on irrelevant (RSV) antigen response.

Figure S8 A, B: the figure legend refers to days 1 to 7 but the X-axis labels says days 0, 7 and 30. Which is the true case represented here? If the latter (the X-label is correct), how does it differentiate from the data represented in figure 6C?
Figures S8A and B show data at 3 timepoints (days 0, 7 and 30) and the axis labelling and legends have been corrected to make this clear.
Figure S8 presents raw data that is not normalized to baseline value (and is therefore distinct from what is shown in Fig6C). Baseline-normalized ratios are presented in the main figure as responses are known to vary by baseline exposure and relative increase compared to baseline is the more easily interpretable result. Inclusion of raw values in

Supplementary FigS8 allows direct interpretation of unnormalized values (as noted by Reviewer 3 also).

Line 316 : probably refers to figure 6E, F and not as printed figure 5E, F
We thank the Reviewer for highlighting this error which has been corrected.

Line 323 : « Treatment on the global BCR repertoire … Seems like an incomplete sentence, not clear what it is saying …
We thank the Reviewer for highlighting this omission which has been corrected, now reading *"we analysed changes in the global BCR repertoire, finding an expected increase in somatic hypermutation and a decrease in diversity after vaccination (FigS9) and with no effect of treatment on the global BCR repertoire (Fig6I, FigS9)."*

Tables S1 to S3 need legend explaining the nature of the displayed gene signatures, for the non-specialist reader.
Thank you: we have added table legends which we hope now improves the clarity of the presentation.

Reviewer #2 (Remarks to the Author):
In this manuscript, Sowerby et al. investigate whether repurposing various pharmaceuticals can boost memory T cell formation and enhance memory responses. The authors identify selective inhibitors for lysine deacetylases (KDACi) that can boost memory precursor progenitor cell (MPEC)-like cell formation of human and murine CD8+ and CD4+ T cells in vitro, and the corresponding effects of KDACi on immune memory responses in multiple animal models and in a human vaccination trial. Such effects appeared to be attributed to enhanced generation of alpha-ketoglutarate and were dependent upon the activity of glutamate dehydrogenase (GLUD1). These results suggest that KDACi may represent a means to improve vaccine efficacy and boost immune memory responses, in part via metabolic rewiring of T cells, which will be of interest to immunologists and clinicians studying vaccine design and metabolic control of immunity. Several points should be addressed prior to publication:

<u>Major comments</u>
1.The authors postulate that increased glutaminolysis in the KDACi-induced effects on memory T cell responses in vitro and in vivo. However, they only test the contribution of glutamate dehydrogenase (GLUD1) and the generation of alpha-ketoglutarate rather than the glutaminolysis pathway.
The authors mention that other "glutaminolysis proximal inhibitors" had no effect, though data testing the contribution of other glutaminolysis-related inhibitors do not appear to be provided in the manuscript. The authors should show how glutaminase (GLS) and GOT1 inhibition impacts memory T cell generation, at minimum in vitro using both mouse and human T cells, as this will further highlight the specificity of glutamate-derived alpha-ketoglutarate in this process.
Overall, the role of the glutaminolysis pathway versus the specific GLUD1-dependent generation of alpha-ketoglutarate should be resolved.
Data describing the effect of proximal glutaminolysis inhibitors was presented in Supplementary Figure 3C-E. This included 3 inhibitors of glutaminolysis along with evidence that enhancing alpha-ketoglutarate (using the cell-permeable analog DMKG) could directly reproduce the effect seen in the in vitro phenotypic screen.
Specifically:
FigS3A: a new schematic illustration of glutaminolysis and inhibitor action to improve clarity of presentation and interpretation.
FigS3B: DMKG supplementation: addition of the cell-permeable analog of alpha-ketoglutarate dimethyl-ketoglutarate (DMKG) reproduced the phenotypic effect seen with KDACi treatment. This is not an inhibitor but confirms that increasing anaplerosis could reproduce the effect seen in the screening assay.
FigS3C: R162 (GLUD1 inhibitor) significantly reverses the memory phenotype when added to valproate-treated and stimulated T cells in vitro.
FigS3D: BPTES (GLS inhibitor) fails to reverse the memory phenotype when added to valproate-treated and stimulated T cells in vitro.
FigS3E: inhibitor C968 fails to reverse the memory phenotype when added to valproate-treated and stimulated T cells in vitro.

Anaplerosis is not the only mechanism by which glutaminolysis may alter cellular function with contributions also made to reactive oxygen species production, biosynthetic pathways and control of amino acid pools[4,7]. Inhibition of more proximal steps in the pathway also attenuate contribution to these processes, whereas distal inhibition specifically inhibits anaplerosis.
The fact that only distal inhibition of GLUD1 by R162 impacted on the observed phenotype indicates that anaplerosis was the likely mechanism being driven by glutaminolysis that was relevant for the phenotype in question. Our data are also consistent with previous reports of distinct effects of glutaminolysis inhibition when effected at different points of the pathway[8].

To make these points clear we have now included a schematic Figure (Supp Fig 3A) highlighting the points of inhibition, the alternative pathways that glutaminolysis can impact (depending on the level of inhibition) and additional discussion in the text (lines 170-179) in addition to the data previously presented showing the effect of other inhibitors.
We also note that our approach demonstrates both reversal of KDACi-induced effects on selective inhibition of glutaminolysis and induction of those effects on selective enhancement of anaplerosis (through DKMG). Although not an inhibitor of glutaminolysis as the Reviewer highlights, this supports our conclusion that it is the anaplerotic effect of glutaminolysis that is responsible (as DMKG directly provides the TCA intermediate α-ketoglutarate).

Can acute restriction of glutamine and/or glutamate also rectify the increased memory potential within the in vitro system?
Glutamine is an absolute requirement for T cell activation and is necessary for both proliferation and cytokine production[9]. Glutamine deprivation therefore results in failure of activation, proliferation and cytokine production and cannot be used to investigate phenotypic modulation of activated T cells.

Are the glutaminolysis-related enzymes (GLUD1, GLS, GOT1) differentially upregulated in KDACi-treated human and mouse CD8+ T cells?
Data showing expression of glutaminolysis-related enzymes is now included in SuppFig 3E with expanded discussion of their inhibition included as indicated above. We do not observe differential expression of glutaminolysis-related enzymes although differential expression is not a requirement for their differential activity which is also modulated through protein stability and activity directly[10-12]. We do see specific differential acetylation of glutaminolysis proteins along with differential metabolites as shown in Fig2C-G.

2.What is the impact of GLUD1 inhibition alone in both the in vitro and in vivo (in particular, the IAV infection model) systems? This treatment group should be included, as GLUD1 activity has recently been reported in memory CD8+ T cells (Tang et al. 2023 Nat Immunol). Furthermore, certain studies suggest that glutamine uptake (Nabe et al. 2018 Cancer Sci; Huang et al. 2021 Cell) and GOT1 (Xu et al. 2023 Cell Rep) may be inhibitory for memory T cell generation.

We thank the Reviewer for highlighting this important point. It is clear that glutamine restriction can block cellular (including T cell) proliferation, activation and cytokine production. Similarly, blocking glutaminolysis at different levels and indeed inhibition of key steps to different degrees (knockout versus suppression) may also produce different effects[8]. It is therefore important to interpret studies blocking glutamine uptake with care as these may impact on proliferation or activity which in turn impact directly on memory differentiation. Our study infers the impact of repurposed drugs that enhance glutaminolysis with no impact on activation or proliferation.
To clarify the impact of direct inhibition of glutaminolysis with GLUD1 inhibition in vivo we have now included data showing the impact of GLUD1 inhibition alone (the control group requested) in the IAV infection model (Supp Fig S6F). This confirms that glutaminolysis inhibition can suppress secondary responses when provided without Valproate. This is an expected result as endogenous glutaminolysis makes a contribution to T cell activation and proliferation even in the absence of valproate treatment (ie glutaminolysis is already active, our data suggest that it is simply further enhanced by KDACi treatment).
We also note that, in the in vitro models, we use GLUD1 inhibition in a dose-range down to levels that have no effect on cell activation or proliferation or survival (Supp 3B) with consistent effects on memory phenotype.

3.Overall, limited surface markers were used to designate cells as MPEC/memory populations (mainly IL-7R) from activated/exhausted-like populations (mainly PD-1) in both the in vitro and in vivo systems. While this reviewer appreciates the activated/exhausted-like populations (mainly PD-1) in both the in vitro and in vivo systems. While this reviewer appreciates the inclusion of gene signatures, additional memory- versus effector T cell-related markers could be included to better support the conclusions of the study. This would be especially pertinent in the IAV infection model (both primary and re-challenge infections), where the authors could distinguish naïve, effector, effector/memory (Tem), central memory (Tcm), and tissue-resident memory (Trm) populations in the spleen, mediastinal lymph node, and lung.

We thank the Reviewer for highlighting the importance of considering how memory is defined. We use different models to investigate different memory properties induced by KDACi treatment. Our in vitro primary T cell culture model necessarily uses a small number of markers as it is a high-throughput screen of many compounds at different doses. We follow this up by confirming recovery of the full memory signature used for in silico screening (Fig3). The *in vivo* models allow a more robust functional definition of memory, through testing for altered number, proportion, function and location of responding cells on secondary rechallenge following drug exposure during priming. This functional definition is standard across many models and does not rely on a small number of markers. Defining surface markers are included (analogous to those used in the high-throughput *in vitro* screen, Fig4I) to allow quantification of memory, naïve and memory subsets along with antigen-specific populations (Fig 4E, G, H, J, K). We therefore argue that we are not restricted to defining memory by a small number of markers in the in vivo models as suggested.
Note also inclusion of tissue sections showing differential number, proportion and location, so not restricted to a surface marker-based definition.

4.Based on Figure 3E, there was no enrichment for memory-related signatures in LF7, contrary to the authors' conclusion. The Day15_Eff_vs_memory_CD8_Tcell signature is an effector signature. Please check other T memory-related signatures from the C7 Immunological Collections of the Molecular signatures database to better support this conclusion, or revise the statement (page 8, lines 205–206).

The Reviewer is incorrect. Figure 3E shows clear, significant enrichment for multiple memory T cell signatures including multiple signatures differentiating effector and memory cells and exhausted and memory cells (Fig3E), which are taken from the C7 immunological collections as referenced in the methods and in Supplementary Table S6. Ten T cell differentiation signatures were selected to reflect T cell activation, memory differentiation and differentiation into T cell substates (Supp Table S6). Of these signatures, four showed significant enrichment in LF7, three of which comprise expression differences between memory/effector or memory/exhausted phenotypes. There is no enrichment of an activation signature comparing naïve and effector cells, which is instead enriched in another factor, LF1 that represents T cell activation. We maintain that the statement to this effect on p8 is therefore supported by the data and are unclear as to why the Reviewer finds otherwise. To improve clarity of presentation we have altered the callout in the Figure to avoid any confusion.

5.For the ATAC-seq datasets, the authors should perform peak set enrichment analysis (PSEA) using the T cell-related genes signatures as in Fig. 3D,E, as well as those from the C7 Immunological Collections of the Molecular Signatures Database to expand the analysis of T cell-related signatures. In addition, the authors should show a heatmap of the differentially accessible peaks, along with the expression of selective effector or memory-related genes. Both of these analyses would better support the notion that memory-related epigenetic programs are altered upon KDACi treatment.

We agree with the Reviewer that a Peak Set Enrichment Analysis is the optimal way to analyse this data for pathway enrichment. The data presented in Fig 3G presents the result of a peak set enrichment analysis undertaken against the KEGG pathway repository. We have now clarified this in the text to ensure that is clear (lines 232-235). The result of the enrichment is presented as a waterfall plot with the heatmap to the left indicating significance of enrichment of each pathway among differential peaks. The expanded insert highlights a more detailed view for each pathway showing significant enrichment (FDR<5%). Individual genes within each pathway are shown, coloured by the location of the strongest differential peak within that gene for that pathway, but also including the location of all differential peaks within each gene (peak distribution, below) and the number of peaks differential per gene (bottom). Note that this method of presentation also allows comparison of pathway overlap, showing whether 2 enriched pathways share many genes or represent independent enrichment.
While we maintain that this presentation concisely conveys a large amount of relevant information it may not have been clear that the result was a peak set enrichment and we have modified both the text and figure legend to improve interpretation (line 232-235 and Fig3 legend, lines 521-539).

Transcription factor activities can also be predicted from ATAC-seq data using transcription factor footprinting or motif enrichment analyses. In particular, it would be interesting to see if Myc activity was also predicted to be enhanced in the ATAC-seq data.
We thank the Reviewer for this interesting suggestion. We have now undertaken transcription factor footprint analysis of the ATAC-seq data against all TF PWMs in the JASPAR repository (n=519)[13]. We now include a Tornado plot showing enrichment of all TF motifs amongst differential peaks (Supplementary Fig S4G) along with an enrichment analysis of the top Transcription factors against gene sets from the Epigenomics RoadMap dataset. This confirms that the top enriched TF motifs are most commonly seen in primary CD8$^+$ memory T cells (Supplementary Fig S4H). The top TF motifs are included as a table (Supplementary Fig S4I) along with example footprint plots for one TF (CTCF) that is known to be a driver of CD8 T cell memory formation[14].
Myc is not itself one of the top differential TF motifs. However, the lack of Myc enrichment here, while interesting, does not preclude an effect of Myc in modulating glutaminolysis or mediating the Wnt-dominant epigenetic changes we observe. For example, Wnt is an upstream controller of Myc expression so chromatin remodelling of Wnt may alter Myc responsiveness indirectly without Myc itself undergoing chromatin remodelling. A key strength of our approach is the unsupervised identification of Myc and Wnt in different genomic datasets, highlighting links between them in the control of memory formation (ie we identified Myc and Wnt signalling through broad screens of enriched pathways rather than selecting them based on prior knowledge).
Further discussion of this point is also now included in the manuscript (lines 243-250).

6.The scatterplots shown for the inhibitor screening data were often difficult to discern conclusions (e.g., those in Fig. 1E–G). Please include the raw data about the impacts of each of the drugs, with names listed, on each of the parameters tested (IL7RA, PD-1, cell proliferation, CD25, and survival) to complement these figures.
Additional data is referenced in the Key Resources table with raw data accessible either through referenced repositories or in the Mendeley repository cited (https://data.mendeley.com/drafts/pncgztw2mg).

7.For the pathway enrichment and metabolite-set enrichment analyses, it was unclear as to whether all differentially expressed genes/metabolites were included or if they were separated into upregulated versus downregulated. Separating them would help better discern whether a given gene signature was increased or decreased in each comparison and aid with interpretations.

For the pathway and metabolite enrichment shown in Fig2C-F we intentionally considered enrichment of both up and downregulated genes considered together. Altered acetylation may result in either increase or decrease in activity of a protein depending on the residue that is altered and the protein in question[15]. Consequently, it is not safe to assume that more acetylation results in higher activity or vice versa. The definition of pathways tested can also include inhibitors or activators of that pathway. Reduced expression of an inhibitor could lead to activation or vice versa. Consequently, we chose to consider enrichment amongst all differentially acetylated peptides or metabolites rather than segregate by direction of effect.

It should also be noted that the majority of differentials were increased by treatment (for metabolites particularly) and consequently repeating the analysis on upregulated features gives comparable results while the downregulated features are underpowered to show any clear enrichment.
We have now included clarification of this approach in the methods (lines 986-988).

Minor comments

1.The readability of this manuscript could be improved via careful editing of the figure callouts, as many seemed to be incorrect (e.g. data for CD25 and cell survival were not shown in Fig. 1D, E) and/or disorganized in the text (e.g., panels in Fig. 4 were cited out of the more logical, sequential order).
We have checked the manuscript for incorrect callouts or annotations and amended those that are highlighted by all Reviewers.
Regarding the specific examples given here, Fig 1D, E do not show expression of CD25 or survival hence they are not annotated as such. These are instead shown in Figs 1I-K. We have also checked the citation order of the panels in Fig4 (on p11) and find these to be correct.

2.For Table S3, it would be helpful to more precisely identify the target of the inhibitors rather than calling them "molecules". In particular, the different inhibitors of KDACs should be clearly labeled.
This has been amended in Table S3.

3.The incorporation of diagrams to describe experimental systems was appreciated, although the authors should also add more experimental details to their figure legends. It was challenging, at times, to match the descriptions in the Methods with the experimental systems shown in each of the figure panels.
We are pleased that the schematic figures were helpful to aid interpretation. We have included further schematics (e.g. Supplementary Fig S3A) and have added additional experimental detail to Figure legends as suggested.

4.The labels for Figure 4H were not clear – please illustrate which plots designate IL-7R MFI (left plots) and which designate PD-1 MFI (right plots).
Thank you – this has now been amended to clarify.

Similarly, in addition to the figure legend, please provide a legend key to illustrate what the orange versus blue bars refer to for Fig. S2C.
This has now been added to FigS2C.

5.For the Seahorse data, it is unclear why relative values are shown and how the data were normalized. Please clarify this point and more ideally, show the raw values for such assays.
For real-time cellular metabolic analysis using Agilent Seahorse XF technology normalization is a standard requirement of data processing to allow interpretation (for example, see[16,17]). The cell quantity per well is a commonly accepted reference value for data normalization alongside normalization to baseline value to show relative change in flux over time between multiple replicate runs. From the referenced paper above:

"Normalization of XF assay results is critical for accurate and consistent data interpretation and comparison".

6.The authors should carefully revise conclusions about the impact of beta-catenin and Myc signatures in their model. The data provided in the paper are correlative only (page 9, lines 233–235). Similarly, GLUD1 inhibition in the in vivo models only partially rescued selective phenotypes, so those conclusions should be carefully reviewed and revised as needed (e.g., page 11, lines 271–274).
We have modified the conclusions as suggested (now lines 288-291).

Finally, the authors should not call PD-1 an exhaustion marker in this context (page 4, line 93), as PD-1 is also expressed by activated T cells.
We agree with the Reviewer and this has been amended to clarify (line 93).

Reviewer #3 (Remarks to the Author):
The study by Sowerby and colleagues describes the ability of KDAC inhibitors to promote T cell memory responses after vaccination. It offers interesting insights derived from cell line and mice experiments, identifies enhanced glutaminolysis as the responsible mechanism, and reports on the results of a human challenge study which investigated the effects of vaproat, a repurposable KDACi, after seasonal influenza vaccination. These data are interesting and add to the increasing body of literature investigating the connection between immunometabolomics and vaccine responses.

Some comments and questions:
- Line 165-166: Selective blockade of glutaminolysis using the GLUD1 inhibitor R162 (but not more proximal inhibitors, Supp Fig3A)..."
However, in Supp Fig3A. only DMKG is shown which is not an inhibitor but, as described in the next phrase, would enhance metabolic flux through the TCA cycle. Which proximal inhibitors were used and, in the authors' opinion, why did they not have the same effect as more distal blockers?
We thank the Reviewer for this point. The additional inhibitors were included in SuppFig3A-E. This has now been clearly referenced in the manuscript and discussed as in response to Reviewer 2 above also. Specifically:
Data describing the effect of proximal glutaminolysis inhibitors was presented in Supplementary Figure 3B-D. This included 3 inhibitors of glutaminolysis, selected for their action at different points along the pathway, along with evidence that enhancing alpha-ketoglutarate could directly reproduce the effect seen in the in vitro phenotypic screen.
Specifically,
FigS3B: DMKG supplementation: addition of the cell-permeable analog of alpha-ketoglutarate dimethyl-ketoglutarate (DMKG) reproduced the phenotypic effect seen with KDACi treatment. This is not an inhibitor but confirms that increasing anaplerosis could reproduce the effect seen in the screening assay.
FigS3C: R162 (GLUD1 inhibitor) significantly reverses the memory phenotype when added to valproate-treated and stimulated T cells in vitro.
FigS3D: BPTES (GLS inhibitor) fails to reverse the memory phenotype when added to valproate-treated and stimulated T cells in vitro.
FigS3E: inhibitor C968 fails to reverse the memory phenotype when added to valproate-treated and stimulated T cells in vitro.

Anaplerosis is not the only mechanism by which glutaminolysis may alter cellular function with contributions also made to reactive oxygen species production, biosynthetic pathways and control of amino acid pools[4,7]. Inhibition of more proximal steps in the pathway leave these downstream functions impaired along with anaplerosis. The fact that only distal inhibition of GLUD1 by R162 impacted on the observed phenotype indicates that anaplerosis was the likely mechanism being driven by glutaminolysis that was relevant for the phenotype in question. Note that our data are consistent with previous reports of distinct effects of glutaminolysis inhibition when effected at different points of the pathway[8].

To make these points clear we have included a schematic Figure (SuppFig3A) highlighting the points of inhibition and additional discussion in the text (lines 170-179).

- Transcriptomics: The DEG analysis is only described in a cursory fashion in the methods section and should be described in more detail there. Were low-abundance genes removed prior to the DEG analysis? If so, how many were excluded? The two conditions to be compared were not independent, as the same cell line was used in two different states (with and without treatment, i.e., a paired design). How was this accounted for in the EdgeR workflow? Additionally, only reporting the number of DEGs does not convey much information to the reader. What kind of genes were up- and down-regulated? A functional annotation using gene ontology or alike would be helpful.

We thank the Reviewer for highlighting this point and agree that insufficient detail was provided on the DEG workflow used. The DEG analysis was undertaken in primary human CD8+ T cells. Prior feature filtration was undertaken using an expression threshold of cpm>1 in at least 6 samples (to allow expression in either treatment group) with a feature count of n=16,402. We used a contrast matrix with a pairwise design in limma[18] using an additive model matrix, considering it as a special case of blocking with block size of n=2 (as specified in limma: https://www.bioconductor.org/packages/devel/bioc/vignettes/limma/inst/doc/usersguide.pdf). Further details of the method used have been added to the Methods section to make this clear (lines 1021-1026).
We agree that reporting the number of differentially expressed genes is limited. That is why we continued directly to analyse the transcriptomic data more extensively using matrix factorization. This is outlined in more detail in response to the query below and includes extensive enrichment analysis representing substantial functional annotation.

- Figure: 3: Figure 3A is referenced in connection to differentially expressed genes (line 191) but does not contain anything on DEGs but is a schematics on MOFA. Confusingly, Figure 3 therefore jumps from MOFA (A) to DEGs (B) back to MOFA (C). In B) in the lower panel, it is very confusing to color genes in red/blue which according to the figure legend indicate statistical significance, even when the genes are clearly below the significance threshold. If the same genes that are significant in the upper panel should be labelled, a different color should be used.
However, I am not really convinced that this adds any useful information because clearly, not a single gene is differentially expressed in the lower panel; therefore all significant genes from the upper panel must be below the threshold and insignificant in the lower panel.
We thank the Reviewer for highlighting this point. To clarify the presentation we have altered the sequence of the Figures to make clear the sequential points being made: firstly that the set of differentially expressed genes (rather than just a small number of surface markers) was reversed on glutaminolysis inhibition (now Fig3A) and secondly (Fig3B onwards), that a more detailed functional annotation of gene expression data shows a treatment specific latent signature that is enriched for T cell memory profiles (Fig 3B onwards) that were used for the original in silico screen.

For the differentially expressed genes in Fig 3A, we coloured the same genes in the upper panel and lower panel to make illustrate how the differentially expressed genes were affected by inhibition of glutaminolysis. We have now altered the second panel to remove this annotation to avoid confusion.

- "MOFA was used "to interpret these gene expression changes" (line 193). This could also rather easily be done with e.g. GO annotation or BTMs etc. Why did the authors choose to use MOFA and what kind of insights does this more complex approach offer that e.g. a GO or BTMs/BloodGen3Modules would not provide? As far as I know, the usual application and strength of MOFA is to combine different –omics datasets for the joint analysis of multiple datasets. As many datasets are available in this study, have the authors considered performing this kind of integrative analysis?
We agree that GO annotation of differentially expressed genes is one approach to functional annotation of gene expression data. We preferred to use matrix factorization (MOFA) for the following reasons. MOFA is a matrix factorization method used to identify a lower dimensional representation of the sample space, i.e. an unsupervised method of latent signature discovery. It is most commonly applied to integrate multiple datasets captured on a common sample set (rather than the multi-omic data across different experiments captured here). MOFA is an established method for unsupervised discovery of latent programmes of gene expression and is applicable when there is a single dataset rather than multiple for integration[19,20]. Matrix factorisation also identifies all latent signatures in the data, rather than restricting analysis of a list of differentially expressed genes for a single subgroup comparison. More importantly, it is an interpretable method that allows interrogation of a ranked distribution of contributory feature weights against external repositories of gene sets. As it identifies the weighted contribution of each feature (gene) to each factor, there is no requirement thresholding at a given significance cutoff, as is the case with list-based enrichment. We also note that GO annotation provides more limited functional annotation compared to the hallmark sets and immunological experimental signatures from the ImmuneSigDB collections used here. We were thefore able to iteratively test for enrichment of multiple gene sets against each factor's feature weight distribution. Coexpression network modelling (e.g. WGCNA) is an alternative approach, although this defines discrete, non-overlapping modules of genes based on degree of coexpression. Consequently, genes can only belong in a single module. Such a discrete approach assumes that all genes contribute to only a single process. With factorization, all genes contribute (variably) to all factors, more consistent with known biological pathway overlaps.
The approach we have taken therefore allows for unsupervised discovery of latent gene expression programmes in the data, their association with treatment and interpretation through iterative enrichment of external gene sets, as the Reviewer suggests.

To ensure comparability of our approach with the list enrichment approach we have also undertaken enrichment of the top differentially expressed genes (Valp vs vehicle, FDR<0.05) against the hallmark sets and find that only two sets (Myc targets and Oxidative phosphorylation as presented in Fig 3D) are significantly enriched in this list.

- Human challenge study: The study protocol specifies that the aim was to recruit 180 participant based on a given power calculation. However, in the paper, only 69

participants were recruited to the study, less then half. Why? How would this impact the results of the study? This should be acknowledged in the paper.
Thank you for highlighting this apparent inconsistency. Target recruitment was based on power calculations that necessarily modelled an estimated effect size in humans based on the results available from both *in vitro* and *in vivo* studies.
The MEMRI study protocol (Section 7.1) indicates a target enrolment of "up to 180 participants in order to achieve 50 participants from each vaccination group completing the study (assuming a 20% dropout rate)". The protocol also provides detailed estimates of statistical power based on observed effect sizes in our preclinical studies, and variance in observed influenza vaccine studies in humans (referenced in detail in Protocol Section 7.1 and provided in methods lines 910-920). This resulted in an estimated need to recruit group sizes of n=25-36 (for cellular and humoral outcomes respectively). We obtained ethical approval for "up to 180" participants to allow subgroup analysis by drug dose and other covariates (sex, prior response etc).
As recruitment was undertaken during the period of a COVID pandemic, this was slower than anticipated and recruitment of those >65 years old were precluded by our BioResource SAB (see below). Consequently, the study recruited n=69 individuals, with adequate power to analyse against treatment/control groups but not for subgroup analyses. They study was therefore adequately powered and recruited to target for the analyses presented.
Although the study was not a formal clinical trial, it was approved as an Experimental Medicine study by the MHRA and we have included the full protocol for transparency of reporting, including a priori power calculations and the assumptions and data on which they were based.
We have now clarified this additional information in the methods section to clarify for readers (lines 910-920 and lines 956-967, methods).

- How are the baseline characteristics distributed across the randomized groups? Figure S7 (not S6 as written in line 847) only shows figures for age and sex. These, however, were the two variables included in the minimization algorithm and it is therefore expected that these are balanced (assuming that the minimization algorithm worked correctly). According to the protocol, several other characteristics that are associated with vaccine responses were also captured for each participant, such as BMI, smoking, and prior vaccination history. A detailed baseline characteristics table is required comparing the distributions of these relevant variables including the pre-vaccination titers/ T cell frequencies across study groups.

We thank the Reviewer for highlighting this. We have corrected the citation within the text and updated Supplementary Figure S7B to include a table comparing characteristics of individuals within each group.
Prior exposure to influenza infection or vaccination is handled through normalization to baseline antibody titer or T cell frequency (to show relative expansion). Further detail is provided in response to the query below regarding ratios, where both raw data and normalized ratios are provided for each subject included (Supplementary Fig S8A-E).

- Two different drug dosages (100mg and 200mg valproate) were used in the study and according to the study protocol, they were to be analysed separately. In the paper, without explanation, these are pooled together. Why? Dose-dependent relationships would give more information about the results and/or provide insights if no such relationship is seen. Accordingly, in Figure 6 C, D, E etc. two different colors should be used for the two dosages.

We thank the Reviewer for highlighting this point. Selecting an optimal dose for use in a human study is challenging and more so when the effective dose *in vitro* is substantially below that used in clinical practice. We included two doses to gain additional insight into dose-response relationship as the Reviewer suggests and have now included a breakdown of the observed outcome by dose group (Supplementary Figure S9). As indicated above, the study was ultimately underpowered to allow analysis by dose so the two groups were considered together for the main analysis as pre-specified in the protocol. This has been clarified in the methods section (956-967).

- The study protocol states in the analysis plan, that linear and logistic regression models will be used to explore the association between study endpoints and variables including age, drug dose, and prior memory responses. This has so far not really been addressed yet but would be important. Additionally, on multiple occasions the study protocol mentions age stratification into 18-65yo and >65 yo but this has not been explored in the paper.

The protocol was adapted due to the COVID pandemic as IRB review by the NIHR BioResource Advisory Board precluded recruitment of those >65years old due to concerns over potential increased risk of infectious exposure on attending the hospital site for vaccination. This has now been included as an explanatory note in the methods section (lines 956-967).

The study was also underpowered for regression analyses by dose and additional covariates. However, we agree with the Reviewer that this is important information and was pre-specified in the protocol. We have therefore undertaken additional multivariate logistic regression analysis including potentially confounding covariates and present this analysis in Supplementary Fig S8F.

Prior responses were taken into account through normalization for baseline/memory response with age and sex as minimization variables as indicated. Supplementary Figure S7B also now shows additional baseline characteristics compared between experimental groups including BMI, smoking status, blood pressure and ethnicity.

- Only ratios are displayed in Figure 6 A, D, E. How would absolute values look like? An ANCOVA analysis with adjustment for prior titer/cell frequency levels would be less prone to regression to the mean effects while still be able to adjust for baseline levels.

We agree with the Reviewer's suggestion that full raw data is helpful in full interpreting the results presented. For the primary analysis data were presented as normalized values (Fig6A-F) to account for prior exposure to infection or vaccination, showing relative expansion compared to baseline. To address this point, in the submitted manuscript we had also presented raw data for each of the outcomes, including influenza-specific T cell count (Supplementary Fig S8A) and frequency (Supplementary

Figure S8B) and antibody titre (Supplementary Fig S8C-E) at each timepoint post-vaccination (d0, d7 and d30).

- Have adverse effects been recorded? The dose was low and the study was not registered as a clinical trial, but valproate does have serious potential side effects and it would be important to include these.

We thank the Reviewer for highlighting the importance of safety and toxicity. The study was formally reviewed by MHRA and considered an experimental medicine study rather than a CTIMP (methods, lines 884-886). However, prospective collection of safety data was included in the protocol. While valproate has serious potential side effects these largely relate to potential teratogenicity. All individuals of child-bearing potential in the study were obliged to have a negative pregnancy test prior to enrolment (Protocol, p12). Side effects are also dose dependent and we used doses of around 10% of the clinical dose used as an anticonvulsant, hence adverse effects were not expected and were not seen. This has now been clarified in the text (line 326) and in Supplementary Fig S7B which includes data on both serious (none seen) and non-serious adverse event rates (which are also not different between treatment and control groups).

Note that we have also now incorporated formal logistic regression modelling of outcomes against additional covariates including minor adverse events (Supplementary Fig S8F).

- A key resources is mentioned on multiple occasions in the paper, e.g., for GEO accessions numbers etc. However, I could not find this table.

A key resources table including Reagents, samples, chemicals, proteins, commercial assays, deposited data (including links to repositories), software and algorithms is included (https://data.mendeley.com/drafts/pncgztw2mg).

- Abbreviation "ob" / "bd" in Figure S7 should be explained in the legend. Preferably, it is just avoided, there would be enough space to write it out like 1x/day and 2x/day.

This has been amended as suggested by the Reviewer to read ‘once daily’ or ‘twice daily’ (Supplementary Fig S7A).

Response References:

1. Le, A., *et al.* Glucose-independent glutamine metabolism via TCA cycling for proliferation and survival in B cells. *Cell Metab* **15**, 110-121 (2012).
2. Boothby, M. & Rickert, R.C. Metabolic Regulation of the Immune Humoral Response. *Immunity* **46**, 743-755 (2017).
3. Yoo, H.C., Yu, Y.C., Sung, Y. & Han, J.M. Glutamine reliance in cell metabolism. *Exp Mol Med* **52**, 1496-1516 (2020).
4. Yang, L., Venneti, S. & Nagrath, D. Glutaminolysis: A Hallmark of Cancer Metabolism. *Annu Rev Biomed Eng* **19**, 163-194 (2017).
5. Feng, J., *et al.* Antibody quantity versus quality after influenza vaccination. *Vaccine* **27**, 6358-6362 (2009).
6. Sanz-Munoz, I., *et al.* Long-term influenza antibodies profiles in previously vaccinated and non-vaccinated adults. *Hum Vaccin Immunother* **19**, 2236537 (2023).

7. Altman, B.J., Stine, Z.E. & Dang, C.V. From Krebs to clinic: glutamine metabolism to cancer therapy. *Nat Rev Cancer* **16**, 773 (2016).
8. Madden, M.Z.*, et al.* Differential Effects of Glutamine Inhibition Strategies on Antitumor CD8 T Cells. *J Immunol* **211**, 563-575 (2023).
9. Carr, E.L.*, et al.* Glutamine uptake and metabolism are coordinately regulated by ERK/MAPK during T lymphocyte activation. *J Immunol* **185**, 1037-1044 (2010).
10. Colombo, S.L.*, et al.* Molecular basis for the differential use of glucose and glutamine in cell proliferation as revealed by synchronized HeLa cells. *Proc Natl Acad Sci U S A* **108**, 21069-21074 (2011).
11. Thangavelu, K., Chong, Q.Y., Low, B.C. & Sivaraman, J. Structural basis for the active site inhibition mechanism of human kidney-type glutaminase (KGA). *Sci Rep* **4**, 3827 (2014).
12. Wang, J.B.*, et al.* Targeting mitochondrial glutaminase activity inhibits oncogenic transformation. *Cancer Cell* **18**, 207-219 (2010).
13. Rauluseviciute, I.*, et al.* JASPAR 2024: 20th anniversary of the open-access database of transcription factor binding profiles. *Nucleic Acids Res* **52**, D174-D182 (2024).
14. Zhu, S.*, et al.* Antigen exposure reshapes chromatin architecture in central memory CD8(+) T cells and imprints enhanced recall capacity. *Proc Natl Acad Sci U S A* **120**, e2313476120 (2023).
15. Drazic, A., Myklebust, L.M., Ree, R. & Arnesen, T. The world of protein acetylation. *Biochim Biophys Acta* **1864**, 1372-1401 (2016).
16. Yoo, I., Ahn, I., Lee, J. & Lee, N. Extracellular flux assay (Seahorse assay): Diverse applications in metabolic research across biological disciplines. *Mol Cells* **47**, 100095 (2024).
17. Janssen, J.J.E.*, et al.* Novel standardized method for extracellular flux analysis of oxidative and glycolytic metabolism in peripheral blood mononuclear cells. *Sci Rep* **11**, 1662 (2021).
18. Ritchie, M.E.*, et al.* limma powers differential expression analyses for RNA-sequencing and microarray studies. *Nucleic Acids Res* **43**, e47 (2015).
19. Argelaguet, R.*, et al.* Multi-omics profiling of mouse gastrulation at single-cell resolution. *Nature* **576**, 487-491 (2019).
20. Argelaguet, R.*, et al.* Multi-Omics Factor Analysis-a framework for unsupervised integration of multi-omics data sets. *Mol Syst Biol* **14**, e8124 (2018).

REVIEWER COMMENTS
[responses highlighted in red; line references to amended manuscript version; updated text in amended manuscript version also highlighted in red]

Reviewer #1 (Remarks to the Author):
The authors have satisfactorily addressed the comments and poised raised by this reviewer.

We thank the Reviewer for their time and comments which we feel have improved the manuscript.

Reviewer #2 (Remarks to the Author):
The authors performed new analyses and/or clarified points in response to my original comments. The study will be an important advance in understanding mechanisms to boost memory immune responses and the contribution of metabolic rewiring to such a
process.

We thank the Reviewer for their time and comments in improving this work.

A few minor comments remain to be addressed:

1. While the functional impacts of valproate treatment in improving secondary recall responses and vaccine responses are striking, the original question remains about the type(s) of memory T cell responses that are being generated in response to valproate treatment (Tcm, Tem, or Trm). The analyses of the cell populations at day 30 before re-challenge in the IAV infection model would help to clarify the points on lines 276–279 regarding whether those cells that are expanded are tissue-resident or circulating (Tcm or Tem) populations. This reviewer feels that such analyses would still be a beneficial addition to this paper, given the differential effects of distinct memory T cell populations on long-term immunity in tissues. At minimum, this point should be acknowledged as a limitation of this study, especially in the human patient cohort.

We thank the Reviewer for highlighting this. We show an absolute expansion of NP and PA-specific CD8T cells in lung d30pi which is suggestive of Trm (Supp Fig 3D-F). However, while we cannot exclude contamination from expansion in circulation, the pattern of IHC staining (Fig4O, P) is consistent with a resident population rather than a vascular source. We agree that differentiating between tissue resident and circulating populations would further add to the work. Although this is not feasible in human studies, we have now acknowledged this limitation in the manuscript as suggested (lines 384-6).

2. Based on the authors' response to the original comment #4, please revise the names in Fig. 3E to match exactly what is shown in MSigDB, especially to reflect the use of DN or UP at the end of the signature. For example, it is presumed, based on the authors'
response, that the Day15_Eff_vs_Memory_CD8_Tcell signature is the Kaech_Day15_Eff_vs_Memory_CD8_Tcell_DN signature, which is a memory T cell signature. Without this additional information, this reviewer interprets the signature to show genes enriched in effector cells rather than memory cells. The same applies to all the other signatures listed.

This was provided in full in Supp Table 6 but the full details of each signature were not included in figure itself. Given the length of each signature's full description it is not feasible to include it in the Figure itself. However, the table has been updated to reflect each signature's full name along with the associated link to extended information in MSigDB. Both the Figure (Fig3E) and the table (Supp Table 6) show concordant changes across multiple signatures from different studies: for example, valproate treated cells show enrichment of Day15_Eff_vs_Memory_CD8_Tcell_DN

(up in memory vs effector), GSE41867_LCMV_ARMSTRONG_VS_CLONE13_DAY15_EFFECTOR_CD8_TCELL_UP (up in non-exhausted memory vs exhausted) and GSE9650_EXHAUSTED_VS_MEMORY_CD8_TCELL_DN (up in memory vs exhausted).

3. The label is missing above the bubbles in Fig. 3D.

Thank you. This has been corrected.

4. For the new experimental schematic in Fig. S3A, the authors should revise to GLUD1 rather than its alternative name of GDH1. This will aid the readers of the manuscript, since GLUD1 is the name used throughout the text.

5. Please add a statement to the methods regarding how the Seahorse data were normalized (as described in the response to minor comment #5). This point remains unclear in the revised manuscript.

Thank you and we are pleased the response was helpful. This has now been included in the methods section (lines 1029 -1034).

6. In the discussion (and related to the authors' response to comment #1), the authors could clarify that glutamine and glutaminolysis have additional effects beyond T cell activation, citing those papers mentioned in the original comment #2 (Tang et al. 2023 Nat Immunol; Nabe et al. 2018 Cancer Sci; Huang et al. 2021 Cell; Xu et al. 2023 Cell Rep), as well as the notion that glutamine deprivation or glutaminase inhibition can have differential effects on CD4 and CD8 T cell populations (Nakaya et al. 2014 Immunity; Johnson et al. 2018 Cell). This provides broader context to the readers about the overall impact of altering glutamine metabolism on T cell responses, especially in consideration of the newly included data on valproate treatment alone in the IAV model.

We thank the Reviewer for this suggestion. We agree that this provides additional helpful context for the reader and have included further discussion as suggested along with the proposed references (lines 180 – 186).

1

Reviewer #3 (Remarks to the Author):
My comments have been adequately addressed. In my opinion, this work is now suitable for publication.

We thank the Reviewer for their time and comments which we feel have improved the manuscript.

Response References:

1. Johnson, M.O., *et al.* Distinct Regulation of Th17 and Th1 Cell Differentiation by Glutaminase-Dependent Metabolism. *Cell* **175**, 1780-1795 e1719 (2018).
