# Supplementary material for: Multiomic analysis identifies glutaminolysis-dependent metabolic enhancement of immune memory utilised for vaccine development
Source: Nat Commun. 2026 Jun 26;17:8018. doi: 10.1038/s41467-026-74866-4 (PMC13454449; doi:10.1038/s41467-026-74866-4)
Supplement: Supplementary file 2 [file 41467_2026_74866_MOESM1_ESM.pdf]

Multiomic analysis identifies glutaminolysis-dependent metabolic enhancement of immune memory utilised for vaccine development

Sowerby JM[#1, 2], Kotagiri P[#1, 2], Clare S[1,2,3], Griffiths D[1, 2], Knight O[1,2], Cuthbertson I[1,2], Harcourt K[3], Pereyra Gerber P[1,2,] Pshenichnaya I[1], Connor C[1,2], Denton A[1,2], Stoll, GA[1,4], Matara C[2], Keel, C[5], Manferrari G[1,2], Birkle T[1,2], Naydenova P[1,2], Peck H[6], Lyons PA[1,2], Barr I[6], Modis Y[1,4], Dougan G[1,2], Matheson NJ[1,2,7], Smith KGC[1,2], McKinney EF[1,2,8*.]

# these authors contributed equally to this work.
1 Cambridge Institute of Therapeutic Immunology and Infectious Disease, Jeffrey Cheah Biomedical Centre, Cambridge, U.K.
2. Department of Medicine, University of Cambridge School of Clinical Medicine, Cambridge, U.K.
3. Wellcome Sanger Institute, Wellcome Genome Campus, Hinxton, Cambridge, CB10 1SA
4. Molecular Immunity Unit, Department of Medicine, University of Cambridge, MRC Laboratory of Molecular Biology, Francis Crick Avenue, Cambridge, CB2 0QH, UK.
5. National Institute for Health Research Cambridge Centre for Clinical Research, Cambridge, U.K.
6. WHO Collaborating Centre for Reference and Research on Influenza (VIDRL), Peter Doherty Institute for Infection and Immunity, 792 Elizabeth Street, Melbourne, VIC3000, Australia.
7. NHS Blood and Transplant, Cambridge, UK..
8. Cambridge Centre for Artificial Intelligence in Medicine, Cambridge, U.K.
*corresponding author
Correspondence: efm30@cam.ac.uk

Supplementary Information

Supplementary Figures 1-10
Supplementary Table 1 (Key Resources Table)

Figure S1

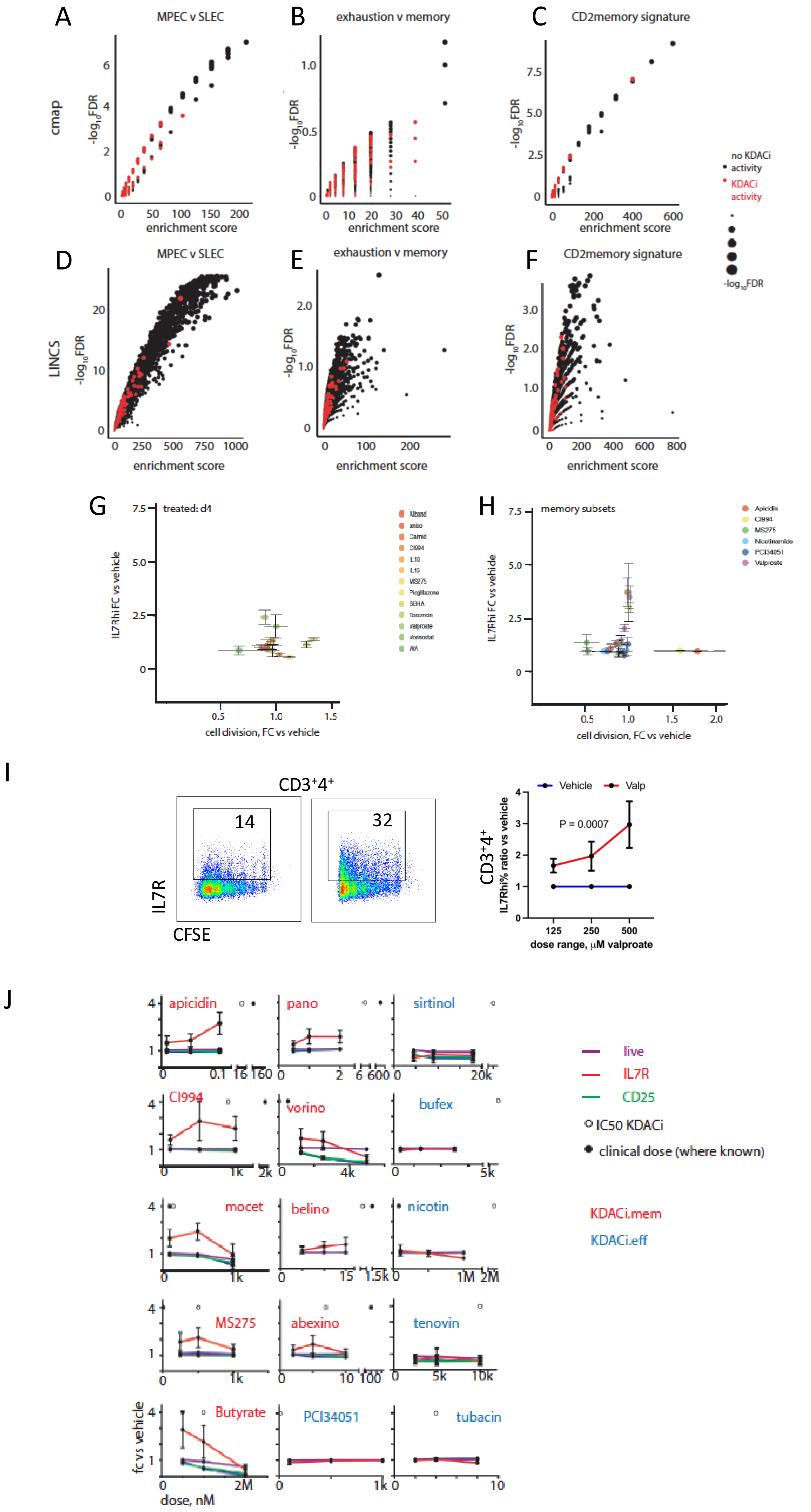
A
MPEC v SLEC
B
exhaustion v memory
C
CD2memory signature
cmap
-log10FDR
enrichment score
no KDACi activity
KDACi activity
D
E
F
LINCS
G
treated: d4
IL7Rhi FC vs vehicle
cell division, FC vs vehicle
H
memory subsets
Apicidin
CI994
MS275
Nicotinamide
PCI34051
Valproate
I
CD3+4+
14
32
IL7R
CFSE
Vehicle
Valp
P = 0.0007
CD3+4+ IL7Rhi% ratio vs vehicle
dose range, µM valproate
J
apicidin
pano
sirtinol
CI994
vorino
bufex
mocet
belino
nicotin
MS275
abexino
tenovin
Butyrate
PCI34051
tubacin
fc vs vehicle
dose, nM
live
IL7R
CD25
IC50 KDACi
clinical dose (where known)
KDACi.mem
KDACi.eff

Figure S1. Extended in silico and in vitro compound screening

Scatterplots (enrichment score vs $-\log_{10}FDR$) showing enrichment of T cell memory signatures (Supp Data 1) against connectivity map (A-C, n= 6100) and LINCS L1000 small molecule response signatures (D-F, LINCS_L1000_Chem_Pert_up/down, n=33132). (G) Scatter plot showing maximum change in $IL7R^{hi}$ MPEC (y-axis, fold change v vehicle) and cell division (x-axis, fc v vehicle) for each of 42 selected treatments (n=844, mean +/-sem) as shown in Fig1D, coloured by individual compounds (Supp Data 2). (G) Scatter plot showing maximum change in $IL7R^{hi}$ MPEC (y-axis, fold change v vehicle) and cell division (x-axis, fc v vehicle) for each of 13 selected treatments (n=67, mean +/-sem) for cells treated on d4 post stimulation (as shown in Fig1F). (H) Scatter plot showing maximum change in $IL7R^{hi}$ MPEC (y-axis, fold change v vehicle) and cell division (x-axis, fc v vehicle) for each of 6 selected KDACi (n=102, mean +/-sem) as shown in Fig1G, coloured by individual compounds. Full compound details given in Supp Data 2. Numbers reported are the total number of measurements contributing to each plot. (I) Representative scatterplot (left) and summary line/scatterplot (right) showing dose-dependent MPEC ($IL7R^{hi}cfse^{lo}$) phenotype differentiation of $CD3^{+}4^{+}$ T cells on valproate treatment during polyclonal stimulation with anti-CD2/3/28 *in vitro*. (J) Line and scatterplots showing dose dependent (x-axis) impact of KDACi treatment (y-axis) on $CD8^{+}$ T cell phenotype (y axis). red = KDACi.mem, blue = KDACi.eff. Open circle = IC_{50} KDACi effect, closed circle = clinically approved dose where available (Supp Data 3).

Figure S2

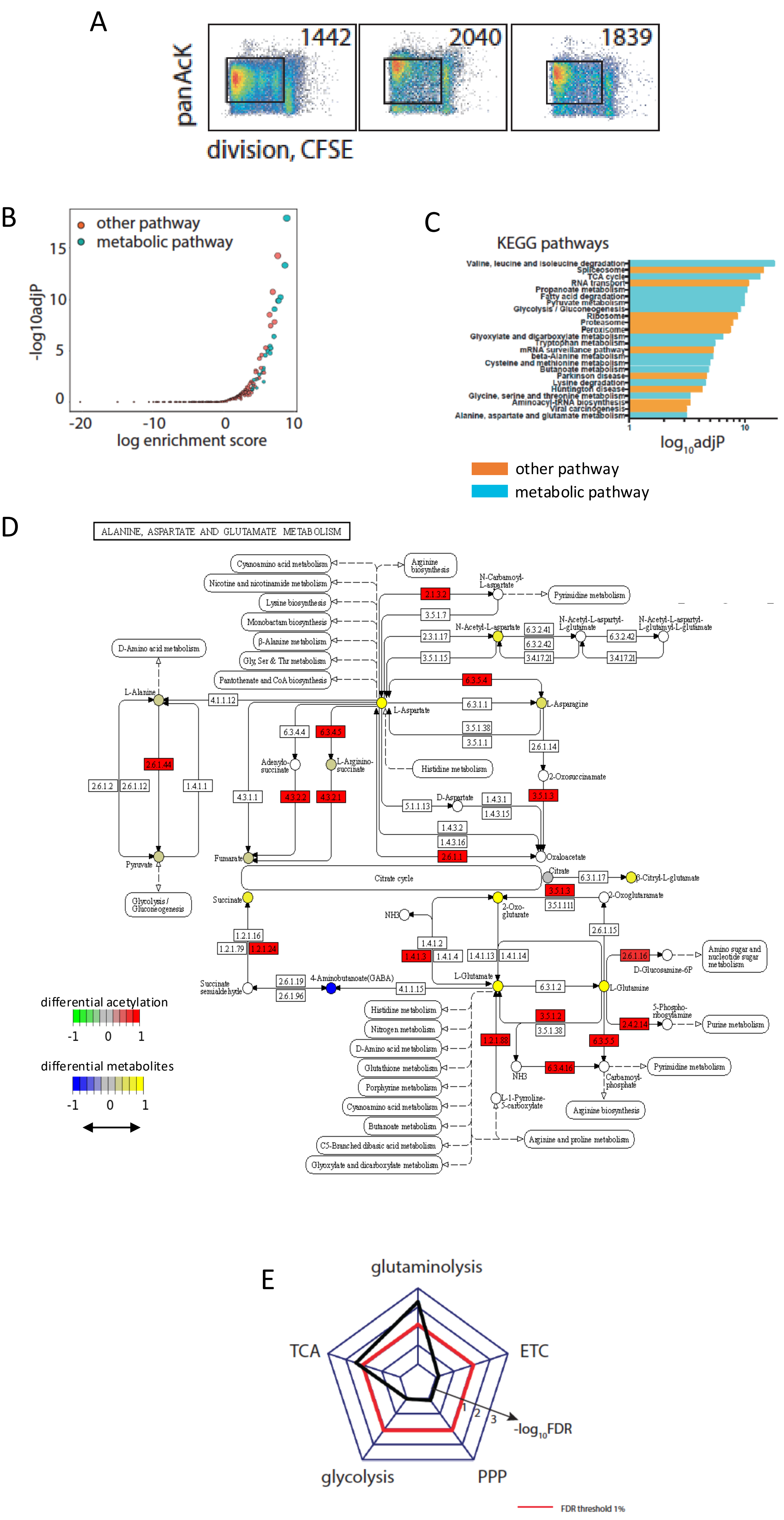
A
1442
2040
1839
panAcK
division, CFSE
B
other pathway
metabolic pathway
-log10adjP
log enrichment score
C
KEGG pathways
Valine, leucine and isoleucine degradation
Spliceosome
TCA cycle
RNA transport
Propanoate metabolism
Fatty acid degradation
Pyruvate metabolism
Glycolysis / Gluconeogenesis
Ribosome
Proteasome
Peroxisome
Glyoxylate and dicarboxylate metabolism
Tryptophan metabolism
mRNA surveillance pathway
beta-Alanine metabolism
Cysteine and methionine metabolism
Butanoate metabolism
Parkinson disease
Lysine degradation
Huntington disease
Glycine, serine and threonine metabolism
Aminoacyl-tRNA biosynthesis
Viral carcinogenesis
Alanine, aspartate and glutamate metabolism
log10adjP
other pathway
metabolic pathway
D
ALANINE, ASPARTATE AND GLUTAMATE METABOLISM
Cyanoamino acid metabolism
Nicotine and nicotinamide metabolism
Lysine biosynthesis
Monobactam biosynthesis
β-Alanine metabolism
Gly, Ser & Thr metabolism
Pantothenate and CoA biosynthesis
Arginine biosynthesis
N-Carbamoyl-L-aspartate
Pyrimidine metabolism
N-Acetyl-L-aspartate
N-Acetyl-L-aspartyl-L-glutamate
N-Acetyl-L-aspartyl-L-glutamyl-L-glutamate
D-Amino acid metabolism
L-Alanine
L-Aspartate
L-Asparagine
Adenylo-succinate
L-Arginino-succinate
Histidine metabolism
2-Oxosuccinamate
D-Aspartate
Pyruvate
Fumarate
Oxaloacetate
Glycolysis / Gluconeogenesis
Citrate cycle
Citrate
β-Citryl-L-glutamate
Succinate
2-Oxo-glutarate
2-Oxoglutaramate
NH3
Amino sugar and nucleotide sugar metabolism
D-Glucosamine-6P
Succinate semialdehyde
4-Aminobutanoate(GABA)
L-Glutamate
L-Glutamine
5-Phospho-ribosylamine
Purine metabolism
Histidine metabolism
Nitrogen metabolism
D-Amino acid metabolism
Glutathione metabolism
Porphyrine metabolism
Cyanoamino acid metabolism
Butanoate metabolism
C5-Branched dibasic acid metabolism
Glyoxylate and dicarboxylate metabolism
Carbamoyl-phosphate
Pyrimidine metabolism
L-1-Pyrroline-5-carboxylate
Arginine biosynthesis
Arginine and proline metabolism
differential acetylation
differential metabolites
E
glutaminolysis
TCA
ETC
glycolysis
PPP
-log10FDR
FDR threshold 1%

Figure S2. Extended enrichment analysis of acetylomic and metabolomic data
(A) Representative scatterplots illustrating total cellular acetyl-lysine (panAcK) quantification by cell division (CFSE dilution, x-axis) for selected KDACi (B) Bubble plot and (C) bar plot showing enrichment of metabolic (blue) and other (orange) pathways amongst differentially acetylated proteins following treatment of HEK cells with KDACi.mem and KDACi.eff compounds, as indicated in Fig2A. (D) Alanine, Aspartate and Glutamate metabolic pathway (KEGG 002050) showing differentially acetylated proteins (red) and differential metabolites (yellow) on KDACi.mem treatment as in Fig 2A-E. (E) Radar plot illustrating enrichment of principal metabolic pathways amongst ranked metabolites differentially induced by valproate/apicidin treatment of stimulated primary $CD8^+$ T cells (as in Fig2E). Threshold FDR<0.05 in red, radial distance = $-\log_{10}$FDR. PPP = pentose phosphate pathway, TCA = tricarboxylic acid, ETC = electron transport chain.

Figure S3

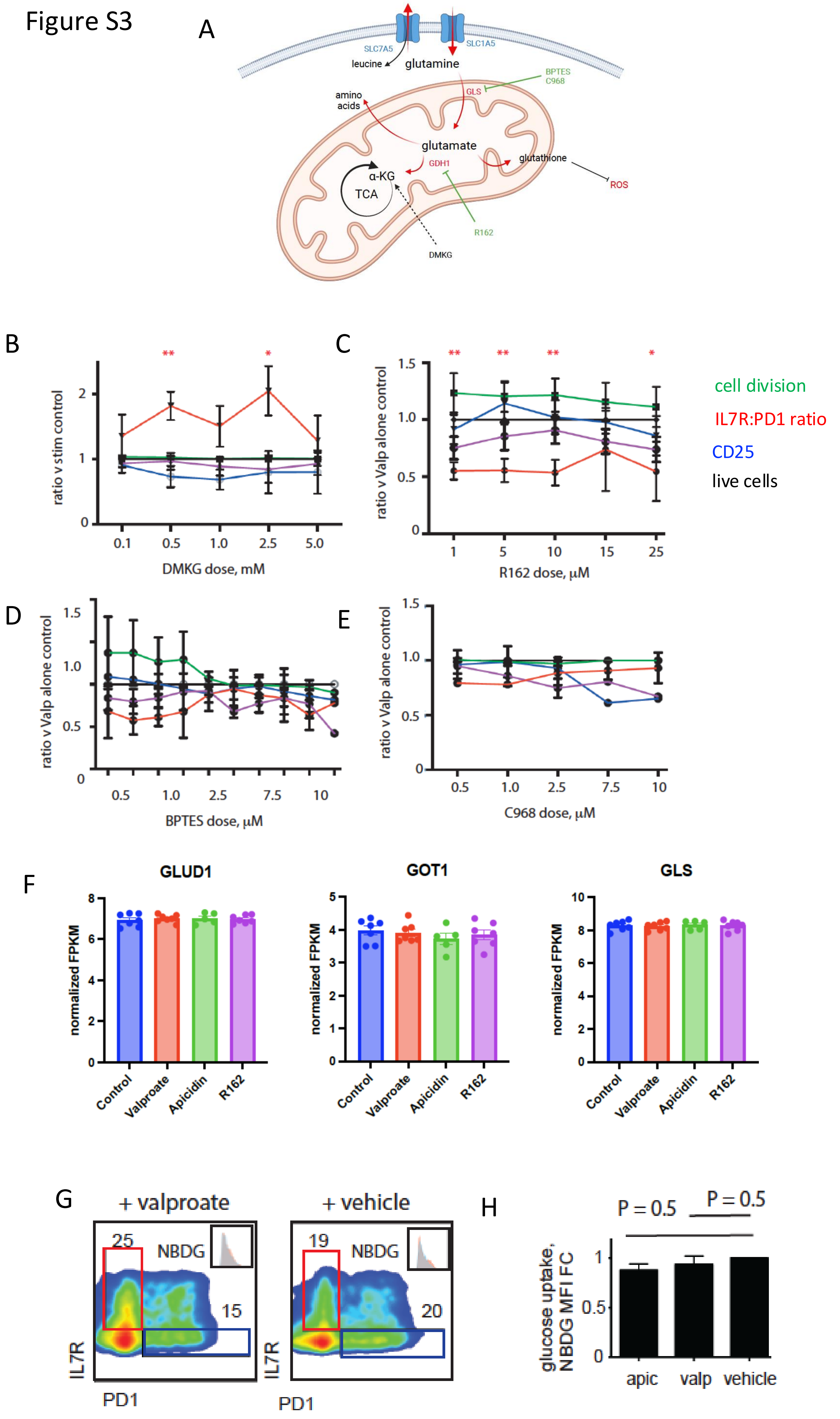

A
SLC7A5
SLC1A5
leucine
glutamine
BPTES
C968
GLS
amino acids
glutamate
GDH1
glutathione
ROS
α-KG
TCA
R162
DMKG
B
ratio v stim control
DMKG dose, mM
0.1
0.5
1.0
2.5
5.0
C
ratio v Valp alone control
R162 dose, µM
1
5
10
15
25
cell division
IL7R:PD1 ratio
CD25
live cells
D
ratio v Valp alone control
BPTES dose, µM
0.5
1.0
2.5
7.5
10
E
ratio v Valp alone control
C968 dose, µM
0.5
1.0
2.5
7.5
10
F
GLUD1
GOT1
GLS
normalized FPKM
Control
Valproate
Apicidin
R162
G
+ valproate
+ vehicle
25
15
19
20
NBDG
IL7R
PD1
H
P = 0.5
P = 0.5
glucose uptake, NBDG MFI FC
apic
valp
vehicle

Figure S3. Extended in vitro metabolic analysis

(A) Schematic illustration of the glutaminolysis pathway and the action of inhibitors and mimics used. (B-E) Line and scatterplots showing effect (fold change vs vehicle control) of (B) the cell-permeable αKG donor dimethyl-ketoglutarate (DMKG) and (C-E) the glutaminolysis pathway inhibitors R162 (C), BPTES (D) and C968 (E). $CD8^+$ T cell phenotype was measured d6 after polyclonal stimulation with anti-CD3/28 bead. Green = cell division, red = IL7R:PD1 ratio, blue = CD25, purple = live $CD8^+$ T cell % of total viable events. (F) Barplots illustrating expression of glutaminolysis pathway enzymes in primary CD8+_T cells treated with each compound as in Fig3. All comparisons non-significant ($p>0.05$). (G) Representative scatterplots (G) and summary bar plot (H, mean +/- sem, n=4) showing fluoroglucose uptake (NBDG, inset histograms) by MPEC phenotype ($IL7R^{hi}PD1^{lo}$) and valproate or vehicle treatment.

Figure S4

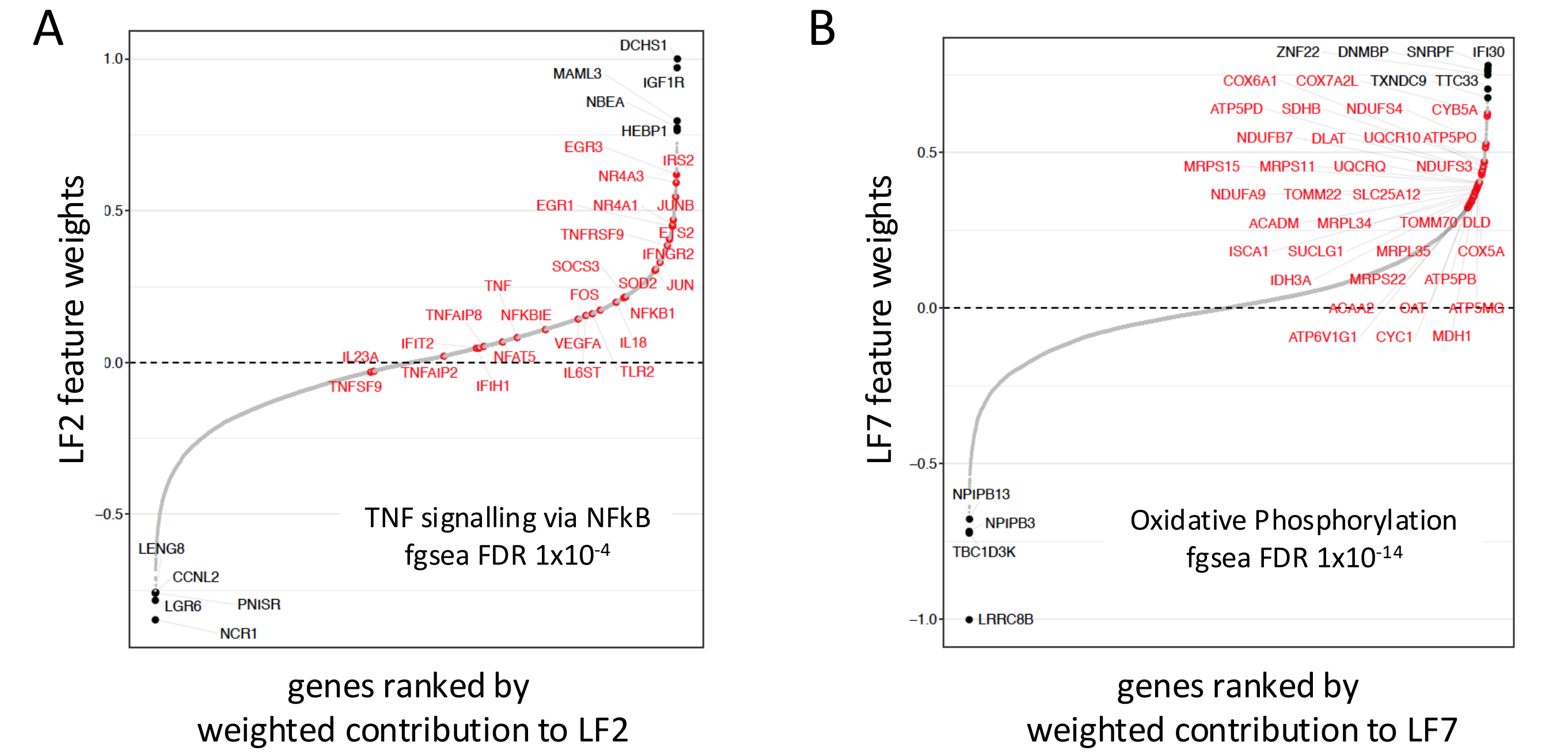

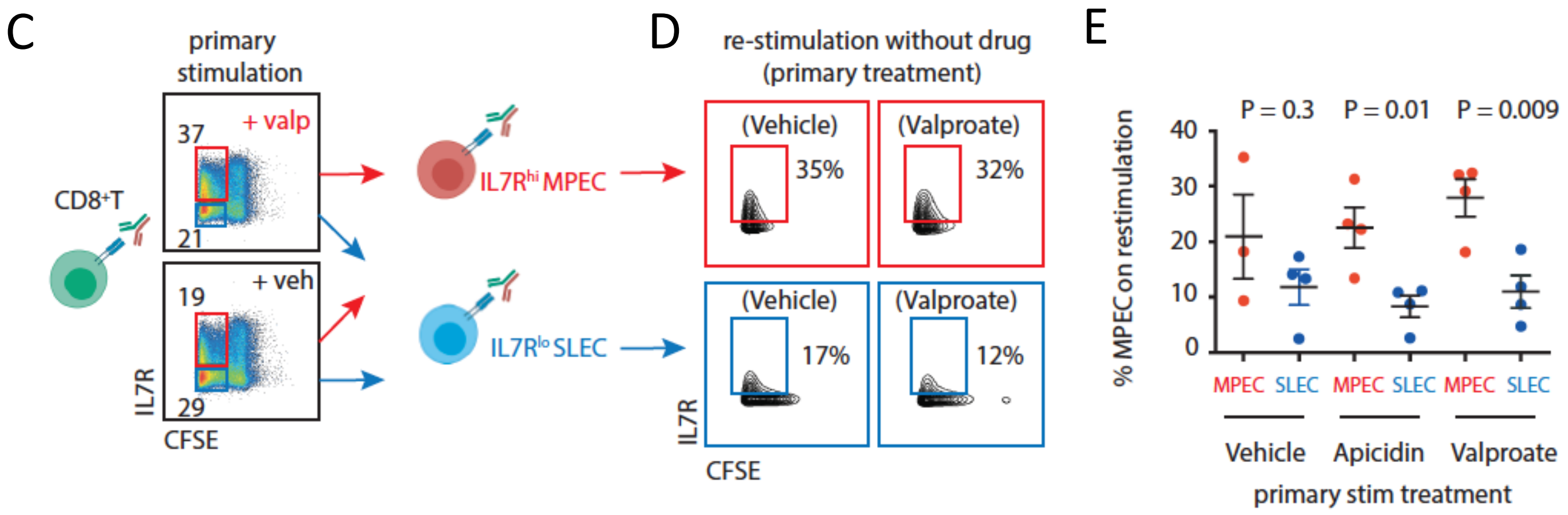

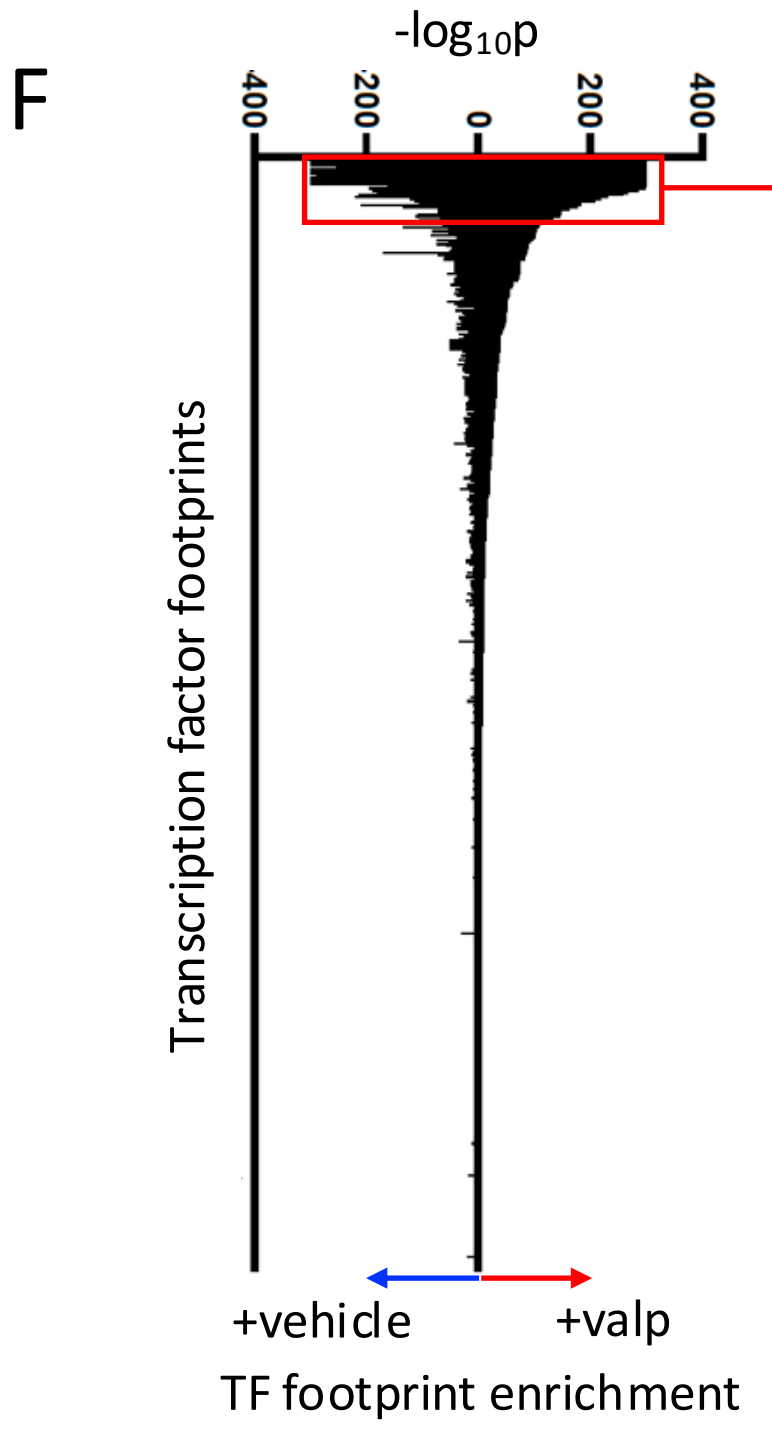

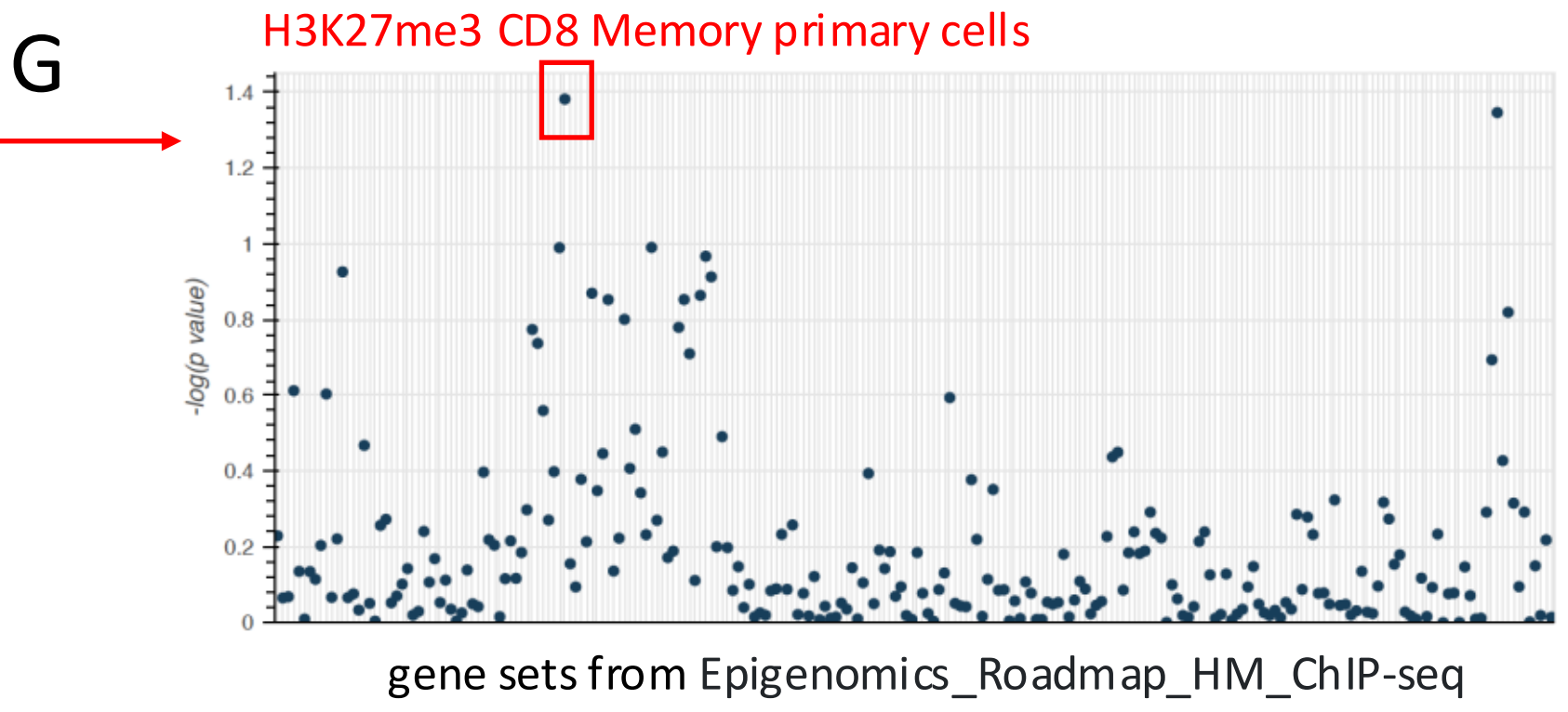

H

TF motif	case_rank	CASEp	control_rank	CONTROLp
OTX2	1	3.00E+02	10	300
CUX2	2	3.00E+02	1	300
CTCF	3	3.00E+02	4	300
SP2	4	3.00E+02	6	300
IRF1	5	3.00E+02	13	252.095294
KLF5	6	3.00E+02	7	300
Arnt	7	3.00E+02	2	300
AhrArnt	8	3.00E+02	3	300
SPIB	9	3.00E+02	12	289.316047
FOSJUN	10	3.00E+02	5	300

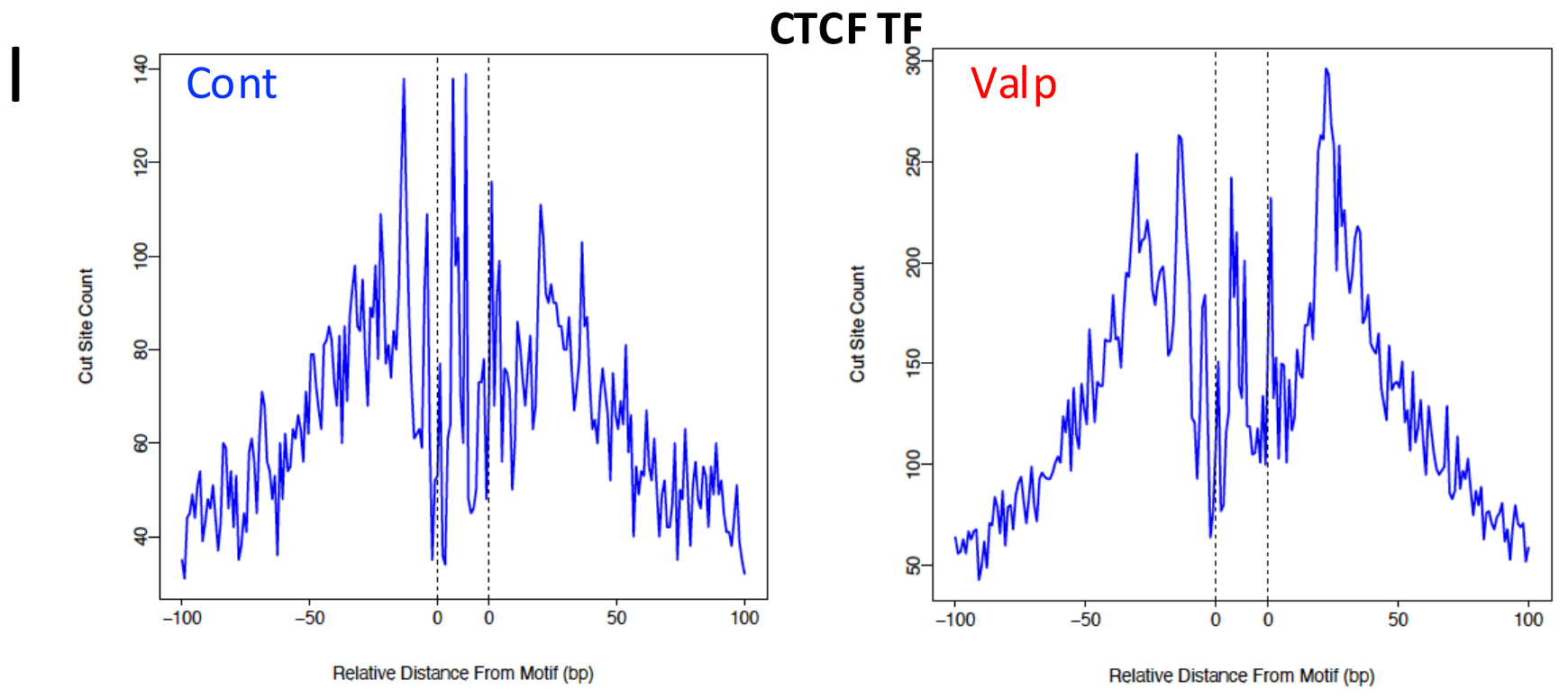

Figure S4. Transcriptomic and functional analysis of KDACi induced memory
(A, B) Annotated line plots showing all expressed genes (x-axis) ranked by weighted contribution to LF2 (B) and LF7 (C) with annotation for selected genes (red) from the TNF/NFkB signalling (B, n=200, M5890) and oxidative phosphorylation (n=200,M5936) pathways. fgsea enrichment (inset) performed on whole pathways with top 50 genes illustrated. (D) Schematic illustration of MPEC ($IL7R^{hi}CFSE^{lo}$) and SLEC ($IL7R^{lo}CFSE^{lo}$) populations on *in vitro* restimulation. (K, L) Representative (E) and summary (F) scatterplots (mean+/-sem) showing MPEC phenotype ($IL7R^{hi}CFSE^{lo}$) on secondary restimulation (without drug treatment) by treatment (apicidin, n=5, valproate, n=5, vehicle n=5) and MPEC phenotype on primary stimulation. (G) Tornado plot showing enrichment ($-\log_{10}p$) of all transcription factor (TF) motifs from the JASPAR repository[109] amongst differential peaks on treatment with valproate (right) or vehicle. The y-values are individual TF motifs (n=519). Manhattan plot (H) showing enrichment of top 10 altered TF footprints (I) amongst terms from the Epigenomics Roadmap HM_ChIP-seq gene set. Each point represents a single term along the x-axis. The y-values represent the $-\log_{10}$(p-value) correspond to the enrichment of the input gene set for each term.

Figure S5

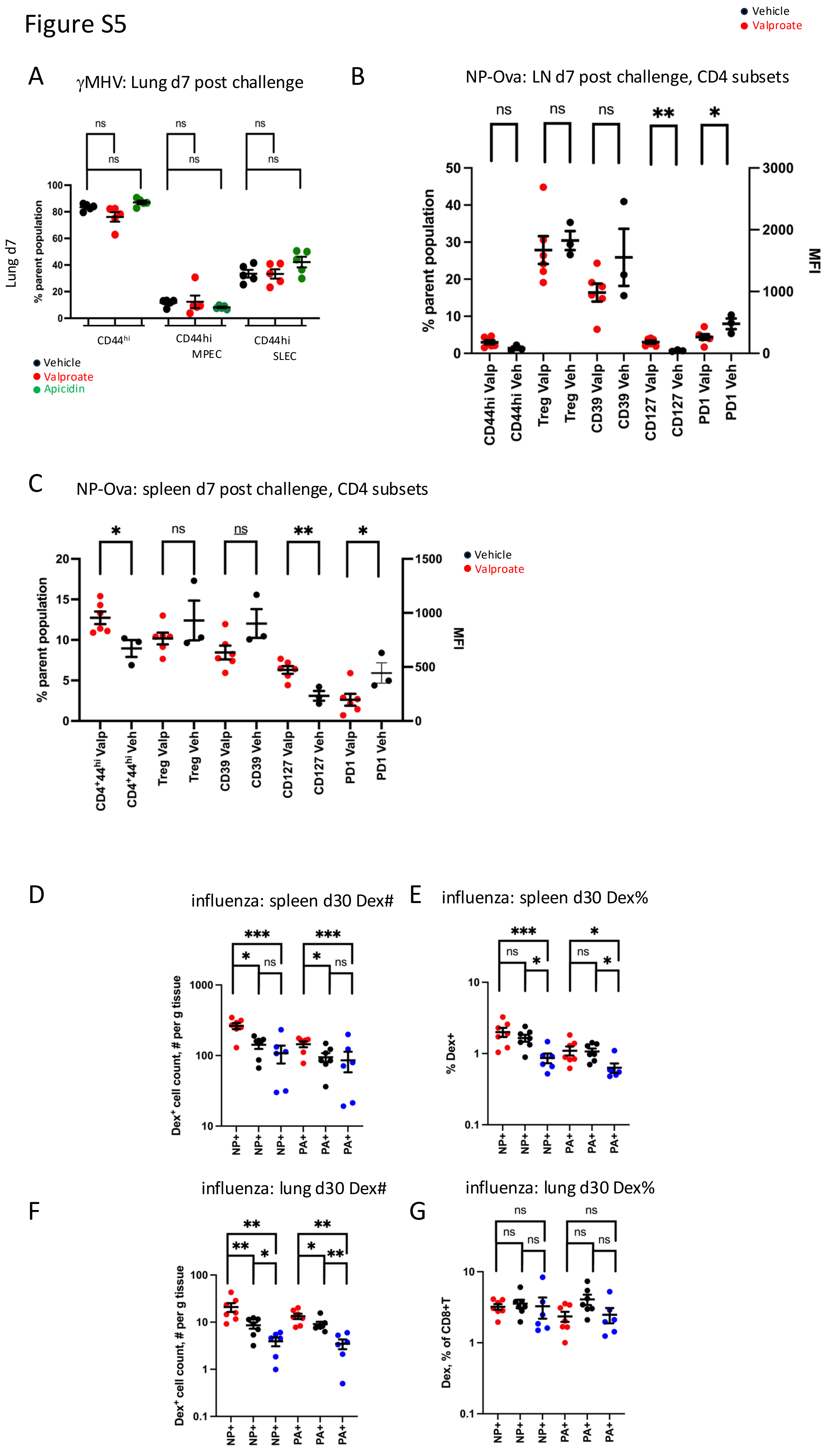

Vehicle
Valproate
A
γMHV: Lung d7 post challenge
ns
ns
ns
ns
ns
ns
Lung d7
% parent population
100
80
60
40
20
0
CD44hi
CD44hi MPEC
CD44hi SLEC
Vehicle
Valproate
Apicidin
B
NP-Ova: LN d7 post challenge, CD4 subsets
ns
ns
ns
**
*
% parent population
50
40
30
20
10
0
3000
2000
1000
0
MFI
CD44hi Valp
CD44hi Veh
Treg Valp
Treg Veh
CD39 Valp
CD39 Veh
CD127 Valp
CD127 Veh
PD1 Valp
PD1 Veh
C
NP-Ova: spleen d7 post challenge, CD4 subsets
*
ns
ns
**
*
% parent population
20
15
10
5
0
1500
1000
500
0
MFI
Vehicle
Valproate
CD4+44hi Valp
CD4+44hi Veh
Treg Valp
Treg Veh
CD39 Valp
CD39 Veh
CD127 Valp
CD127 Veh
PD1 Valp
PD1 Veh
D
influenza: spleen d30 Dex#
***
***
*
ns
*
ns
Dex+ cell count, # per g tissue
1000
100
10
NP+
NP+
NP+
PA+
PA+
PA+
E
influenza: spleen d30 Dex%
***
*
ns
*
ns
*
% Dex+
10
1
0.1
NP+
NP+
NP+
PA+
PA+
PA+
F
influenza: lung d30 Dex#
**
**
**
*
*
**
Dex+ cell count, # per g tissue
100
10
1
0.1
NP+
NP+
NP+
PA+
PA+
PA+
G
influenza: lung d30 Dex%
ns
ns
ns
ns
ns
ns
Dex, % of CD8+T
10
1
0.1
NP+
NP+
NP+
PA+
PA+
PA+

Figure S5. Extended phenotyping of in vivo infection and immunisation models
(A) Scatterplot showing $CD3^{+}8^{+}$ T cell phenotype in lung d7 after intranasal infection with γMHV (as in Fig 4A). (B) Scatterplot showing T cell phenotypic frequencies (left y-axis) and marker median fluorescence (MFI, right y-axis) on $CD3^{+}8^{+}$ T cells in spleen on d7 following challenge with ip NP-Ova (as in Fig 4A). (C) Scatterplot showing $CD4^{+}$ T cell % in spleen on d7 following challenge with ip NP-Ova following valproate (red) or vehicle (black) treatment up to d7. (D-G) Scatterplots showing antigen specific (Dex^{+}) cell counts (D, F) and frequencies (E, G) in spleen (D, E) and lung (F, G) on d30 following primary influenza challenge (as per Fig4E) following treatment with valproate (red), vehicle (black) or combined valproate and R162 (blue) for d1-7. Mann Whitney test, ns = P >0.05, * = P<0.05, ** = P<0.01.

Figure S6

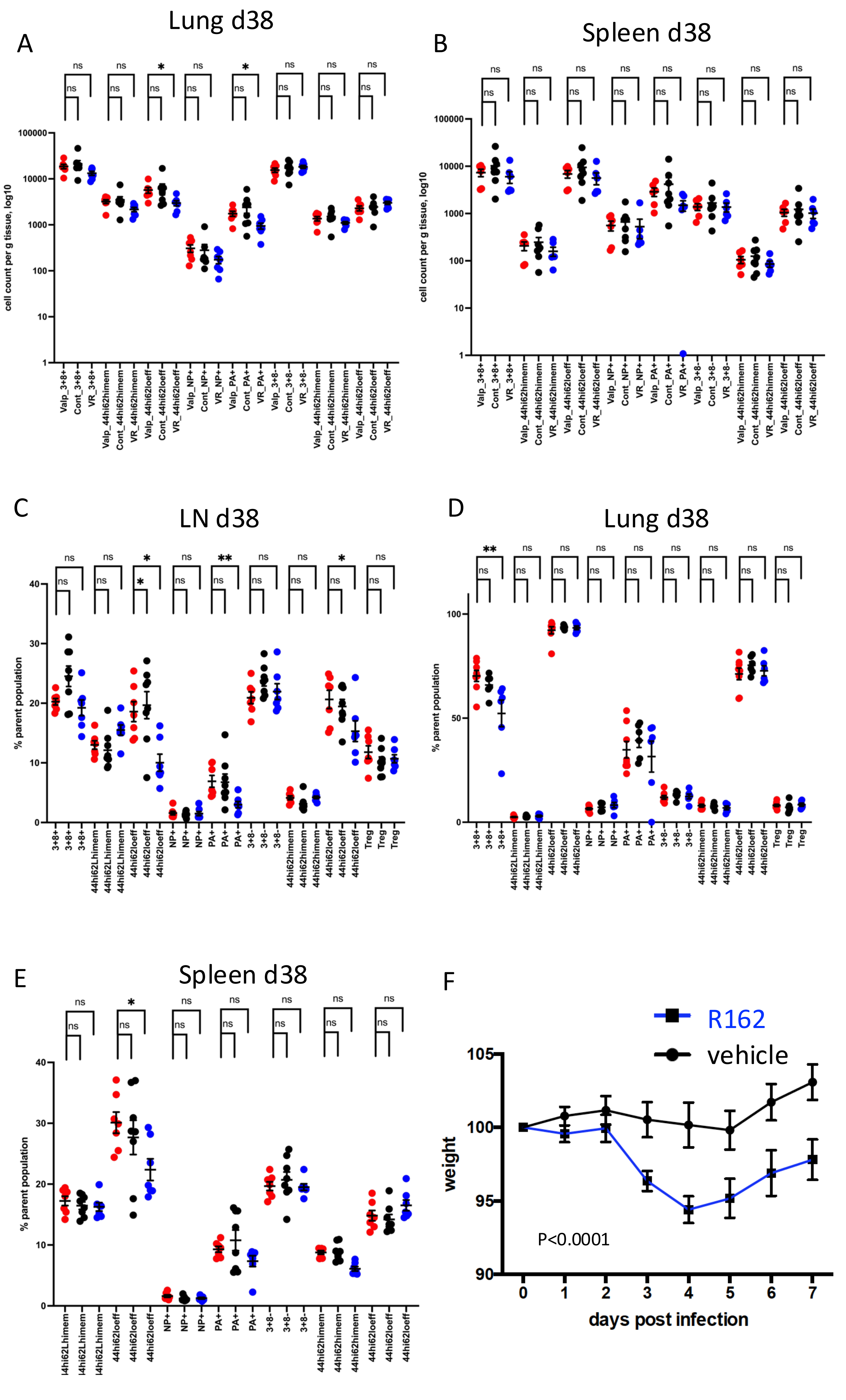

A
Lung d38
cell count per g tissue, log10
Valp_3+8+
Cont_3+8+
VR_3+8+
Valp_44hi62himem
Cont_44hi62himem
VR_44hi62himem
Valp_44hi62loeff
Cont_44hi62loeff
VR_44hi62loeff
Valp_NP+
Cont_NP+
VR_NP+
Valp_PA+
Cont_PA+
VR_PA+
Valp_3+8-
Cont_3+8-
VR_3+8-
Valp_44hi62himem
Cont_44hi62himem
VR_44hi62himem
Valp_44hi62loeff
Cont_44hi62loeff
VR_44hi62loeff
B
Spleen d38
cell count per g tissue, log10
C
LN d38
% parent population
3+8+
44hi62Lhimem
44hi62loeff
NP+
PA+
3+8-
44hi62himem
Treg
D
Lung d38
% parent population
E
Spleen d38
% parent population
F
R162
vehicle
weight
days post infection
P<0.0001

Figure S6. Extended phenotyping of in vivo influenza rechallenge model
Scatterplots (mean +/- sem) illustrating cell counts (A, B, normalized per g tissue) and frequencies (C-E, of parent population) in lung (A, D), spleen (B, E) and draining mediastinal lymph node (C) on day 7 post influenza rechallenge following valproate (V, red), vehicle (black) or combined valproate/R162 (blue) treatment during primary infection (as in Fig4F). ns = P >0.05, * = P<0.05, ** = P<0.01.

Figure S7

A

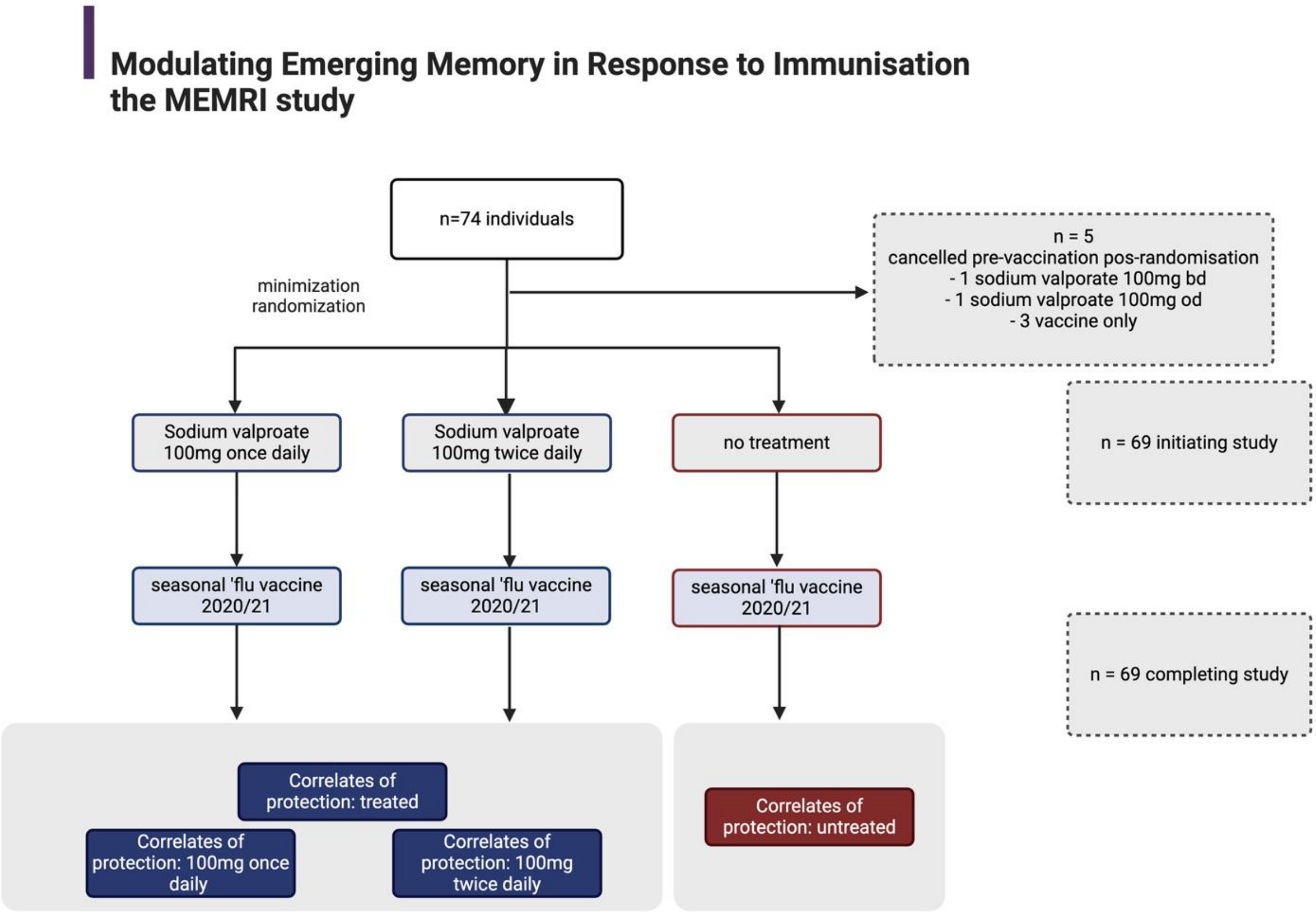

B

Summary	Valp high	Valp low	Control	P
Race	all white	all white	all white	1
smoking	13 never, 9ex	19 never, 4 ex	10 never, 13ex	0.02
temperature	36.6 (0.07)	36.6 (0.11)	36.4 (0.10)	0.23
SBP	130 (3.6)	131.5 (6.1)	128 (3.53)	0.87
DBP	74.45 (1.84)	82 (1.95)	79 (2.29)	0.02
age	49.65 (2.02)	50.3 (2.18)	52.3 (2.30)	0.67
sex	10F, 13M	9F, 14M	10F, 13M	0.99
SAE	0	0	0	1
AE	5Y, 22N	7Y, 16N	7Y, 16N	0.57
BMI	25.03 (1.03)	24.9 (0.85)	24.82 (0.84)	0.98

Figure S7. MEMRI study CONSORT and baseline characteristics
(A) CONSORT diagram illustrating recruitment to the Modulating Emerging Memory Responses to Immunisation (MEMRI) experimental medicine study. The study was powered to detect a difference between valproate treated and untreated individuals. (B) Breakdown of key demographic characteristics of recruited individuals by treatment group. Treatment group allocation was undertaken using a minimization randomization method to ensure balanced allocation by age and sex.

Figure S8

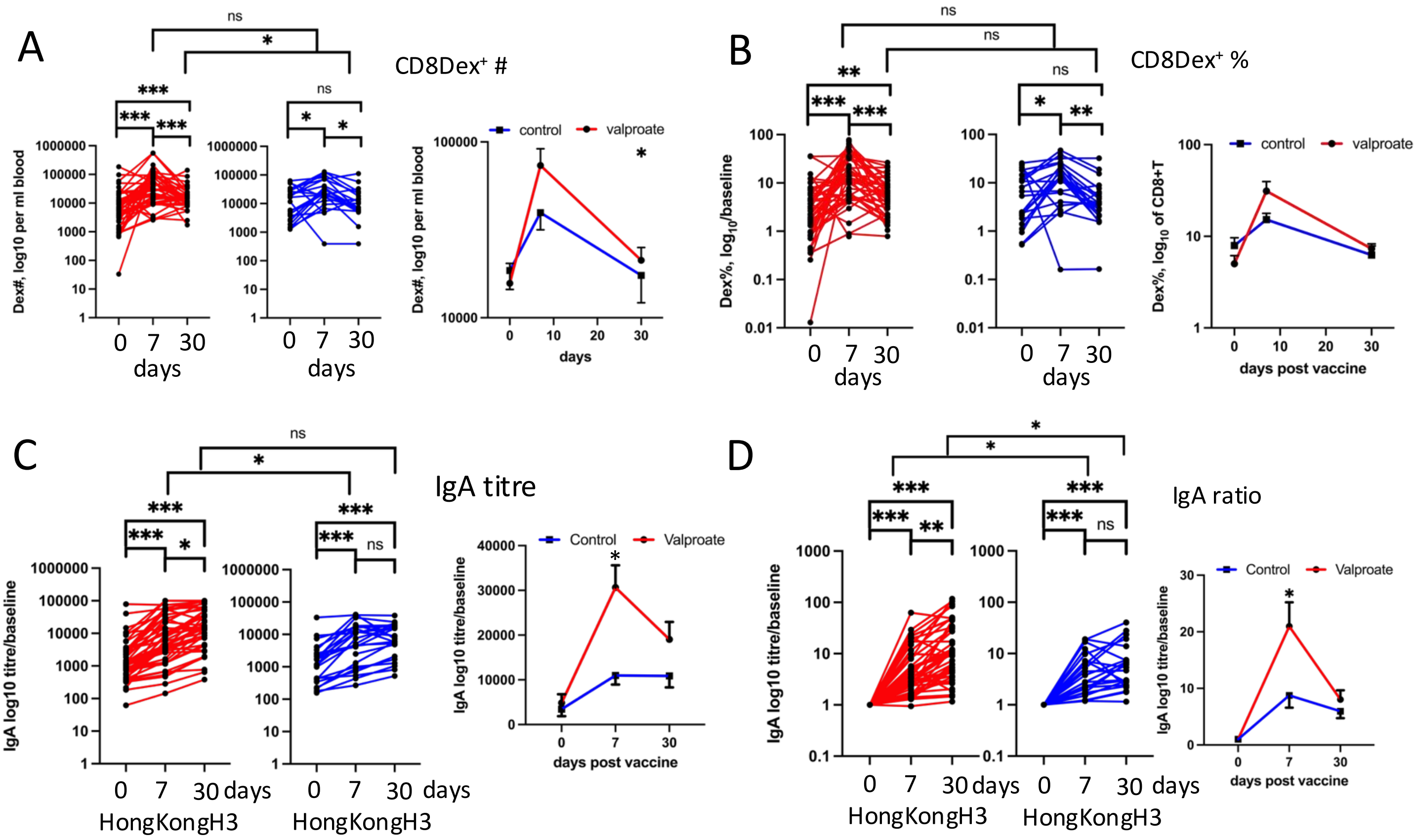

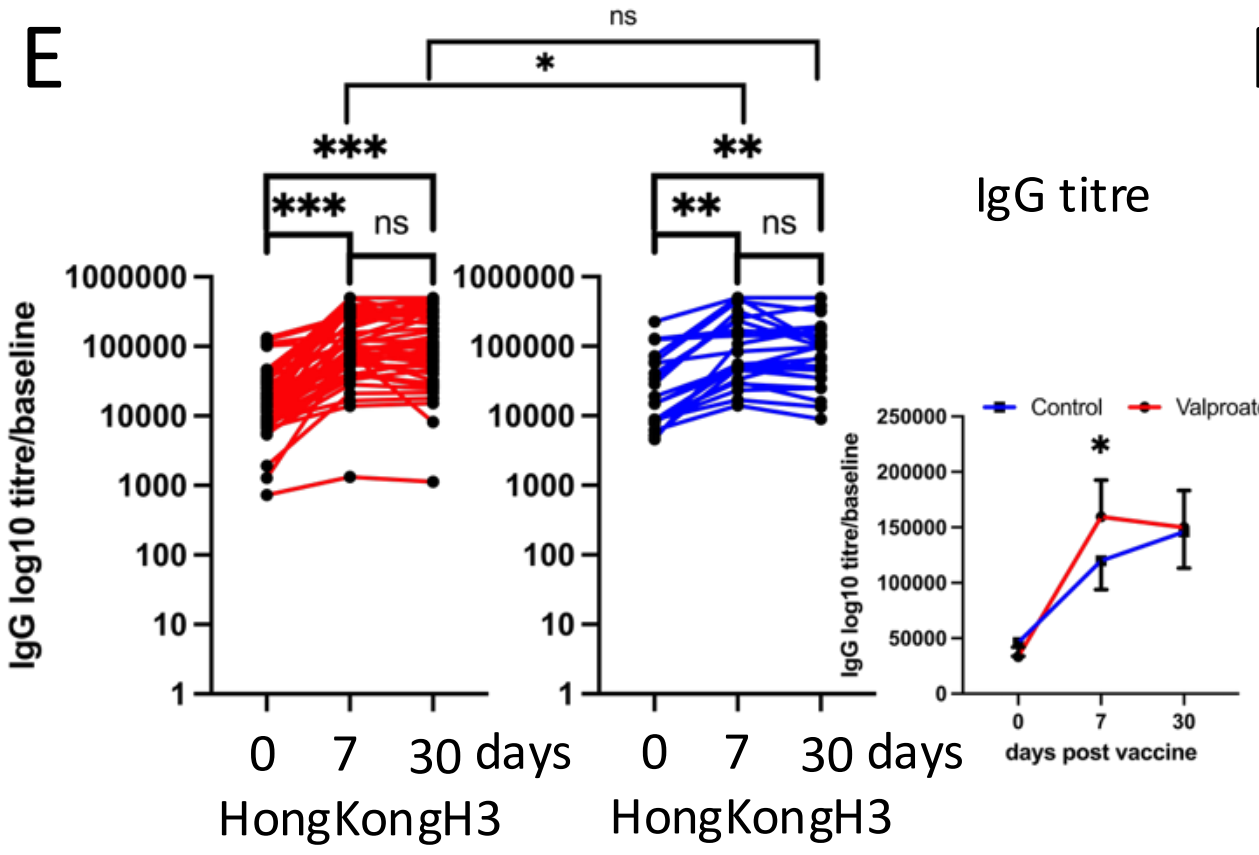

F

CD8⁺ Dextramer ratio			
Predictors	Odds Ratios	CI	p
(Intercept)	10.79	0.36 – 414.94	1.81e-01
Dex8	1.11	1.03 – 1.24	**4.31e-02**
age	0.97	0.93 – 1.02	2.53e-01
BMI	0.99	0.90 – 1.08	7.52e-01
Sex	1.15	0.50 – 2.62	7.46e-01
smoker	0.34	0.14 – 0.77	**1.10e-02**
AE	0.60	0.25 – 1.44	2.53e-01

HongKongH3 Titer ratio			
Predictors	Odds Ratios	CI	p
(Intercept)	8.12	0.08 – 1108.53	3.81e-01
H3HKV3	1.01	0.95 – 1.08	7.65e-01
age	0.99	0.93 – 1.05	7.22e-01
BMI	0.99	0.87 – 1.12	8.36e-01
Sex	0.91	0.30 – 2.68	8.59e-01
smoker	0.38	0.13 – 1.09	7.48e-02
AE	0.61	0.19 – 1.92	3.96e-01

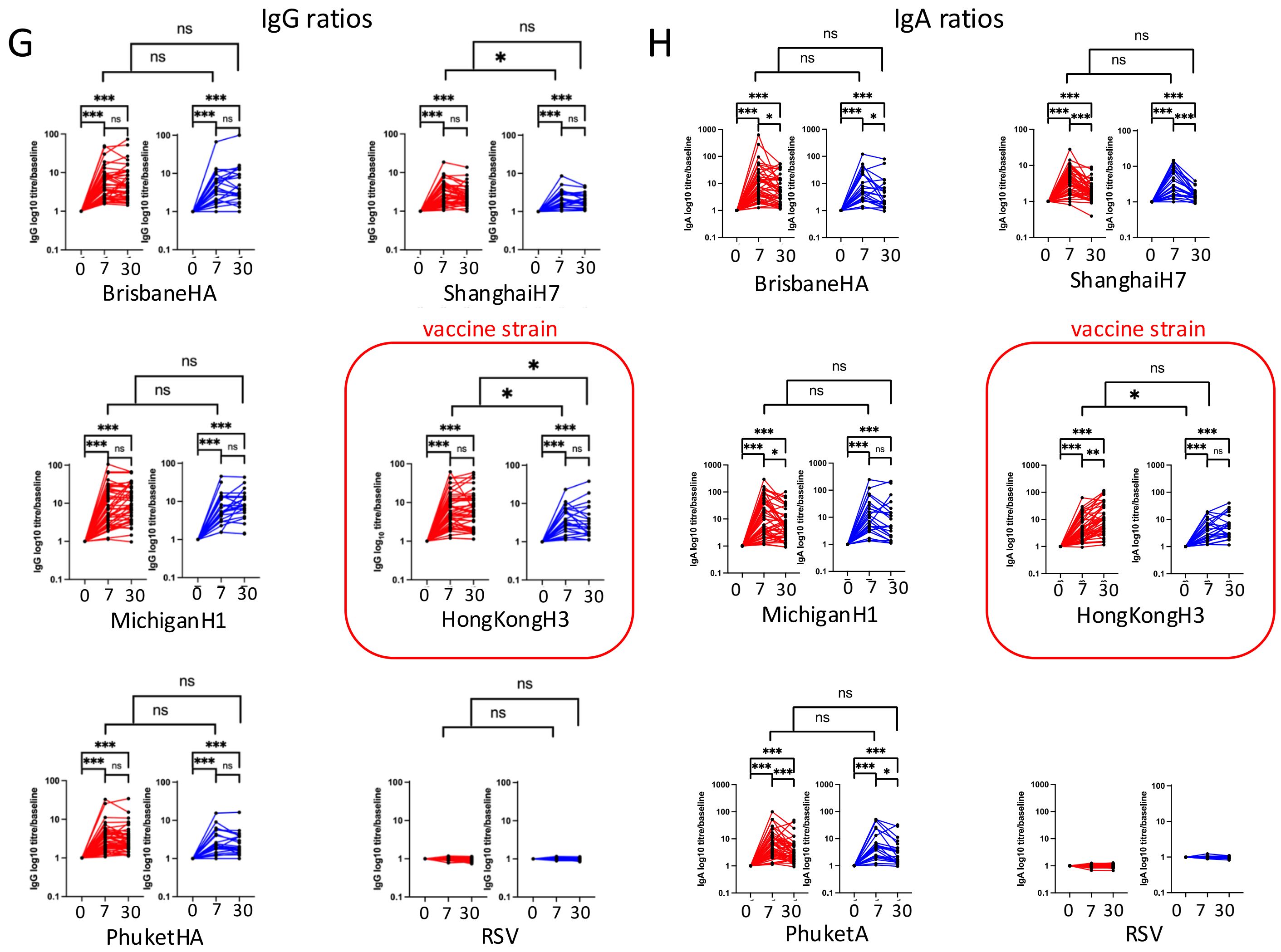

Figure S8. Extended analysis of MEMRI study samples
(A, B) Scatter/line plots showing absolute paired individual (left) and summary (right, mean+/-sem) influenza-specific (Dex^+) $CD8^+$ T cell counts (A) and frequencies (B) by timepoint in volunteers following vaccination with (n=46, red) or without (n=23, blue) valproate treatment at d0, 7 and 30 post vaccination. (C -E) Scatter/line plots showing absolute paired individual (left) and summary (right, mean +/-sem) influenza-specific IgA (C) and IgG titres (E) and baseline-normalized $\log_{10}$ IgA ratios (D) by timepoint in volunteers following vaccination with (n=46, red) or without (n=23, blue) valproate treatment from d1-7 post vaccination. (F) Model summaries from logistic regression analysis of treatment (valp/control) versus cellular (Dex8, left) and humoral (HongKongH3[HKH3], right) vaccine response (ratio vs baseline), incorporating additional covariate effects. AE = adverse events, BMI = body mass index. (G, H) Scatter/line plots showing absolute paired individual (left) and summary (right, mean +/-sem) influenza strain-specific baseline-normalized $\log_{10}$ IgG (G) and IgA ratios (H) by timepoint in volunteers following vaccination with (n=46, red) or without (n=23, blue) valproate treatment from d1-7 post vaccination. Red box indicates the strain included in the seasonal vaccine administered. RSV responses shown as negative control for comparison in each case. Mann-whitney test or Wilcoxon signed rank test (where comparing to baseline ratio =1), ns = $P > 0.05$, * = $P<0.05$, **<0.01, ***<0.001

Figure S9

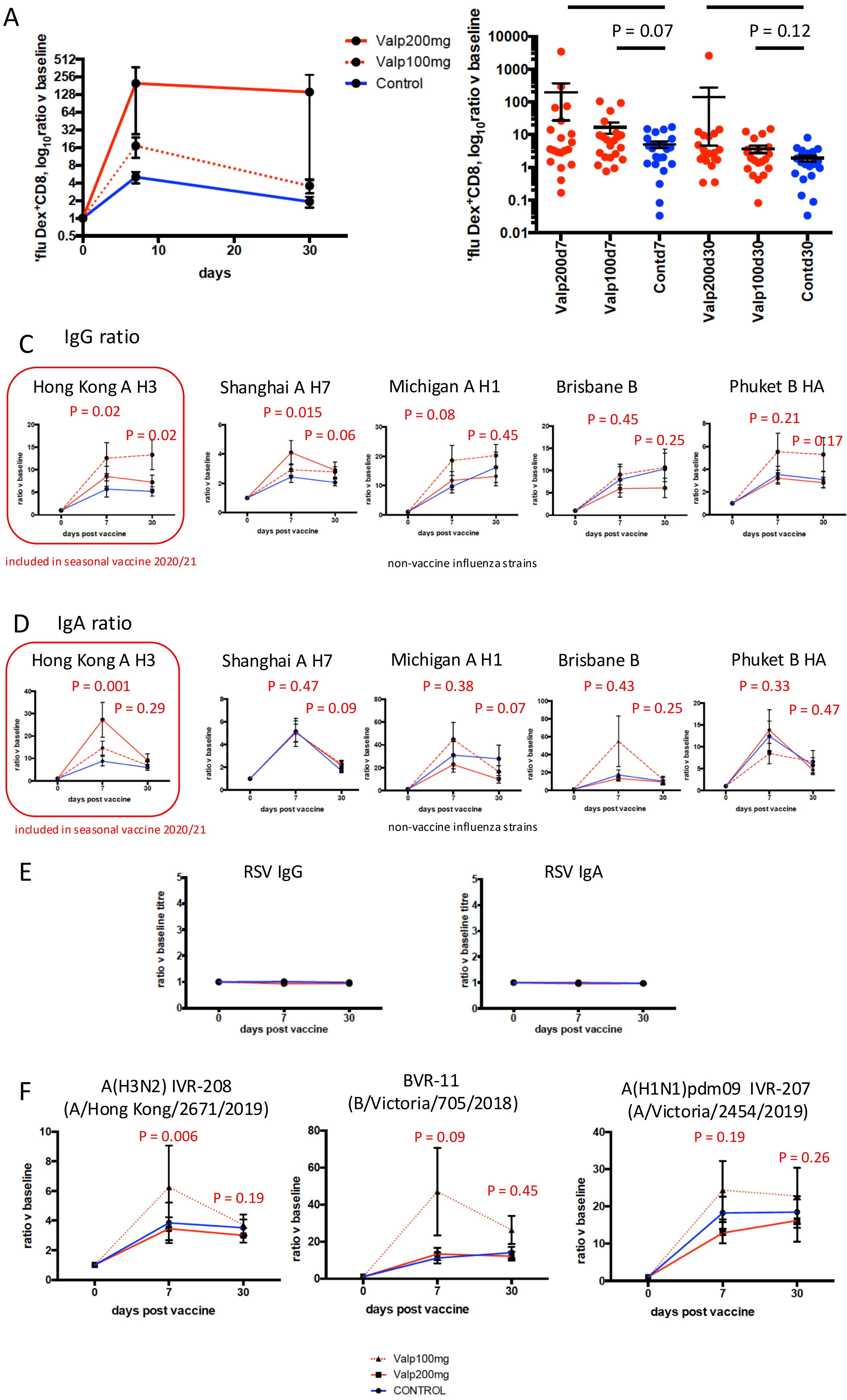

A
'flu Dex+CD8, log10ratio v baseline
512
256
128
64
32
16
8
4
2
1
0.5
0
10
20
30
days
Valp200mg
Valp100mg
Control
B
P = 0.15
P = 0.005
P = 0.07
P = 0.12
'flu Dex+CD8, log10ratio v baseline
10000
1000
100
10
1
0.1
0.01
Valp200d7
Valp100d7
Contd7
Valp200d30
Valp100d30
Contd30
C
IgG ratio
Hong Kong A H3
P = 0.02
P = 0.02
Shanghai A H7
P = 0.015
P = 0.06
Michigan A H1
P = 0.08
P = 0.45
Brisbane B
P = 0.45
P = 0.25
Phuket B HA
P = 0.21
P = 0.17
ratio v baseline
days post vaccine
included in seasonal vaccine 2020/21
non-vaccine influenza strains
D
IgA ratio
Hong Kong A H3
P = 0.001
P = 0.29
Shanghai A H7
P = 0.47
P = 0.09
Michigan A H1
P = 0.38
P = 0.07
Brisbane B
P = 0.43
P = 0.25
Phuket B HA
P = 0.33
P = 0.47
ratio v baseline
days post vaccine
included in seasonal vaccine 2020/21
non-vaccine influenza strains
E
RSV IgG
RSV IgA
ratio v baseline titre
days post vaccine
F
A(H3N2) IVR-208
(A/Hong Kong/2671/2019)
P = 0.006
P = 0.19
BVR-11
(B/Victoria/705/2018)
P = 0.09
P = 0.45
A(H1N1)pdm09 IVR-207
(A/Victoria/2454/2019)
P = 0.19
P = 0.26
ratio v baseline
days post vaccine
Valp100mg
Valp200mg
CONTROL

Figure S9. MEMRI sample analysis by valproate dose group
(A, B) Line plot (A, mean +/- sem) and scatterplot (B, mean+/-sem) showing baseline-normalized influenza-specific $CD8^{+}$ T cell number ($Dextramer^{+}$) up to 30 days post vaccination with the 2020/21 seasonal influenza vaccine and treatment with either 100mg, 200mg valproate or no drug (control). (C-E) Line plots (mean +/- sem) for influenza-specific IgG (C), IgA (D) and RSV (E) by treatment group. (F) Influenza-specific HAI titre by vaccine strain specificity and by treatment group.
P values = one tailed Mann Whitney test.

Figure S10

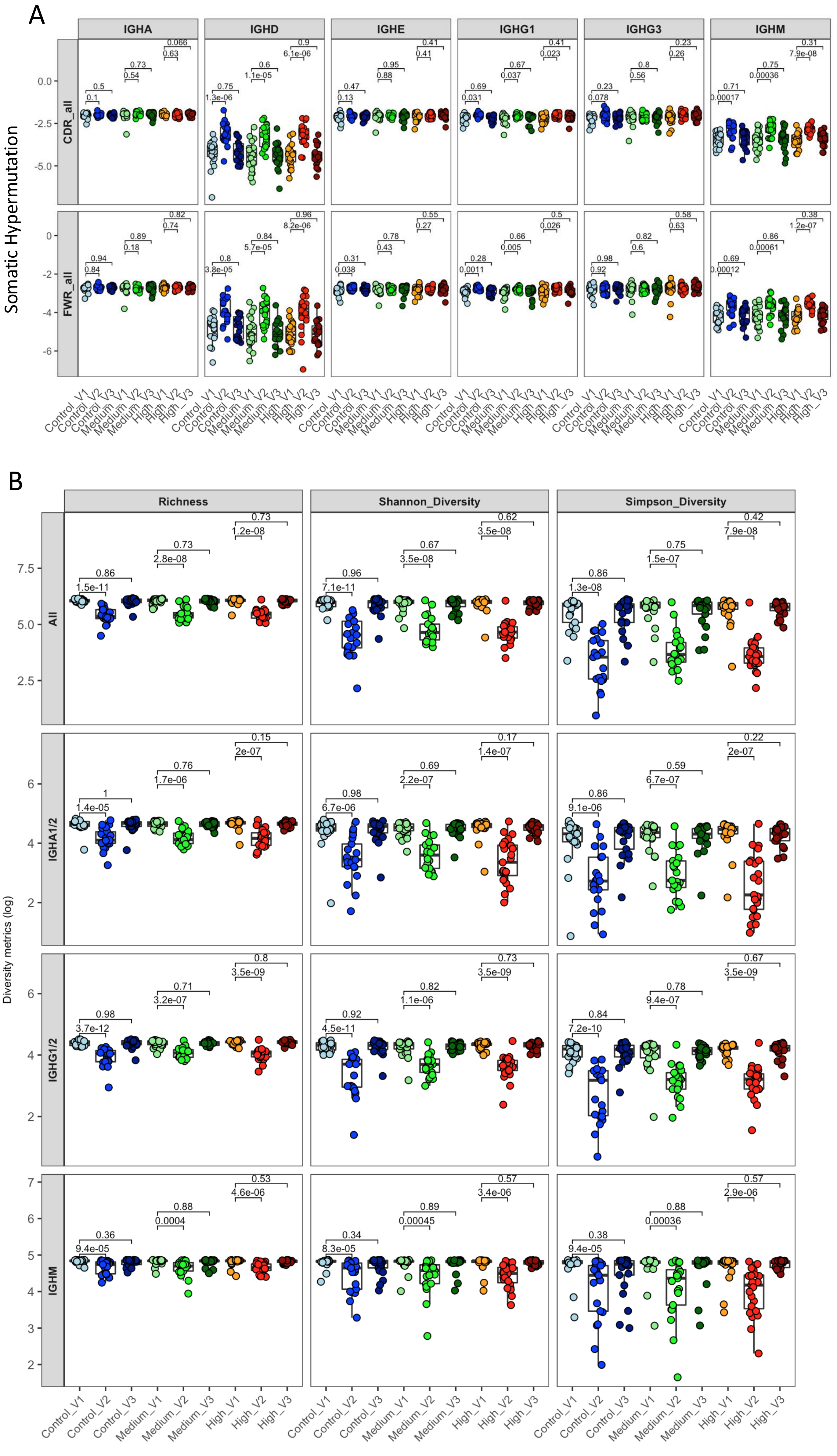
A
IGHA
IGHD
IGHE
IGHG1
IGHG3
IGHM
Somatic Hypermutation
CDR_all
FWR_all
Control_V1
Control_V2
Control_V3
Medium_V1
Medium_V2
Medium_V3
High_V1
High_V2
High_V3
B
Richness
Shannon_Diversity
Simpson_Diversity
All
IGHA1/2
IGHG1/2
IGHM
Diversity metrics (log)

Figure S10. Extended BCR repertoire analysis from the MEMRI study
(A) Total Somatic Hypermutation assessed over complementarity determining regions (CDR) 1+2 and the framework (FWR) region. Results presented by study timepoint (V1-V3), by treatment group (Control, medium [100mg] or high [200mg] valproate dose) and by isotype class of VDJ sequence (columns). (B) Total BCR repertoire diversity metrics (columns) by timepoint (V1-V3), treatment group and VDJ isotype class (rows). 'Richness' refers to the abundance of unique clones in a repertoire, 'Simpson's index' assesses the probability of two randomly sampled reads belonging to the same clone and 'Shannon's index' is a measure of "evenness", whereby the proportion of total reads represented by each clone is assessed.

KEY RESOURCES TABLE

REAGENT or RESOURCE	SOURCE	IDENTIFIER (dilution/conc)
**Antibodies**		
CFSE	Invitrogen	C34554 (2.5uM)
CD45RA, Clone HI100	BioLegend	304110 (1/100)
CD62L, Clone MEL-14	ThermoFisher	25-0621-82 (1/100)
PD1, Clone RMP1-30	Biolegend	329902 (1/100)
IL7R, Clone HIL-7R-M21	BD Biosciences	557938 (1/100)
Hamster anti-human KLRG1, Clone 2F1/KLRG1	BioLegend	138416 (1/100)
CD25, Clone M-A251	BD Biosciences	555749 (1/100)
Live/Dead Aqua Fluorescent dye	Invitrogen	L34957 (1/100)
Acetylated-Lysine (Ac-K^2-100) MultiMab™ Rabbit mAb mix	Cell Signalling Technology	#9814 (1/100)
Acetylated-Lysine (Ac-K^2-100) MultiMab™ Rabbit mAb mix	Cell Signalling Technology	#9814 (1/100)
Goat anti-mouse IgG human ads-HRP	Southern Biotech	1030-05 (1/2000)
anti-mouse interferon gamma	ThermoFisher	AN18 (2ug/ml)
biotinylated anti-mouse IFNγ	ThermoFisher	R4-6A2 (50ul/well)
anti-mouse CD44, Clone IM7	BioLegend	103065 (1/100)
Influenza NP Dextramer, H-2 Db, peptide: ASNENMETM	Immudex	
anti-mouse GL7, Clone GL7	Biolegend	144608 (1/100)
anti-mouse CD8, Clone 53-6.7	Biolegend	100702 (1/100)
anti-mouse BCL6, Clone IG191E/A8	BioLegend	648304 (1/100)
anti-mouse FOXP3, Clone FJK-16s	ThermoFisher	14-5773-82 (1/100)
anti-mouse B220, Clone RA3-6B2	Biolegend	103278 (1/100)
Influenza HLA-A*0201 dextramer, peptide GILGFVFTL	Immudex	
**Bacterial and virus strains**		
γMHV virus	gift from A. Denton, Cambridge Institute for Medical Research	N/A
A/HKx31 (HKx31, H3N2) influenza A virus	gift from S Clare, Wellcome Sanger Institute	N/A
A/PR8/34 (PR8, H1N1) influenza A virus	gift from S Clare, Wellcome Sanger Institute	N/A
SARS-CoV-2 Receptor Binding domain peptide	gift from Y Modis, MRC Laboratory of Molecular Biology	N/A

SARS-CoV-2/human/Liverpool/REMRQ0001/2020	gift from I Goodfellow, University of Cambridge and L Turtle, University of Liverpool, David Matthews and Andrew Davidson (University of Bristol)	N/A
**Biological samples**		
Human Leucocyte cones, primary human peripheral blood cells	NHS Blood and Transplant (Addenbrooke's Hospital, Cambridge, UK)	https://www.blood.co.uk/the-donation-process/about-our-donation-venues/cambridge-donor-centre/
**Chemicals, peptides, and recombinant proteins**		
see Supp Data 2, Supp Data 3 for additional compounds and suppliers	Supp Data 2, Supp Data 3	
NBDG	ThermoFisher	N13195
Interleukin 2	Gibco Life Technologies	10252750
sodium valproate, CAS id 1069-66-5	Sigma Aldrich	S0930000
R162	Focus Biomolecules	10-4603
C968	Sigma	SML1327
BPTES	Sigma	SML0601
DMKG	Sigma	349631
Carboxypeptidase A	Sigma	C9268
Interferon gamma	ThermoFisher	AN18
**Critical commercial assays**		
CD8 microbeads	Miltenyi Biotec	130-045-201
MACS iBEAD polyclonal T cell stimulation	Miltenyi Biotec	130-091-441
Metabolon LC MS/MS metabolomic spectroscopy	https://www.metabolon.com/	
SMARTer stranded Total RNA-Seq kit v2 pico input	Takara Bio	634411
ImmPRESS anti-Rabbit-HRP Polymer detection system	VectorLab	MP-7401
TSA-Plus Opal 620	Akoya	FP1495001KT
Seahorse XF Cell Mito Stress Test Kit	Agilent	103015-100
Seahorse XF Cell Glycolysis Stress Test Kit	Agilent	103020-100
Dual Glo Kit	Promega	N1610
TruCount tubes	BD Biosciences	663028
VPlex Respiratory Virus Panel1	MesoScale Discovery	K15365U-4

Haemagglutination Inhibition assay	WHO Collaborating Centre for Reference and Research on Influenza	VIDRL, Melbourne, Australia
**Deposited data**		
RNA-seq of isolated primary human CD8+ T cells treated with KDACi with polyclonal stimulation for 6 days	EBI ArrayExpress	E-MTAB-13366 https://www.ebi.ac.uk/biostudies/ArrayExpress/studies/E-MTAB-13366?query=E-MTAB-13366
ATAC-seq of isolated primary human CD8+ T cells treated with KDACi with polyclonal stimulation for 6 days	EBI ArrayExpress	E-MTAB-13367 https://www.ebi.ac.uk/biostudies/ArrayExpress/studies/E-MTAB-13367?query=E-MTAB-13367
ATAC-seq of CD44+CD8+ T cells isolated from spleen on 5 post rechallenge in a heterotypic influenza rechallenge model	EBI ArrayExpress	E-MTAB-13368 https://www.ebi.ac.uk/biostudies/ArrayExpress/studies/E-MTAB-13368?query=E-MTAB-13368
Mendeley Data Resource for additional linked data<br>- BCRsequencing fastq files<br>- MEMRI study protocol<br>- Supplementary data files	Mendeley Data Share	https://data.mendeley.com/drafts/pncgztw2mg
**Experimental models: Organisms/strains**		
C57BL/6 wild type mice	Wellcome Sanger Institute	courtesy of S Clare
**Software and algorithms**		
FlowJo	TreeStar	
ape	CRAN	
phylobase	CRAN	
iTOL	https://itol.embl.de/	
EnrichR	https://maayanlab.cloud/Enrichr/	
MetaboAnalyst	https://github.com/xia-lab/MetaboAnalystR	
MOFA+	https://biofam.github.io/MOFA2/	

ImageJ	NIH	
Prism 6.0	graphpad	
Immcantation toolbox	https://immcantation.readthedocs.io/en/stable/	
changeo	https://changeo.readthedocs.io/en/stable/	
IMGT-IV	https://www.imgt.org/IMGTindex/V-QUEST.php	
Alakazam	https://alakazam.readthedocs.io/en/stable/	
esATAC	https://www.bioconductor.org/packages/release/bioc/html/esATAC.html	
CD-HIT	https://www.bioinformatics.org/cd-hit/	
