# Supplementary material for: Multiomic analysis identifies glutaminolysis-dependent metabolic enhancement of immune memory utilised for vaccine development
Source: Nat Commun. 2026 Jun 26;17:8018. doi: 10.1038/s41467-026-74866-4 (PMC13454449; doi:10.1038/s41467-026-74866-4)
Supplement: Supplementary file 3 [file 41467_2026_74866_MOESM2_ESM.pdf]

Description of Additional Supplementary Files

Supplementary Data 1. Transcriptional signatures used for in silico screening

Supplementary Data 2. Treatments selected for in vitro screening

Supplementary Data 3. KDACi dose range selection and supporting information

Supplementary Data 4. Differential Metabolomic analysis of stimulated primary CD8+ T cells with valproate or apicidin treatment

Supplementary Data 5. Metabolomic data

Supplementary Data 6. Transcriptional signatures defining T cell differentiation states
