# Supplementary material for: Multiomic analysis identifies glutaminolysis-dependent metabolic enhancement of immune memory utilised for vaccine development
Source: Nat Commun. 2026 Jun 26;17:8018. doi: 10.1038/s41467-026-74866-4 (PMC13454449; doi:10.1038/s41467-026-74866-4)
Supplement: Supplementary file 4 [file 41467_2026_74866_MOESM9_ESM.pdf]

nature portfolio

Corresponding author(s): Eoin McKinney

Last updated by author(s): 23/02/2026

Reporting Summary

Nature Portfolio wishes to improve the reproducibility of the work that we publish. This form provides structure for consistency and transparency in reporting. For further information on Nature Portfolio policies, see our Editorial Policies and the Editorial Policy Checklist.

Statistics

For all statistical analyses, confirm that the following items are present in the figure legend, table legend, main text, or Methods section.

n/a	Confirmed	
☐	☒	The exact sample size (*n*) for each experimental group/condition, given as a discrete number and unit of measurement
☐	☒	A statement on whether measurements were taken from distinct samples or whether the same sample was measured repeatedly
☐	☒	The statistical test(s) used AND whether they are one- or two-sided<br>*Only common tests should be described solely by name; describe more complex techniques in the Methods section.*
☐	☒	A description of all covariates tested
☐	☒	A description of any assumptions or corrections, such as tests of normality and adjustment for multiple comparisons
☐	☒	A full description of the statistical parameters including central tendency (e.g. means) or other basic estimates (e.g. regression coefficient) AND variation (e.g. standard deviation) or associated estimates of uncertainty (e.g. confidence intervals)
☐	☒	For null hypothesis testing, the test statistic (e.g. *F*, *t*, *r*) with confidence intervals, effect sizes, degrees of freedom and *P* value noted<br>*Give P values as exact values whenever suitable.*
☒	☐	For Bayesian analysis, information on the choice of priors and Markov chain Monte Carlo settings
☒	☐	For hierarchical and complex designs, identification of the appropriate level for tests and full reporting of outcomes
☐	☒	Estimates of effect sizes (e.g. Cohen's *d*, Pearson's *r*), indicating how they were calculated

Our web collection on statistics for biologists contains articles on many of the points above.

Software and code

Policy information about availability of computer code

Data collection	No custom software or code was used in collection of data for this analysis. Software packages used are detailed in the Key Resources Table provided.
Data analysis	No custom software or code was used in analsyis of data. Software packages and algorithms used are detailed in the Key Resources Table provided<br><br>Software and algorithms<br>FlowJo TreeStar<br>ape CRAN<br>phylobase CRAN<br>iTOL https://itol.embl.de/<br><br>EnrichR https://maayanlab.cloud/Enrichr/<br>MetaboAnalyst https://github.com/xia-lab/MetaboAnalystR<br>MOFA+ https://biofam.github.io/MOFA2/<br>ImageJ NIH<br>Prism 6.0 graphpad<br>Immcantation toolbox https://immcantation.readthedocs.io/en/stable/<br>changeo https://changeo.readthedocs.io/en/stable/<br>IMGT-IV https://www.imgt.org/IMGTindex/V-QUEST.php<br>Alakazam https://alakazam.readthedocs.io/en/stable/

esATAC https://www.bioconductor.org/packages/release/bioc/html/esATAC.html
CD-HIT https://www.bioinformatics.org/cd-hit/

For manuscripts utilizing custom algorithms or software that are central to the research but not yet described in published literature, software must be made available to editors and reviewers. We strongly encourage code deposition in a community repository (e.g. GitHub). See the Nature Portfolio guidelines for submitting code & software for further information.

Data

Policy information about availability of data

All manuscripts must include a data availability statement. This statement should provide the following information, where applicable:

- Accession codes, unique identifiers, or web links for publicly available datasets
- A description of any restrictions on data availability
- For clinical datasets or third party data, please ensure that the statement adheres to our policy

RNA-seq data have been deposited in EBI ArrayExpress and are publicly available as of the date of publication under the accession numbers: E-MTAB-13366 (RNA-seq of isolated primary human CD8+ T cells treated with KDACi with polyclonal stimulation for 6 days), E-MTAB-13367 (ATAC-seq of isolated primary human CD8+ T cells treated with KDACi with polyclonal stimulation for 6 days), E-MTAB-13368 (ATAC-seq of CD44+CD8+ T cells isolated from spleen on 5 post rechallenge in a heterotypic influenza rechallenge model). Additional raw datasets and full protocols have been deposited at Mendeley and are publicly available as of the date of publication (https://data.mendeley.com/drafts/pncgztw2mg). Accession codes and DOI are also listed in the key resources table.

Research involving human participants, their data, or biological material

Policy information about studies with human participants or human data. See also policy information about sex, gender (identity/presentation), and sexual orientation and race, ethnicity and racism.

Reporting on sex and gender	Sex was accounted for during participant recruitment using a minimization randomization method to ensure balanced allocation by age and sex as described in methods.
Reporting on race, ethnicity, or other socially relevant groupings	Recruitment was not restricted by ethnicity although all individuals recruited were self-reported caucasian. This is primarily due to their recruitment from the NIHR BioResource that is based in Cambridge, UK.
Population characteristics	Population characteristics of the cohort recruited are outlined in Figure S7.
Recruitment	Full details of recruitment are provided in Methods with additional information in the full experimental medicine study protocol, provided in Supplementary Information.
Ethics oversight	Animal experiments where performed under project licence P653704A5 with institutional approval by a local Animal Welfare and Ethical Review Board (AWERB) and in accordance with the United Kingdom Animals (Scientific Procedures) Act of 1986. The Modulating Emerging Memory Responses to Immunisation (MEMRI) study was a non-CTIMP interventional experimental medicine study as defined by EU Directive 2001/20/EC and approved by the HRA and Health and Care Research Wales (IRAS id 287814). The study has been verified by a formal MHRA scoping review as an experimental medicine study exploring correlates of protection, therefore not requiring registration as a clinical trial.

Note that full information on the approval of the study protocol must also be provided in the manuscript.

Field-specific reporting

Please select the one below that is the best fit for your research. If you are not sure, read the appropriate sections before making your selection.

☒ Life sciences ☐ Behavioural & social sciences ☐ Ecological, evolutionary & environmental sciences

For a reference copy of the document with all sections, see nature.com/documents/nr-reporting-summary-flat.pdf

Life sciences study design

All studies must disclose on these points even when the disclosure is negative.

Sample size	We have undertaken estimates of sample size using the available prior information. 2-sample non-inferiority tests have indicated:<br>- For cellular memory responses: A group size of n=25 results in 80% power to detect a superiority margin of 30 with group A mean 110 and group B mean 55 with sd 50 and a sampling ratio of 1 at a type 1 error rate of 0.05 (http://powerandsamplesize.com/Calculators/Compare-2-Means/2-Sample-Non-Inferiority-or-Superiority)<br>- For humoral memory responses: A group size of n=36 results in 80% power to detect a superiority margin of 2 with group A mean 18 and group B mean 32 with sd 32 and a sampling ratio of 1 at a type 1 error rate of 0.05 ((http://powerandsamplesize.com/Calculators/Compare-

	2-Means/2-Sample-Non-Inferiority-or-Superiority). Full details of power calculations and assumptions made are available in the MEMRI study protocol, Section 7.1.
Data exclusions	No data exclusions were made prior to analysis.
Replication	The data presented in the figures represent a minimum of three independent experiments. The number, statistical test and descriptive metrics used are defined in each corresponding figure and its legend.
Randomization	Sequential participants (n=74) were randomized at recruitment into one of three treatment groups (sodium valproate 100mg od, 100mg bd or control without treatment) using a biased coin minimization and marginal balance method (probability 0.7, MinimPy2) specifying age (18-30, 30-45, 46-65 years old) and sex (male or female) as stratification factors.
Blinding	For in vitro and animal studies, investigators were blinded to the treatment group of the samples until after results were obtained and recorded. For the MEMRI study, Investigators were blinded to the study group allocation during analysis.

Reporting for specific materials, systems and methods

We require information from authors about some types of materials, experimental systems and methods used in many studies. Here, indicate whether each material, system or method listed is relevant to your study. If you are not sure if a list item applies to your research, read the appropriate section before selecting a response.

Materials & experimental systems

n/a	Involved in the study	
☐	☒	Antibodies
☐	☒	Eukaryotic cell lines
☐	☐	Palaeontology and archaeology
☐	☒	Animals and other organisms
☐	☒	Clinical data
☒	☐	Dual use research of concern
☒	☐	Plants

Methods

n/a	Involved in the study	
☒	☐	ChIP-seq
☐	☒	Flow cytometry
☒	☐	MRI-based neuroimaging

Antibodies

Antibodies used

All antibodies used, including clone IDs, suppliers and target antigens are made availlabe in the Key Resources Table.
Antibodies (validation study in brackets)
CFSE Invitrogen C34554
CD45RA, Clone HI100 BioLegend 304110 (Mol Ther
. 2014 Jul;22(7):1388-1395. doi: 10.1038/mt.2014.50.)
CD62L, Clone MEL-14 ThermoFisher 25-0621-82 (Immunity, 58(3), 555. (PMID:39879983))
PD1, Clone RMP1-30 Biolegend 329902 (Immunity, 2021 Mar 9;54(3):526-541.e7. doi: 10.1016/j.immuni.2021.01.003.)
IL7R, Clone HIL-7R-M21 BD Biosciences 557938 (Immunity, 2026 Jan 13;59(1):129-144.e13. doi: 10.1016/j.immuni.2025.10.020
Hamster anti-human KLRG1, Clone 2F1/KLRG1 BioLegend 138416 (Grebinoski S, et al. 2022. Nat Immunol. 23:868)
CD25, Clone M-A251 BD Biosciences 555749 (Nat Med, 2025 Nov;31(11):3879-3888. doi: 10.1038/s41591-025-03993-0.)
Live/Dead Aqua Fluorescent dye Invitrogen L34957
Acetylated-Lysine (Ac-K2-100) MultiMab™ Rabbit mAb mix Cell Signalling Technology #9814 (validated using SimpleChIP® Enzymatic Chromatin IP Kits)
Goat anti-mouse IgG human ads-HRP Southern Biotech 1030-05 (Nat Commun, 2025 Dec 22;16(1):11561. doi: 10.1038/s41467-025-66252-3.)
anti-mouse interferon gamma ThermoFisher AN18 (Sci Immunol, 2023 Sep 29;8(87):eadd1599. doi: 10.1126/sciimmunol.add1599)
biotinylated anti-mouse IFN� ThermoFisher R4-6A2 (JCI Insight, 2018 Feb 22;3(4):e95824. doi: 10.1172/jci.insight.95824.)
anti-mouse CD44, Clone IM7 BioLegend 103065 (Campisi L, et al. 2022. Nature. 606:945.)
Influenza NP Dextramer, H-2 Db, peptide: ASNENMETM Immudex
anti-mouse GL7, Clone GL7 Biolegend 144608 (Zhang YN, et al. 2023. Nat Commun. 14:1985. PubMed)
anti-mouse CD8, Clone 53-6.7 Biolegend 100702 (Cell, 2024 Jun 6;187(12):3090-3107.e21. doi: 10.1016/j.cell.2024.04.018.)
anti-mouse BCL6, Clone IG191E/A8 BioLegend 648304 (Venturutti L, et al. 2020. Cell. 182(2):297-316.e27)
anti-mouse FOXP3, Clone FJK-16s ThermoFisher 14-5773-82 (J Clin Invest, 2018 Jul 2;128(7):3088-3101. doi: 10.1172/JCI97490.)
anti-mouse B220, Clone RA3-6B2 Biolegend 103278 (Fazilleau N, et al. 2007. Nature Immunol. 8:753.)
Influenza HLA-A*0201 dextramer, peptide GILGFVFTL Immudex

Validation

Antibodies were used according to the manufacturer's instructions with Fluorescence minus one controls where indicated.

Eukaryotic cell lines

Policy information about cell lines and Sex and Gender in Research

Cell line source(s)

HEK293T reporter cell line, gift from Dr Nicholas Matheson

Authentication	authenticated by STR profiling
Mycoplasma contamination	All cell lines tested negative for mycoplasma contamination
Commonly misidentified lines (See ICLAC register)	None

Palaeontology and Archaeology

Specimen provenance	*Provide provenance information for specimens and describe permits that were obtained for the work (including the name of the issuing authority, the date of issue, and any identifying information). Permits should encompass collection and, where applicable, export.*
Specimen deposition	*Indicate where the specimens have been deposited to permit free access by other researchers.*
Dating methods	*If new dates are provided, describe how they were obtained (e.g. collection, storage, sample pretreatment and measurement), where they were obtained (i.e. lab name), the calibration program and the protocol for quality assurance OR state that no new dates are provided.*

☐ Tick this box to confirm that the raw and calibrated dates are available in the paper or in Supplementary Information.

Ethics oversight	*Identify the organization(s) that approved or provided guidance on the study protocol, OR state that no ethical approval or guidance was required and explain why not.*

Note that full information on the approval of the study protocol must also be provided in the manuscript.

Animals and other research organisms

Policy information about studies involving animals; ARRIVE guidelines recommended for reporting animal research, and Sex and Gender in Research

Laboratory animals	Mouse experiments were carried out in specific pathogen-free conditions at The Wellcome Trust Sanger Institute. Experiments were performed under project licence P653704A5 with institutional approval by a local Animal Welfare and Ethical Review Board (AWERB) and in accordance with the United Kingdom Animals (Scientific Procedures) Act of 1986. Basic husbandry and animal welfare was provided daily by trained and competent animal technicians and mice were given ad libitum access to food (Safe Diets, Safe 105) and water in individually ventilated cages with relative humidity (RH) of 55 ± 10%, temperature 21+/-2C and a standard 12h light/dark cycle. All animals were bred at the Wellcome Sanger Institute and were wild type mus musculus C57BL/6N(WTSI) and were between 6-8 weeks of age.
Wild animals	No wild animals were used in this study.
Reporting on sex	Only female mice were used in this study.
Field-collected samples	N/A
Ethics oversight	Experiments where performed under project licence P653704A5 with institutional approval by a local Animal Welfare and Ethical Review Board (AWERB) and in accordance with the United Kingdom Animals (Scientific Procedures) Act of 1986.

Note that full information on the approval of the study protocol must also be provided in the manuscript.

Clinical data

Policy information about clinical studies
All manuscripts should comply with the ICMJE guidelines for publication of clinical research and a completed CONSORT checklist must be included with all submissions.

Clinical trial registration	The Modulating Emerging Memory Responses to Immunisation (MEMRI) study was a non-CTIMP interventional experimental medicine study as defined by EU Directive 2001/20/EC and approved by the HRA and Health and Care Research Wales (IRAS id 287814).
Study protocol	The full MEMRI study protocol is available through Mendeley Data Share with link details provided in the Key Resources Table.
Data collection	Participants were identified from the NIHR Cambridge BioResource (study# CBR213) with informed consent taken from all participants. Prespecified inclusion and exclusion criteria were: [for inclusion] age >18 years old, able to give informed consent and hetero/homozygous for HLA-A*02:01; [for exclusion] known/suspected primary or acquired immune deficiencies due to a congenital condition, treatment or disease process, history of thymectomy, any use of immune suppressing medications, including glucocorticoids and topical formulations, current or within the previous 60 days, prior vaccination with or contraindication to receiving the 2020/21 seasonal influenza vaccine, any intercurrent acute febrile illness, active or in the previous 90 days and pregnancy, lactation or intention to conceive during the study period in women of childbearing potential. Individuals >65 years of age were precluded by recommendation of the Cambridge BioResource Advisory Board due to the COVID pandemic and requirements

	for hospital attendance for venepuncture. Vaccines were administered in and samples were collected in the Cambridge Clinical Research Centre (CCRC), Addenbrooke's Hospital, Hills Road, Cambridge, CB2 0XY between August 2020 and March 2021.
Outcomes	The population we aim to study are healthy adults in two broad age groups (18-65 years and >65 years) who are positive for specific HLA alleles that will facilitate identification of antigen specific CD4 (HLA-DRB*07:01, HLA-DRB*11:01, HLA-DRB*04:01) and CD8 (A*02:01) T cells. Individuals will be identified and invited to participate through the NIHR Cambridge BioResource, to facilitate identification by HLA haplotype.<br>We aim to recruit up to 180 volunteers, to achieve full datasets from 50 volunteers per treatment group (1.vaccine,+drug.dose#1 (100mg/day), 2.vaccine+drug.dose#2 (200mg/day) of the study (allowing for 20% dropout). Group 3 will receive seasonal influenza immunization alone with groups 1 and 2 receiving oral low dose sodium valproate from day 0 to day 7 inclusive. Each group will be further stratified by age into those 18-65 years old and those >65 years old (Figure 1). Peripheral blood samples will be collected before and after vaccination (day0 and day7) and will be processed to retain serum and peripheral blood mononuclear cells (PBMC) for storage as viable aliquots of 107 cells. Samples will be processed in batches to minimize batch artefact structure once all have been collected. Serum samples will be used to quantitate influenza specific humoral responses (ELISA, HAI titre) at each timepoint while frozen, viable cell aliquots will be used to identify and sort influenza specific CD8 and CD4 T cells for quantitation and sequencing.<br>The immune traits being measured as outcomes in this study design have been developed taking into account previous evidence of immunological correlates of protection following influenza vaccination:<br><br>• The cellular response following influenza vaccine shows a significant correlation with protection from severe infection.<br>• Increased titre of influenza specific antibody is a correlate of protection from influenza infection following seasonal vaccination<br>• Influenza responses to seasonal vaccination are demonstrably lower in aged individuals compared to younger vaccines.<br>• There is a dose-dependent effect of sodium valproate on memory differentiation during polyclonal stimulation of T lymphocytes in vitro culture.<br>• There is a differential effect of sodium valproate on memory and naïve populations during polyclonal stimulation of T lymphocytes in vitro culture.<br><br>1) Primary: The magnitude, proportion and phenotype of the influenza-specific memory response at 7 and 30 days post immunisation.<br>2) Secondary:<br>The impact of the following key parameters on the primary endpoint:<br>i. Drug dose<br>ii. Vaccinee age<br>iii. Prior level of influenza-specific humoral and cellular immunity.<br>Full details on primary and secondary outcomes and the rationale for their selection are available in the Study protocol, with link provided through the Key Resources Table.

Plants

Seed stocks	*Report on the source of all seed stocks or other plant material used. If applicable, state the seed stock centre and catalogue number. If plant specimens were collected from the field, describe the collection location, date and sampling procedures.*
Novel plant genotypes	*Describe the methods by which all novel plant genotypes were produced. This includes those generated by transgenic approaches, gene editing, chemical/radiation-based mutagenesis and hybridization. For transgenic lines, describe the transformation method, the number of independent lines analyzed and the generation upon which experiments were performed. For gene-edited lines, describe the editor used, the endogenous sequence targeted for editing, the targeting guide RNA sequence (if applicable) and how the editor was applied.*
Authentication	*Describe any authentication procedures for each seed stock used or novel genotype generated. Describe any experiments used to assess the effect of a mutation and, where applicable, how potential secondary effects (e.g. second site T-DNA insertions, mosiacism, off-target gene editing) were examined.*

Flow Cytometry

Plots

Confirm that:

☒ The axis labels state the marker and fluorochrome used (e.g. CD4-FITC).

☒ The axis scales are clearly visible. Include numbers along axes only for bottom left plot of group (a 'group' is an analysis of identical markers).

☒ All plots are contour plots with outliers or pseudocolor plots.

☒ A numerical value for number of cells or percentage (with statistics) is provided.

Methodology

Sample preparation	Cell preparation is desribed in full in 'Methods'. After harvesting cell suspensions were normalised to 1x106/ml and stained with non-limiting concentrations of each antibody with full details provided in the Key Resources Table.
Instrument	BD LSR Fortessa

Software	Analysis was undertaken in FlowJo (BD Biosciences) and prism 6.0 (Graphpad)
Cell population abundance	Primary human CD8+ T cells were separated from leucocyte cones obtained from NHS Blood and Transplant (Addenbrooke's Hospital, Cambridge, UK) by centrifugation over ficoll and positive selection using magnetic beads as previously described82. The purity of separated cell subsets was determined by three-colour flow cytometry. Purified T cells (>95%) were labelled with 2.5µM CFSE (Invitrogen) and resuspended in complete RPMI 1640 (Sigma Aldrich) in the presence of 10% FCS and then stimulated in sterile, U-bottomed 96 well culture plates (Greiner) using MACS iBead particles (1:2 bead:cell ratio, Miltenyi) conjugated to anti-CD2/CD3/CD28 in the presence of non-limiting IL2 (10ng/ml, Gibco Life technologies) for 6 days.
Gating strategy	Cells were gated on viability, live/dead discrimination (AquaFluorescent amine-reactive dye, Invitrogen), and by biaxial gating for CD3/CD8 or other populations as indicated. Thresholds for gate application were chosen using FMO controls where applicable.

☒ Tick this box to confirm that a figure exemplifying the gating strategy is provided in the Supplementary Information.
